# A Non-Canonical Function of Zebrafish Telomerase Reverse Transcriptase Is Required for Developmental Hematopoiesis

**DOI:** 10.1371/journal.pone.0003364

**Published:** 2008-10-10

**Authors:** Shintaro Imamura, Junzo Uchiyama, Eriko Koshimizu, Jun-ichi Hanai, Christina Raftopoulou, Ryan D. Murphey, Peter E. Bayliss, Yoichi Imai, Caroline Erter Burns, Kenkichi Masutomi, Sarantis Gagos, Leonard I. Zon, Thomas M. Roberts, Shuji Kishi

**Affiliations:** 1 Schepens Eye Research Institute, Department of Ophthalmology, Harvard Medical School, Boston, Massachusetts, United States of America; 2 Department of Cancer Biology, Dana-Farber Cancer Institute, Harvard Medical School, Boston, Massachusetts, United States of America; 3 Renal Division, Department of Medicine, Beth Israel Deaconess Medical Center, Boston, Massachusetts, United States of America; 4 Stem Cell Program and Division of Hematology/Oncology, Children's Hospital Boston, Howard Hughes Medical Institute, Harvard Medical School, Boston, Massachusetts, United States of America; 5 Laboratory of Genetics, Biomedical Research Foundation of the Academy of Athens Greece, Athens, Greece; 6 The CBR Institute for Biomedical Research, Harvard Medical School, Boston, Massachusetts, United States of America; 7 Cancer Stem Cell Project, National Cancer Center, Tokyo, Japan; Centre de Regulacio Genomica, Spain

## Abstract

Although it is clear that telomerase expression is crucial for the maintenance of telomere homeostasis, there is increasing evidence that the TERT protein can have physiological roles that are independent of this central function. To further examine the role of telomerase during vertebrate development, the zebrafish telomerase reverse transcriptase (zTERT) was functionally characterized. Upon zTERT knockdown, zebrafish embryos show reduced telomerase activity and are viable, but develop pancytopenia resulting from aberrant hematopoiesis. The blood cell counts in TERT-depleted zebrafish embryos are markedly decreased and hematopoietic cell differentiation is impaired, whereas other somatic lineages remain morphologically unaffected. Although both primitive and definitive hematopoiesis is disrupted by zTERT knockdown, the telomere lengths are not significantly altered throughout early development. Induced p53 deficiency, as well as overexpression of the anti-apoptotic proteins Bcl-2 and E1B-19K, significantly relieves the decreased blood cells numbers caused by zTERT knockdown, but not the impaired blood cell differentiation. Surprisingly, only the reverse transcriptase motifs of zTERT are crucial, but the telomerase RNA-binding domain of zTERT is not required, for rescuing complete hematopoiesis. This is therefore the first demonstration of a non-canonical catalytic activity of TERT, which is different from “authentic” telomerase activity, is required for during vertebrate hematopoiesis. On the other hand, zTERT deficiency induced a defect in hematopoiesis through a potent and specific effect on the gene expression of key regulators in the absence of telomere dysfunction. These results suggest that TERT non-canonically functions in hematopoietic cell differentiation and survival in vertebrates, independently of its role in telomere homeostasis. The data also provide insights into a non-canonical pathway by which TERT functions to modulate specification of hematopoietic stem/progenitor cells during vertebrate development. (276 words)

## Introduction

Telomerase is a ribonucleoprotein complex required for the synthesis of telomere terminal repeats. The essential components required for this activity are telomerase reverse transcriptase (TERT), the catalytic component, and telomerase RNA (TR) (or TERC; telomerase RNA component) which is the template for DNA repeat synthesis [1], [2]. Telomerase elongates telomeres and protects chromosome ends from recombination and fusion, and the loss of this enzyme can trigger cellular DNA damage responses in both the presence and absence of altered telomere integrity [1], [3], [4], [5]. The TERT protein is well conserved evolutionarily and has now been characterized with regard to its functional motifs and domains [6], [7] (**Figure S1** **,** **Figure S2A**). The reverse transcriptase (RT) motifs are essential for the enzymatic activity of TERT in synthesizing telomere repeats and also play an important role in nucleotide addition and processivity in concert with its C-terminal domain [8], [9]. With respect to the physical interaction between TERT and TR in vitro, the RT domain of TERT appears to be dispensable [6], [7], [10]. Instead, the RNA-binding domains of TERT interact with TR to facilitate the elongation of the telomere repeats via the catalytic activity of the RT domain [11], [12], [13], [14], [15]. Hence, both the TR binding and RT domains of TERT must act in concert for the synthesis of telomere repeats [13].

Telomerase activity is detectable at different levels in various cell types and correlates with their proliferative potential [16], [17]. In higher vertebrates including humans, telomerase expression is dynamically and precisely regulated in normal somatic tissues, but is constitutively expressed in most cancer cells and long-lived self-renewing cells such as stem cells [18], [19]. Interestingly, rodents and lower vertebrates have relatively loosely regulated or even constitutive telomerase activity in their somatic cells [20], [21], [22], [23], [24]. The biological significance of ‘tight’ versus ‘loose’ regulation of TERT expression in different vertebrates may be associated with the differing stem cell functions, regenerative abilities, and cancer predisposition in various species [25], [26], [27], [28], [29], [30]. However, links between TERT and cellular functions that govern the relationship between telomerase activity, telomere structure, and telomere length have not been elucidated extensively in lower vertebrates, including zebrafish, until recently [22], [31], [32], [33], [34].

Although it is now clear that telomerase expression is crucial for the maintenance of telomere homeostasis, there is increasing evidence that the TERT protein can have physiological roles that are independent of this central function [5], [25], [29], [35], [36], [37], [38], [39], [40]. In tumor-derived cells, TERT promotes tumor development, even if the cells possess telomeres of ample length [38]. This observation implies that TERT has at least one function that is distinct from telomere maintenance during tumorigenesis. Mice have long telomeres and telomere shortening is not an actual barrier to cellular transformation in the absence of TR [41]. Yet in transgenic mice, TERT overexpression promotes stem cell mobilization, hair growth, and stem cell proliferation in the absence of changes in telomere length, and this can occur both dependently and independently of TR expression [27], [29]. Moreover, ablation of human TERT (hTERT) expression affects the overall configuration of chromatin, and abrogates the cellular response to DNA double strand breaks, without altering telomere integrity [5]. These results support the notion that TERT may have non-canonical functions, although the underlying mechanisms by which TERT operates in this way remain unclear.

Genetically engineered mice lacking TR are viable, but telomere loss and increased end-to-end fusions have been reported in later generations [17]. The phenotypes associated with telomere dysfunction include neural tube defects, severe intestinal atrophy, reduced angiogenic potential, and reduced proliferative potential of the bone marrow stem cells [42]. Furthermore, TERT-deficient mice have also been generated [43], [44], and active telomerase in these animals appears to be critical for telomere maintenance as obvious telomere shortening was evident in comparably later generations [43], [45], [46]. In contrast, Chiang et al. [47] have reported that *mTERT^+/−^* heterozygotes had no detectable defects in telomere elongation compared with wild-type controls. In addition, Yuan et al. [44] previously observed that there were no significant changes in G-strand 5′-overhangs between *mTERT^+/+^*, *mTERT^+/−^*, and *mTERT^−/−^* mice, at least in the early generation progeny.

In humans, heterozygous mutations of the human TR (hTR) gene have been described in patients with acquired aplastic anemia and the autosomal dominant form of dyskeratosis congenita. Dyskeratosis congenita is a rare skin and bone marrow failure syndrome caused by defective telomere maintenance in hematopoietic stem cells [48]. More recently, heterozygous mutations have also been identified in hTERT among aplastic anemia patients [49]. These results suggest that partially impaired telomerase activity arising from a haploinsufficiency might induce bone marrow failure in humans. Mice deficient in the telomerase gene products are well established and potentially very good models to study the pathogenesis of telomerase-related bone marrow failure, as already reported [50]. However, the choice of animal model remains one of the most important issues for a variety of experimental approaches to the study of telomerase. In addition, given the possibility that mice models may not recapitulate all of the phenotypes of human bone marrow failure patients, it will also be important to examine the function of telomerase in other animal models.

Zebrafish (*Danio rerio*) is an excellent vertebrate model for studying developmental hematopoiesis [51]. The embryos of this fish are transparent and develop rapidly ex-utero, thus allowing for easy observation of multiple organs, including the vasculature and the relative number and color of circulating blood cells. Zebrafish orthologs for genes expressed in many mammalian blood cell types have also been identified [52], [53]. Moreover, a number of zebrafish mutants have now been developed as models for human hematopoietic diseases, such as congenital dyserythropoietic anemia, sideroblastic anemia, hepatoerythropoietic porphyria, hemochromatosis, and myelodysplastic syndrome (MDS) [54], [55], [56]. Although the critical roles of telomerase in hematopoietic cells have been documented extensively in the literature [57], [58], little is known about the functional involvement of TERT in the molecular programming of embryonic hematopoiesis in vertebrates.

In our current study, we have isolated zebrafish TERT (zTERT) and characterized its functional roles in hematopoiesis during early development. We show that zTERT knockdown causes hypochromic anemia at the onset of circulation, and that this is accompanied by the impaired differentiation of blood cells and their eventual apoptotic cell death leading to a severe reduction of hematopoietic cells (‘pancytopenia’), during embryogenesis. The phenotypes resulting from zTERT deficiency bear many of the hallmarks of MDS, rather than of aplastic anemia. In addition, although peripheral blood cytopenia was observed, we also detected dysplasia of blood cell development during hematopoiesis. Based on the alterations of hematopoietic cell differentiation markers, TERT deficiency in zebrafish may cause differentiation and maturation failure in both primitive and definitive hematopoiesis. The cytopenic phenotype, but not impaired differentiation, in the zTERT-deficient embryos is significantly reduced by the loss of p53 as well as by the expression of the anti-apoptotic proteins Bcl-2 and E1B-19K. Intriguingly, the effects observed in zTERT deficient embryos appear to be independent of the telomere maintenance function of telomerase, and can be compensated by the overexpression of a telomerase activity-negative deletion mutant of zTERT lacking the TR-binding domain. Taken together, our results demonstrate for the first time that zTERT promotes the development of hematopoietic cells through a non-canonical mechanism that is independent of the authentic telomerase activity of TERT and the role of this enzyme in telomere lengthening. Our zebrafish model should therefore provide new platforms with which to examine novel TERT functions and pathways related to human hematopoietic disorders.

## Results

### Induction of apoptosis, but not telomere shortening, by the ablation of TERT in zebrafish embryos

Sequence analysis and comparisons have confirmed a high degree of homology between the functional domains of zTERT and human TERT (hTERT). The N-terminus, TR-binding site, and RT motifs are the most highly conserved regions. The zTERT protein shows approximately 50% identity with its hTERT counterpart within these functional domains, but exhibits only a 22% identity outside these regions. Importantly, the metal-binding motifs in RT motifs A and C of TERT, which are responsible for the two-metal mechanism underlying its catalytic activity, are conserved in zTERT (**Figure S1**). In addition, the overall primary structure of zTERT, based on its amino acid sequence, has a high degree of homology to that of human, mouse, chicken, *Xenopus*, and Fugu TERT (**Figure S1** **,** **Figure S2**). zTERT is also significantly expressed during early development and also in multiple tissues in adult fish (**Figure S3**) [23].

**Figure 1:**
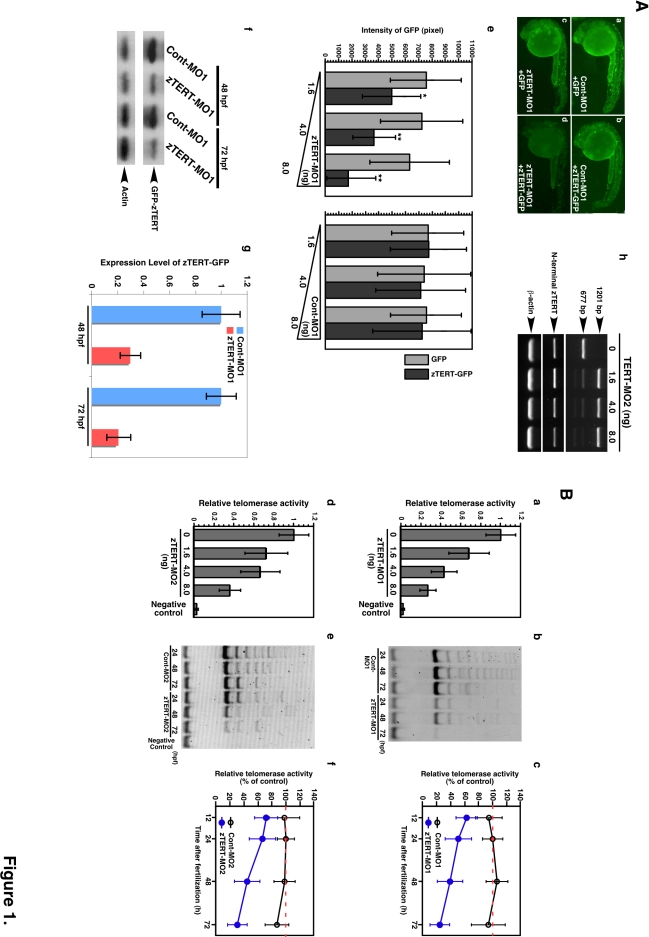
The knockdown of TERT in the zebrafish embryo does not result in telomere shortening. (A) A zTERT-MO1-induced translational block was monitored using mini-zTERT-GFP (zTERT-GFP) in vivo. To test the effectiveness of the MO1 in binding the transcript and inhibiting downstream translation, a GFP reporter construct driven by a DNA sequence upstream from the *zTERT* coding region (encompassing the zTERT-MO1 target region) was used. Representative GFP expression profiles are shown for embryos (24 hpf) injected with Cont-MO1 and GFP empty vector (a), Cont-MO1 and zTERT-GFP (b), zTERT-MO1 and GFP empty vector (c), and zTERT-MO1 and zTERT-GFP (d). (e) Quantification of the GFP intensity in embryos injected with a combination of MOs (Cont-MO1 or zTERT-MO1) and plasmids (GFP or zTERT-GFP) as shown in Figure 1A. Five independent experiments incorporating more than 50 embryos in each instance were performed. ^*^ *P*<0.01, ^**^ *P*<0.001, (Student t-test). (f) Western blot analysis using an anti-GFP antibody to detect the expression of zTERT-GFP, and its reduction by zTERT-MO1 but not Cont-MO1. (g) For the western blotting results, the intensities of the bands were quantitated using the associated pixel levels and the expression levels of GFP-zTERT were normalized to actin in each time point as a ratio of mean values which are shown in the right side graphs. These experiments were independently performed three times. *P*<0.001 (Student t-test). (h) RT-PCR analysis of zTERT MO2-induced altered splicing of zTERT transcripts. The 677 bp intact zTERT transcript was detectable in uninjected embryo samples (0 ng), and to some degree in the 1.6 and 4.0 ng injected samples. A 1201 bp product indicating the insertion of an intact intron between exons 5 and 6 was almost predominantly observed in samples injected with 8 ng MO2 (48 hpf). In contrast, the 677 bp intact band was almost undetectable in these same morphants. (B) Quantitative analysis of telomerase activity in zTERT knockdown embryos injected with 1.6 to 8.0 ng MO1 and MO2 during 12–72 hpf. (a, d) A TRAP-ELISA assay was performed in zTERT knockdown embryos injected with 1.6, 4.0, and 8.0 ng MO1 (a) or MO2 (d). (b, e) TRAP assay using electrophoretic gel analysis over a 24–72 hpf timecourse for MO1 (b) or MO2 (d). (c, f) Quantitative fluorometric TRAP assay performed over a 12–72 hpf timecourse for MO1 (c) or MO2 (f).

To analyze the functional roles of the TERT protein during vertebrate development, we performed knockdown analysis of the zTERT gene. To this end, we designed a morpholino antisense oligonucleotide (MO) targeting the translation initiation codon and 5′-UT region of the zTERT gene to block translation of zTERT mRNA (zTERT-MO1), and injected this MO into zebrafish embryos at the 1–2-cell stage. As an antibody that specifically recognizes or cross-reacts with the zTERT protein is not currently available, we generated a construct containing the translational initiation site of zTERT fused with green fluorescent protein (GFP) as a transgene (in mimicry of zTERT) for introduction into zebrafish embryos to demonstrate that our zTERT-MO1 works in vivo (**Figure S4A**). The resulting transgenic embryos allowed us to monitor the suppression of extrinsic mimic zTERT (zTERT-GFP) expression by MO1 through the analysis of the GFP expression levels (**Figure 1A, a–d** **,** **Figure S4B**). We also performed western blot analysis using an anti-GFP antibody to detect the expression of the zTERT-GFP protein, and its reduction by zTERT-MO1 but not Cont-MO1 (**Figure 1A, f, g**).

We next determined whether zTERT-MO1 inhibits telomerase activity in vivo using the standard telomere repeat amplification protocol (TRAP) assay. A decrease in telomerase activity by this MO was found to be dose-dependent, as revealed by injection of 1.6 to 8 ng/embryo (**Figure 1B, a**; TRAP-ELISA assay). **Figures 1B, b** (TRAP-gel loading assay), **c** (Fluorometric TRAP assay) show the enzymatic activity of telomerase in lysates from 12, 24, 48 and 72 hours post fertilization (hpf) embryos. At each point over this time course, more decreases in activity with time were detectable after the injection of zTERT-MO1 (8 ng/embryo). In contrast, injections of two types of control MOs (a 5-base mismatch MO1 as the Cont-MO1 and an inverse-sequence MO1 for the sequence of zTERT-MO1) had no significant effects on telomerase activity (**Figure 1B, b, c**; not shown for the inverse-sequence MO1).

An additional zTERT-specific MO (zTERT-MO2) was also designed that creates aberrant splicing between exon 5 and exon 6 of this gene (**Figure S4B**). RT-PCR and DNA sequencing results showed that the intron between exon 5 and exon 6 was not skipped out during splicing in MO2-injected embryos (**Figure 1A, h**). More specifically, integration of this intact intron created an in-frame premature stop codon (TGA), resulting in a truncated protein lacking most of the reverse transcriptase domain (termed motifs A, B', C, D, and E, and further carboxyl-terminal end region) (**Figure S4B**, see also the zTERT structure in **Figure S1**, **Figure S2A**). Hence, this splice-blocking MO2 successfully generated a telomerase activity-defective zTERT product (**Figure 1B, d–f**), as measured by the three different types of the TRAP assay methods.

We next measured the telomere lengths in zebrafish embryos by whole-mount quantitative fluorescence in situ hybridization (WM-Q-FISH). TERT-deficient and control embryos at 24 hpf were hybridized with a Cy3-labeled peptide nucleic acid (PNA) telomere-specific probe as described in the Materials and Methods [59], [60]. Telomere speckles in interphase nuclei can be easily visualized and quantified by WM-Q-FISH throughout the body of the test animals. Magnified images of these speckles were then used to quantify the relative telomere lengths. We compared zTERT morphants and control animals by measuring the telomere lengths in the eyes, brains, and muscles of the injected embryos by WM-Q-FISH. No decrease in the telomere lengths was detectable in any of these tissues at 24–72 hpf after injection of zTERT-MO1 or zTERT-MO2 (data not shown). Moreover, we performed metaphase chromosome spreading to enable telomere FISH (**Figure S5A, B**), in addition to the originally performed interphase telomere FISH. We then compared not only average telomere lengths, but also determined the number of critically short telomeres and signal-free telomere ends. We found populations of cells that exhibited no critical telomere shortening, and there were no statistically significant differences between the corresponding low fluorescent signals from either the MO1- or MO2-injected embryos when compared with the controls (**Figure S5B**). These findings significantly reduced the likelihood that short telomeres play any role in propagating the cell fate abnormalities that we observe in the TERT-knockdown zebrafish embryos.

We also measured telomere lengths by terminal restriction fragment (TRF) Southern blotting after digestion of the genomic DNA with the *Hinf I* and *Rsa I* restriction enzymes and hybridization with telomere probes. The mean zebrafish telomere length was determined to be 15–20 kb in embryos and larval fish as well as in young adult fish (**Figure S5C**). Consistent with our earlier Q-FISH results, the TRF lengths were found to be unchanged in the zTERT-MO embryos (**Figure S5C**). To verify that telomere length changes were indeed detectable by TRF Southern blotting, we performed *in vitro* TRF shortening by *DNase I* nuclease treatment of zebrafish genomic DNA (**Figure S5C, a**), as compared with longer (high) and shorter (low) human telomeres (**Figure S5C, b**). We likewise checked the integrity of the 3′ G-strand overhangs, but did not find significant differences between the zTERT knockdown embryos and the controls (data not shown). It thus appears that the inhibition of telomerase in the zTERT morphants is not sufficient to elicit detectable telomere shortening during the early developmental stages of zebrafish embryos.

**Figure 2:**
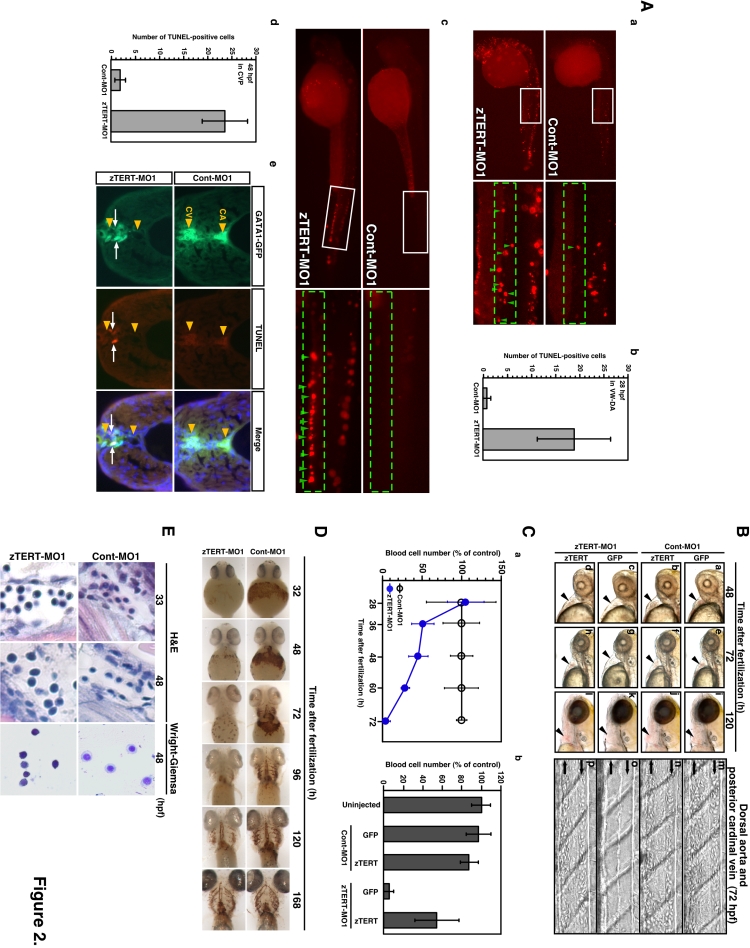
The knockdown of TERT in the zebrafish embryo results in severe cytopenia and in the impaired differentiation of hematopoietic cells. (A) Induction of apoptosis in hematopoietic cells of the zebrafish embryo. Both a low magnification of the whole body and higher magnification of the trunk region are shown in (a) and (c), respectively. White squares in the low magnification images designate the regions shown in the higher magnification images in the adjoining right panels. (a) Apoptotic cells were detected by a TUNEL assay of the ventral wall of the dorsal aorta (VW-DA) (shown by green arrow heads in the right panel) at 28 hpf. (b) Quantification of the TUNEL-positive cells in the ventral wall of dorsal aorta (VW-DA) at 28 hpf. The number of TUNEL-positive cells was estimated within the gated area indicated by the green dashed rectangle at the upper yolk extension. (c) Apoptotic cells were detected by a TUNEL assay of the caudal venous plexus (CVP) (shown by green arrow heads in the right panel) at 48 hpf. (d) Quantification of the TUNEL-positive cells detected in the CVP at 48 hpf. The quantity of TUNEL-positive cells was assessed.within the gated area indicated by the green dashed rectangle at the anatomical CVP region. (e) Detection of apoptosis in *gata-1^GFP^*-positive hematopoietic cells. Transverse sections through the trunk region of 48-hpf *gata-1^GFP^* embryos with the dorsal up are shown. The caudal artery (CA; upper) and caudal vein (CV; lower) are shown by orange arrow heads in the panels. By TUNEL assay, *gata-1^GFP^*-positive apoptotic cells in the CV are evident and indicated by white arrows. (B) Lateral views of 48, 72 and 120 hpf embryos following the co-injection of zTERT-MO1 (or Cont-MO1) and GFP-zTERT-cDNA (or GFP-cDNA) expression vectors (a–l); black arrowheads indicate the heart regions. Bright field pictures of blood cells in trunks of 72 hpf embryos after co-injection of zTERT-MO1 or Cont-MO1 and either a GFP-cDNA or GFP-zTERT-cDNA vector (m–p). The upper vessel is the dorsal artery (from left to right arrows) and the lower vessel is the posterior cardinal vein (from right to left arrows). (C) Quantitation of the circulating blood cell number in zTERT-MO- (blue circle) versus Cont-MO- (black open circle) injected embryos during 28–72 hpf (a). (b) Calculation of the percentage of the control circulating blood cell numbers at 72 hpf after co-injection of zTERT-MO or Cont-MO and either a GFP-control or GFP-zTERT-cDNA vector. Blood cell numbers were determined for 10 embryos from each group. (D) Whole-mount o-dianisidine staining for heme detection in uninjected, Cont-MO1- and zTERT-MO1-injected embryos during 32–168 hpf. Blood flow over the yolk sac and in the tail vessels results in brown staining in wild type (data not shown) and Cont-MO1-injected embryos during 32–168 hpf (ventral view). (E) H&E staining of blood cells in tissue sections of the arteries or veins of Cont-MO1- and TERT-MO1-injected embryos at 33 and 48 hpf, and Wright-Giemsa staining of isolated blood cells from Cont-MO1- and TERT-MO1-injected embryos at 48 hpf.

With regard to the morphological phenotypes associated with a zTERT knockdown, zTERT-MO-injected embryos appear normal throughout embryogenesis, although a slight growth retardation was observed until 24 hpf (data not shown). However, after 24 hpf, TERT morphants do not display marked gross morphological changes, and their growth and development proceeds at the almost the same rate as in the control animals. We compared the number of apoptotic cells in zTERT-MO-injected embryos with wild-type animals. Accordingly, in zTERT morphants, TUNEL (terminal deoxynucleotidyl transferase biotin-dUTP nick end-labeling)-positive apoptotic cells can be observed throughout the head and trunk and in the ventral wall of the dorsal aorta (VW-DA) where the first definitive hematopoietic stem cells presumably emerge at 28 hpf (**Figure 2A, a, b**), with a gradual reduction after this time period. However, at 48 hpf (up to 72 hpf) in the caudal venous plexus (CVP), TUNEL-positive cells are detectable again (**Figure 2A, c, d**). The detection of TUNEL-positive cells in zebrafish *gata-1*-promoter-driven GFP (*gata-1^GFP^*) transgenic fish at 48 hpf revealed that apoptotic cells were indeed involved in the blood cell population (**Figure 2A, e**). These results thus suggest that a zTERT deficiency causes impaired hematopoiesis.

### Induction of severe cytopenia and the impaired differentiation of hematopoietic cells by zTERT knockdown in zebrafish embryos

In contrast to the relatively normal morphological development of the zTERT morphants, striking effects were observed in the hematopoietic pathways in these embryos. Primitive blood cell circulation becomes visible by 28 hpf in both zTERT morphants and control embryos, whereas light microscopy reveals hypochromic blood in zTERT morphants at 32 hpf (see the heme intensity in **Figure 2D**). During 36–72 hpf, zTERT morphants became increasingly anemic with erythrocytes becoming undetectable by 72 hpf (**Figure 2B–D**). From 60 hpf, circulating blood cells are dramatically decreased in the heart and blood vessels of zTERT-MO1-injected embryos (**Figure 2B, g, k, o**). At 72 hpf, Cont-MO1-injected embryos contain an average of 125 cells per 0.5 mm of dorsal aorta, whereas most zTERT-MO1-injected embryos show less than 5% of these numbers in the same area (**Figure 2C**). The decrease of circulating blood cells in TERT-deficient animals continued up to 5 days post fertilization (dpf), but subsequently recovered by 7 dpf, due to the transient knockdown of zTERT using MO in this system (data not shown). zTERT-MO2 elicited this same spectrum of blood cell phenotypes (**Figure S6A–E**).

We next examined whether the overexpression of zTERT could rescue the blood cell number and differentiation phenotypes in our system, hence relieving the embryonic cytopenia and anemia induced by zTERT-MO1 and -MO2. Microinjection of a GFP-tagged wild-type zTERT (GFP-zTERT) expression vector did not cause any morphological abnormalities (data not shown). Co-injections of zTERT-MO1 or -MO2 together with either the GFP-zTERT or control GFP vector were then performed. **Figure 2B** shows the blood circulation in the heart area (lateral view, arrowhead indicates the heart). A red color that is indicative of normal chromic blood cell circulation in the heart was not observed at 48 and 72 hpf in TERT-deficient embryos (**Figure 2B, c, g**). TERT-deficient animals were significantly rescued from this decrease of blood cell circulation following the co-expression of a zTERT vector, but not by a control GFP vector, at 72 hpf (**Figure 2B, g, h, o, p**). Notably, the overexpression of zTERT in Cont-MO-injected embryos did not induce any significant changes such as over-proliferation of blood cells (**Figure 2B, C, b**). These results provide strong evidence that both anemia and cytopenia induced by two different zTERT-MOs (MO1 and MO2) is caused by the specific inhibition of zTERT function (see **Figure S9** for MO2).

We speculated that blood progenitor cells in TERT-deficient animals may not differentiate appropriately into normal mature erythrocytes. We therefore analyzed, using whole-embryo staining with o-dianisidine, whether the red blood cells present in TERT-deficient animals were sufficiently differentiated to become hemoglobinized. We confirmed that there was a severe reduction in the erythroid hemoglobin content in TERT-knockdown embryos compared with control embryos from 32 to 96 hpf but that these levels subsequently recovered by 7 dpf, due to the transient nature of this knockdown system (**Figure 2D** for MO1, **Figure S6C, D** for MO2). Moreover, the intravenous microinjection of iron-dextran failed to rescue this impaired hemoglobin production (data not shown), indicating that inadequate levels of circulatory iron cannot account for the hypochromia of TERT-deficient embryos. These results suggest that the circulating erythrocytes in TERT-deficient embryos are likely to still be immature and have defects in hemoglobin synthesis and/or production. This hypothesis was confirmed when we analyzed the blood cells in the artery and veins by hematoxylin and eosin (H&E) staining of tissue sections at 33 and 48 hpf. The blood cells in zTERT morphants appeared blast-like, with large nuclei characteristic of immature erythrocytes (**Figure 2E**). Wright-Giemsa staining of isolated blood cells at 48 hpf also revealed inefficient development of erythrocytes having a blastic (immature) phenotype (**Figure 2E** for MO1, **Figure S6E** for MO2). Taken together, these results indicate that TERT function is likely to be required for progenitor blood cell differentiation, and could also be required for their specification, maturation, and survival. At 72 hpf, although peripheral blood cytopenia was obviously detectable, some non-circulating blood cells still existed around the CVP, as evident from a transverse section of a zTERT morphant (data not shown). This suggests that phagocytosis followed by apoptotic cell death due to ineffective hematopoiesis is still actively occurring in these areas.

### The involvement of zebrafish TERT in both primitive and definitive hematopoiesis

**Figure 3:**
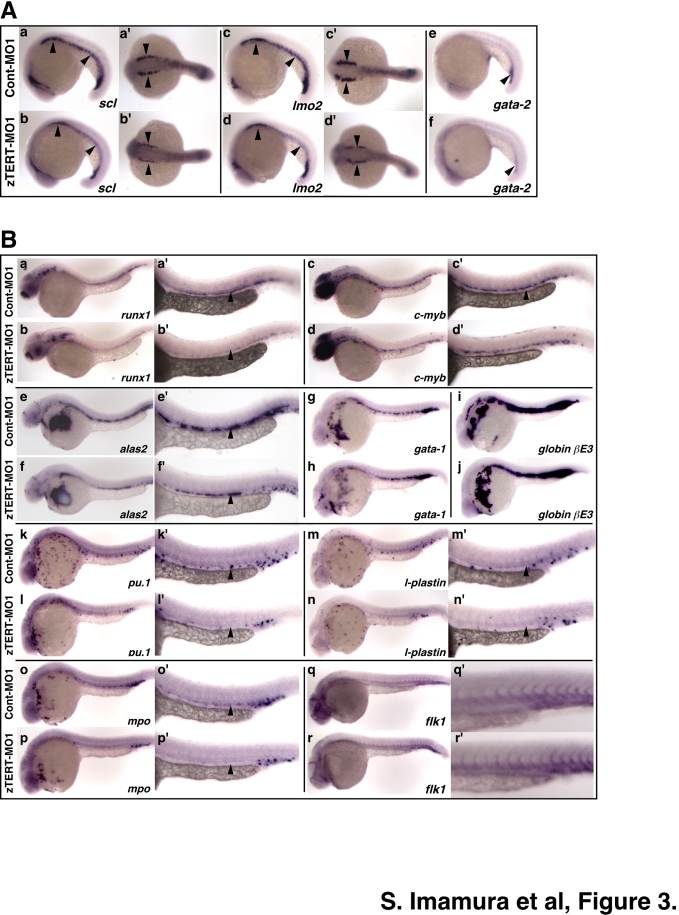
TERT is required for both primitive and definitive hematopoiesis in the zebrafish. Whole-mount in situ hybridization of control and TERT deficient zebrafish embryos at the 20-somite stage (19 hpf) (A), and at 28–32 hpf (B). (A) For the detection of primitive hematopoietic markers, 20-somite stage zebrafish embryos (19 hpf) are shown and are oriented anterior left in lateral (a–f) and dorsal views (a'–d'). Control and TERT morphants were analyzed for the expression of *scl*, *lmo2* and *gata-2*, which are early hematopoietic markers. The expression of *scl* and *lmo2* in the ICM (a–d, a'–d'), and that of *gata-2* in the blood island (e, f), is indicated by arrowheads. (B) Embryos at 28–32 hpf and oriented anterior left in a lateral view and are shown at a low (a–r) and high (a'–r') magnification of the trunk region. Control and TERT morphants were analyzed for the expression of multiple hematopoietic cell lineage markers. Representative time points for the expression of *runx1* (32 hpf), *c-myb* (32 hpf), *alas2* (32 hpf), *gata-1* (28 hpf), *pu.1* (28 hpf), *globin bE3* (28 hpf), *l-plastin* (28 hpf), *mpo* (28 hpf), and *flk1* (28 hpf) genes are shown. The expression in the arterial region is indicated by arrowheads (a'–n').

To examine the requirement of the *TERT* gene during developmental hematopoiesis in zebrafish, the expression of multiple marker genes for both primitive and definitive hematopoiesis was analyzed by in situ hybridization. To evaluate the requirement for TERT for primitive hematopoiesis in zebrafish, we analyzed early hematopoietic markers in the anterior lateral mesoderm (ALM) which produces myeloid cells, and the posterior lateral mesoderm (PLM) that produces erythroid cells [53]. At 19 hpf (20-somite stage), the expression of stem cell leukemia (*scl*), which is known to form a multimeric complex with *lmo2* and *gata-2*, indicates the initiation of hematopoietic stem cell formation [61]. These early hematopoietic markers (*scl*; n = 80 of 85; 94%, *lmo2*; n = 81 of 88; 92%, *gata-2*; n = 54 of 54; 100%) were reduced in the ALM and PLM of the zTERT morphants (**Figure 3A, a–f, a'–d'**) at 19 hpf, but almost recovered at the normal levels by 24 hpf (data not shown). Thus, both the ALM and PLM regions are significantly but temporally affected by a zTERT knockdown suggesting that hematopoietic cell specification is disturbed at least in part.

Both *c-myb* and *runx1* are expressed in definitive hematopoietic stem and progenitor cells in the ventral wall of the zebrafish dorsal aorta [62], [63]. Both of these markers are diminished, however, in TERT-deficient embryos from 32 hpf (*c-myb*; n = 56 of 57; 98%, *runx1*; n = 68 of 69; 99%)–36 hpf (*c-myb*; n = 67 of 70; 96%, *runx1*; n = 75 of 79; 95%) (**Figure 3B, a–d, a'–d'**, data not shown for 36 hpf). On the other hand, the expression of *flk1*, a marker of vasculature endothelial cells, appears to be relatively normal in comparison with control embryos during 24 hpf (n = 51 of 55; 93%) to 28 hpf (n = 45 of 50; 90%) (**Figure 3B, q, r, q', r'**, data not shown for 24 hpf). These results indicate that definitive hematopoietic stem and progenitor cells may be disrupted by a zTERT deficiency, whereas vasculature endothelial cells appeared to be unaffected, during early zebrafish development. We further demonstrated that the vasculature system is intact in zTERT morphants using a vasculature-specific ETS-domain transcription factor *fli-1* promoter-driven GFP-transgenic (*fli* ^GFP^) zebrafish combined with microangiography (**Figure S8**).

Primitive erythrocytes expressing the *gata-1* and *globin βE3* genes appear to be normal in the intermediate cell mass (ICM) of 28 hpf (*gata-1*; n = 52 of 55; 95%, *globin βE3*; n = 64 of 68; 94%) to 30 hpf (*gata-1*; n = 54 of 60; 90%, *globin βE3*; n = 54 of 59; 92%) TERT-deficient zebrafish embryos when compared with control embryos (**Figure 3B, g–j**). In contrast, the expression of delta-aminolevulinate synthase (*alas2*), which is the enzyme required for the first step in heme biosynthesis, was found to be decreased from 26 hpf (n = 41 of 41; 100%) to 32 hpf (n = 50 of 54; 93%) (**Figure 3B, e, f, e', f'**, data not shown for 26 hpf). This is consistent with the hypochromic anemia phenotype showing reduced o-dianisidine staining of the erythroid hemoglobin in TERT-deficient embryos (**Figure 2D**). Moreover, the expression of *pu.1*, *l-plastin* and myeloperoxidase (*mpo*), which is confined to the primitive myeloid cells, is decreased in each case in the ICM of zTERT morphants at 28 hpf (*pu.1*; n = 42 of 45; 93%, *l-plastin*; n = 39 of 40; 98%, *mpo*; n = 50 of 55; 91%) (**Figure 3B, k–p, k'–p'**). Taken together, these results suggest that TERT is involved in both definitive and primitive hematopoietic waves, which may therefore be responsible for the subsequent pancytopenia in TERT deficient zebrafish embryos. In addition, the expression patterns of these multiple hematopoietic markers in TERT-deficient embryos injected with zTERT-MO2 were similar to the zTERT-MO1-injected embryos (**Figure S7A, B**).

We further analyzed whether circulating blood cells cause apoptosis in zTERT-MO-injected embryos. By AO treatment of living TERT-deficient zebrafish embryos, we could not detect flowing AO-positive blood cells at 36, 48, and 72 hpf (data not shown). However, as we described above, in fixed TERT-deficient embryos, TUNEL-positive apoptotic cells were observed at the ICM at 28 hpf (**Figure 2A, a, b**) and at the CVP at 48 hpf (**Figure 2A, c, d**). Moreover, the detection of TUNEL-positive cells in zebrafish *gata-1^GFP^* transgenic fish at 72 hpf revealed that apoptotic cells were indeed present in the blood cell populations (**Figure 2A, e**). The CVP area, in addition to the posterior ICM and aorta-gonad-mesonephros (AGM), has been reported to be the region where both hematopoietic progenitors and phagocytic cells exist [64]. Therefore, these stationary cells may include both immature blood cells excluded from circulation and also dying progenitors. The decreased numbers of GFP-positive cells detectable in zTERT-deficient *CD41*-promoter-driven GFP (*CD41^GFP^*)-transgenic fish support this notion because CD41 is presumably expressed on early hematopoietic stem cells and progenitors during embryonic development (**Figure S7C**) [65], [66], [67], [68]. These data thus suggest that the defective hematopoiesis resulting from TERT deficiency is associated with the ineffective differentiation as well as the insufficient specification of hematopoietic stem/progenitor cells.

### Telomere lengths are maintained in the circulating blood cells of TERT-deficient zebrafish embryos

**Figure 4:**
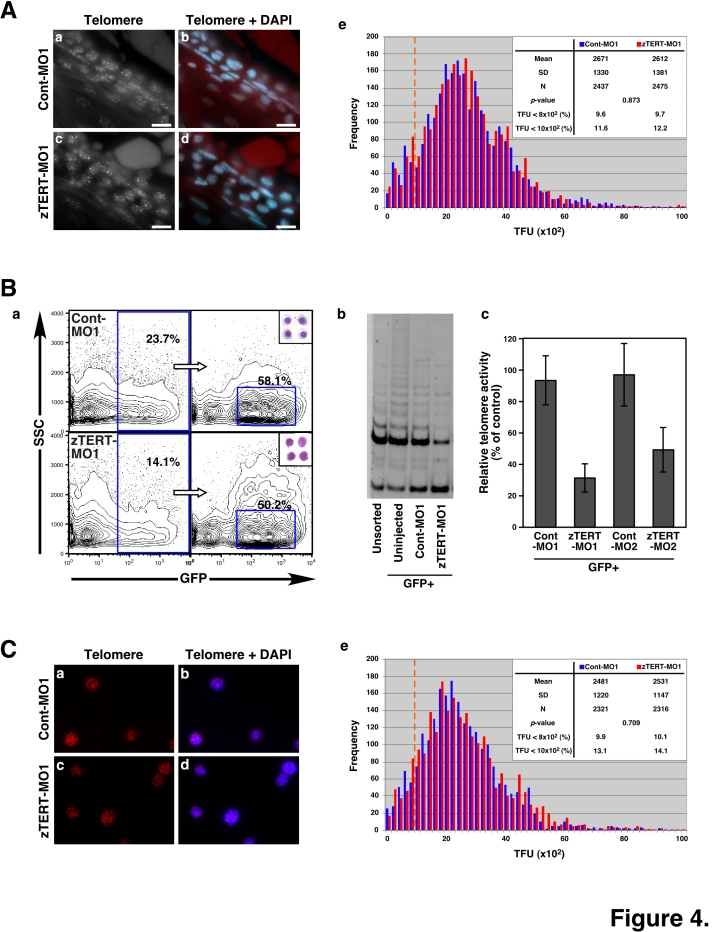
Telomere length and telomerase activity in the blood cells of zebrafish embryos. (A) Telomere length analysis of blood cells in tissue sections from control and TERT morphant embryos by telomere FISH (a, c). The sections were also counterstained with DAPI (b, d). The intensities of the fluorescent speckles detected with a telomere PNA probe reflect the corresponding telomere lengths (a, c; grayscale; bar, 10 µm). Nuclei were counterstained with DAPI and merged with telomere spots on the captured images (b, d; bar, 10 µm). (e) Q-FISH histograms showing telomere fluorescence in blood cell nuclei from control (the blue color bars) and TERT morphants (the red color bars), as measured using the TFL-TELO software. The x-axis depicts the intensity of each signal as expressed in telomere fluorescence intensity units (TFU), and the y-axis shows the frequency of telomeres of a given intensity. The dashed orange line indicates 10×10^2^ TFU. (B) Zebrafish erythroid cells were isolated from *gata-1*-promoter-driven GFP (*gata-1^GFP^*) transgenic fish by FACS (a). TRAP assay of telomerase activity in *gata-1^GFP^*-posiitve blood cells (b). Quantification of telomerase activity by captured image analyses in GFP-positive cells from the MO1- and MO2-injected samples (c). The activity in GFP-positive cells from uninjected samples was used as a control. (C) Telomere length analysis of *gata-1^GFP^*-positive blood cells from control and TERT morphant embryos by telomere FISH (a, c). The intensities of the fluorescent speckles detected with a telomere PNA probe reflect the corresponding telomere lengths. Nuclei were counterstained with DAPI and merged with telomere spots on the captured images (b, d; bar). (e) Q-FISH histograms showing telomere fluorescence in blood cell nuclei from control (the blue color bars) and TERT morphants (the red color bars), as measured using the TFL-TELO software. The x-axis depicts the intensity of each signal as expressed in telomere fluorescence intensity units (TFU), and the y-axis shows the frequency of telomeres of a given intensity. The dashed orange line indicates 10×10^2^ TFU.

We speculated that the loss of circulating blood cells in our zebrafish morphants may be caused by specific and critical telomere shortening, with associated telomere attrition and cellular crisis, resulting from the induced zTERT deficiency. To test this possibility, the telomere lengths were analyzed at the single cell level using Q-FISH. At 36–48 hpf, sections of vessels including the circulating blood cells were hybridized with a telomere probe (**Figure 4A, a–d**). Other tissues were also compared in terms of telomere fluorescence intensities in control and zTERT morphants (data not shown). The telomere lengths in the blood cells of TERT-deficient embryos were found to be similar to those of the control embryos. Moreover, in all of the examined tissues such as the eye, brain and muscle (data not shown), as well as in blood cells, no significant differences in the telomere lengths could be observed between zTERT morphants and control animals (**Figure 4A, e**).

In order to analyze telomere lengths more specifically in the erythroid cell lineage, we also utilized *gata-1^GFP^*-transgenic fish [69]. Since endogenous *gata-1* expression was found to be unchanged in our zTERT morphants (**Figure 3B, g, h**), it was possible to trace the erythroid cell lineage using *gata-1^GFP^*-transgenic fish. The *gata-1^GFP^*-positive fluorescent cells were localized in the ICM at 28 hpf and no critical differences in cell numbers were observed between the control and zTERT morphants (data not shown) [66]. These fluorescent cells were isolated from living embryos by cell sorting (FACS) at 48 hpf when blood cells in TERT-deficient embryos are still present (**Figure 4B, a**). Circulating blood cells, in control *gata-1^GFP^*-transgenic fish are visible by GFP at 4 dpf, at a time when no circulating *gata-1^GFP^*-positive cells can be observed in blood vessels of the zTERT morphants (data not shown). However, some uncirculated GFP-positive cells still existed around the CVP in the zTERT knockdown embryos (data not shown). Impairments of the vasculature, such as vascular leaks and malformations, were also not observed by microangiography in the zTERT morphants at any time during the period of observation (32–96 hpf) (**Figure S8** for 72 hpf). To demonstrate the effects of a zTERT knockdown in *gata-1^GFP^*-positive cells, the sorted cells were subjected to a TRAP assay (**Figure 4B, b, c**). The cells sorted from TERT-deficient embryos show immature erythrocyte morphology (insets of **Figure 4B, a**), and have reduced telomerase activity in comparison with untreated and control embryos (**Figure 4B, b, c**). Nevertheless, we still did not detect any zTERT deficiency-induced telomere shortening by telomere length Q-FISH analysis in smears of *gata-1^GFP^*-positive erythroid cells (**Figure 4C**). These results suggest that TERT is involved in blood cell differentiation during early development irrespective of the telomere length and maintenance status of these cells.

### A p53-deficiency prevents a zTERT deficiency-induced blood cell decrease in the zebrafish embryo

**Figure 5:**
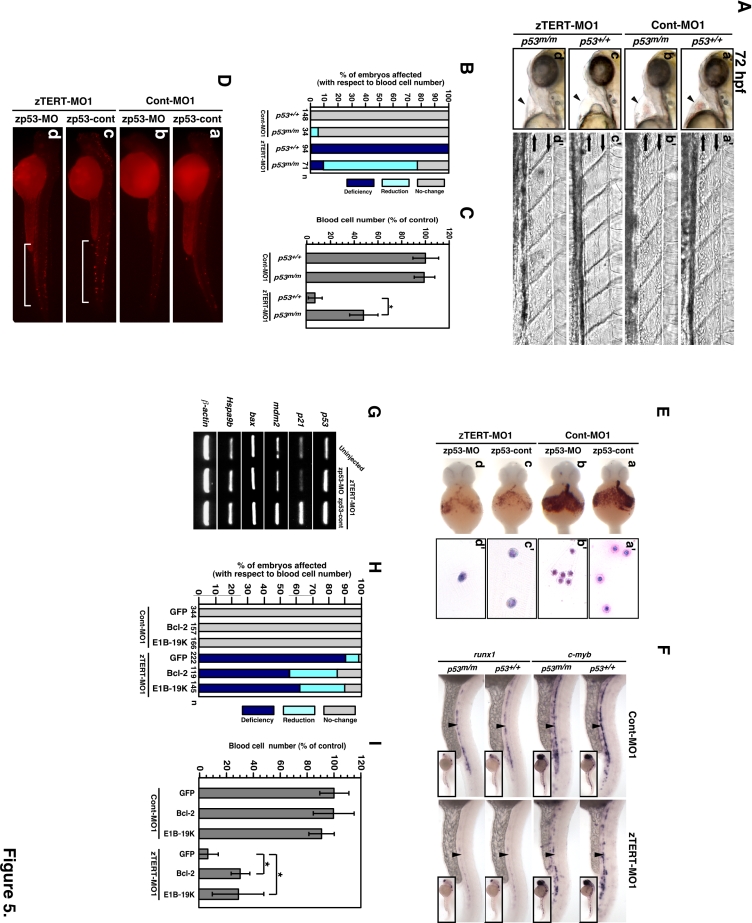
Rescue of cytopenia, but not anemia, in zTERT morphant embryos with a p53-deficient background. (A) Lateral views (anterior to left) of wild-type (*p53^+/+^*) and homozygous *p53^M214K^* mutant (*p53^m/m^*) embryos injected with TERT-MO1 (a–d). Arrowheads indicate the heart regions, including the blood (a'–d') and views of the artery and veins (anterior to left) in the trunk at 72 hpf. (B) Scoring system based on the number of circulating blood cells at 72 hpf after injection of zTERT-MO1 or Cont-MO1 into *p53^+/+^* and *p53^m/m^* embryos. We divided the embryos into three classes based on their flowing blood cell number: i) indistinguishable from the control (>90%; no change) as indicated by the gray bar, ii) cell number reduction compared with the control (10–90%; reduction) as indicated by the light-blue bar, and iii) severely deficient or almost no flowing blood cells (<10%; deficiency) as indicated by the dark-blue bar. (C) Percentages of the control levels of circulating blood cell numbers at 72 hpf after the injection of zTERT-MO1 or Cont-MO1 into *p53^+/+^* and *p53^m/m^* embryos. Blood cell numbers were counted in 10 embryos from each group. ^*^ *P*<0.01 (Student t-test). (D) Whole-mount TUNEL staining in control and zTERT-knockdown embryos coinjected with either zp53-MO- or zp53-control-MO at 48 hpf (a–d). A representative region of TUNEL-positive cells is indicated by the brackets (c, d). (E) Whole-mount o-dianisidine staining of hemoglobin in control and TERT-knockdown embryos coinjected with either zp53-MO or zp53-control-MO at 48 hpf. The intensity of the blood flow color over the yolk indicates the hemoglobin concentration (a–d). Wright-Giemsa staining of isolated blood cells from Cont-MO1- and zTERT-MO1-injected embryos in a p53-deficient background at 48 hpf (a'–d'). (F) Whole-mount in situ hybridization of control and TERT-knockdown embryos for *c-myb* and *runx1* expression in *p53^+/+^* and *p53^m/m^* embryos. The expression in the arterial region is indicated by arrowheads. (G) Altered expression levels of the indicated genes in TERT-deficient embryos in a p53-deficient background. Genes involved in the p53 pathway were analyzed by single-embryo RT-PCR. Similar results (data not shown) were obtained from this analysis of a number of individual embryos (more than 10 embryos for each gene). (H) Scoring of the number of circulating blood cells at 72 hpf after co-injection of zTERT-MO1 or Cont-MO1 and either GFP-, hBcl-2-, or E1B-19K-cDNA vectors. Embryos are classified as in (B). (I) Circulating blood cell numbers as a percentage of the control at 72 hpf after co-injection of zTERT-MO1 or Cont-MO1 and either GFP-control, hBcl-2, or E1B-19K expressing vectors. Blood cell numbers were counted in 10 embryos for each group. ^*^ *P*<0.01 (Student t-test).

It has been suggested that p53 is involved in the regulation of apoptosis in hematopoietic progenitors [70], [71]. However, in hematopoietic cells, the evidence of a link between p53-dependent apoptosis and telomerase function has not been well established. To elucidate a possible role of p53 in the changes to the blood cell number and differentiation brought about by TERT deficiency in the zebrafish embryo, we injected zTERT-MOs into p53-mutant (*p53^m/m^*) embryos [72], and into p53 morphants, and compared these with control wild-type embryos. The *p53^m/m^* zebrafish are homozygous for the loss-of-function mutation of M214K: methionine-214 in exon 7 is substituted by lysine. [72]. In both *p53^+/+^* and *p53^m/m^* embryos with a knockdown of zTERT, blood circulation commences at around 24–28 hpf, and the number of blood cells decreases by about 50% at 36–48 hpf (data not shown). By 72 hpf, however, *p53^m/m^* zTERT morphants showed a significant number of blood cells throughout the circulatory system including the heart and caudal vessels, while virtually almost no circulating blood cells are detectable in the *p53^+/+^* background (**Figure 5A–C**). As shown in **Figure 5D**, the apoptotic response of the cells at the CVP in zTERT-deficient embryos is dependent upon the p53 status. At 48 hpf, TUNEL-positive apoptotic cells can be detected in the CVP in zTERT-deficient embryos under normal p53 conditions in the wild-type background as expected. In contrast, TUNEL-positive cells at this region are significantly reduced in number in zTERT- and p53-double deficient embryos obtained via MO injections against the corresponding genes. These same results were also obtained using *p53^m/m^* animals (data not shown).

We next examined the presence of hemoglobin (by o-dianisidine staining) in zTERT- and p53-double deficient animals (**Figure 5E**), as well as in zTERT-deficient *p53^m/m^* fish (data not shown), compared with control animals at 48 hpf. Although the loss of circulating blood cells induced by TERT deficiency was found to be significantly alleviated in both p53 morphants and mutants, o-dianisidine staining revealed still hypochromic blood in these cases, suggesting that the differentiation of erythroblasts remains insufficient or impaired (**Figure 5E, a–d**). Ineffective erythroid cell development showing a blastic phenotype in zTERT-deficient *p53^m/m^* embryos was also evident from Wright-Giemsa staining of isolated blood cells at 48 hpf (**Figure 5E, a'–d'**). The reduction in *c-myb* (n = 52 of 55; 95%) and *runx1* (n = 56 of 58; 97%) expression in zTERT morphants was also unchanged in the p53-deficient genetic background, suggesting that the definitive hematopoietic stem and/or progenitor cell development is not fundamentally restored in the p53 mutants (**Figure 5F**), and p53 morphants (data not shown). Thus, these results indicate that the loss of p53 function can significantly suppress the reduction in blood cell numbers due to zTERT deficiency, but cannot restore the impairment of hematopoietic stem/progenitor cells and their differentiation into mature erythrocytes.

We next monitored the expression of representative genes that are downstream of the p53 pathway in zTERT-deficient animals via single-embryo reverse transcriptase-PCR (RT-PCR). The upregulation of p53 mRNA was evident in the TERT-deficient embryos compared with the controls (**Figure 5G**). In the case of downstream targets of *p53*, *mdm2*, *p21*(/*waf1*/*CIP1*), but not bax, were proportionally upregulated in the zTERT-deficient embryos and this response was suppressed by p53 MO knockdown (**Figure 5G**). This indicated that p53-dependent cell cycle arrest pathways are correspondingly activated in TERT deficient embryos. These single-embryo RT-PCR results are representative of similar analyses that we performed on additional samples (wild-type, n = 12; TERT^MO^-p53^MO^, n = 10; TERT^MO^-p53^cont^, n = 10; TERT^cont^-p53^MO^, n = 10; TERT^cont^-p53^cont^, n = 12; data not shown). Furthermore, in our zTERT morphants, hspa9b/mortalin-2, the deficiency of which has been reported to similarly cause multilineage cytopenia in zebrafish [56], was also found to be slightly upregulated in the absence of significant suppression by p53 knockdown (**Figure 5G**), implicating that an adaptive response exists to sustain blood cell production.

It has been reported that Bcl-2 expression can inhibit the apoptotic response mediated by the p53 pathway in zebrafish as well as in mammals [73], [74], [75]. To test this in our current study, human *bcl-2* cDNA was co-injected with zTERT-MO into zebrafish embryos, and the circulating blood cells in the resulting animals were analyzed. The blood cell numbers were partially but significantly restored, and the circulating blood cells were clearly observed in heart and vessels, at 72 hpf (**Figure 5H, I**) compared with the zTERT-MO controls. The anti-apoptotic adenovirus protein E1B-19K, another member of the Bcl-2 family, was also found to partially rescue the zTERT deficiency-induced blood cell loss. However, neither Bcl-2, E1B-19K overexpression, nor p53 deficiency, could restore blood cell dysplasia, which was evidenced by the presence of immature erythrocytes (erythroblasts) and anemia (data not shown for Bcl-2 and E1B-19K). This suggests that apoptosis is not the primary causative mechanism governing the hematopoietic cell abnormalities caused by the TERT deficiency.

### The restoration of both reduced blood cell number and impaired differentiation in hematopoiesis by the forced expression of both zebrafish and human TERT

As we have already demonstrated, a zTERT knockdown induces both a dramatic reduction in the number of circulating blood cells and an inefficient developmental hematopoiesis, concurrent with the inhibition of telomerase activity in zebrafish in vivo, but in the absence of detectable telomere length alterations. These findings prompted us to investigate whether any activities of TERT that are unrelated to telomerase and telomere functions may be critical for blood cell differentiation and maintenance.

**Figure 6:**
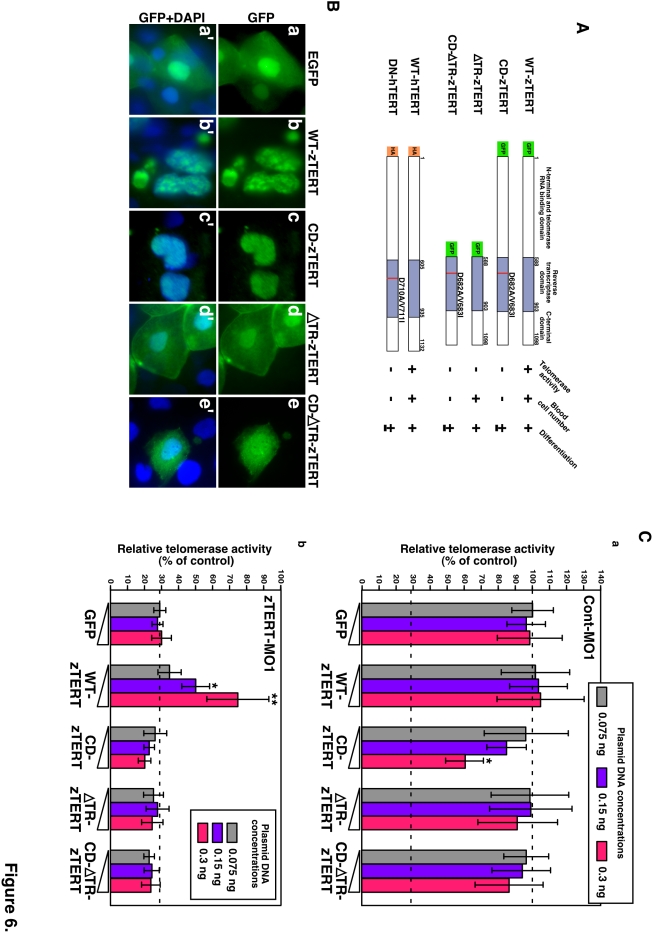
Expression of zebrafish or human TERT in zTERT-deficient embryos. (A) Schematic representations of zebrafish and human wild-type TERT and TERT mutants. All zebrafish TERT (zTERT) fragments were tagged with EGFP at their N-termini. The two amino acid substitutions in the RT domain of TERT (see Materials and Methods) are indicated by the red lines. Deletion mutants of the telomerase RNA-binding domain (ΔTR) of zTERT (zTERT-ΔTR) and its amino-acid substitution mutant ΔTR-CD-zTERT were generated. Human TERT (hTERT) and DN-hTERT were tagged with HA at their N-termini. The presence and absence of telomerase activity, detected by TRAP assay, are indicated as + and −, respectively. Significant recovery of blood cell number is indicated by +, and no significant recovery is denoted by −. Full and partial recovery of blood cell differentiation are indicated by + and ±, respectively. (B) Cellular localization of GFP-wild-type (WT) and -mutant zTERT proteins in the zebrafish embryo. GFP is fused to the N-terminus of each TERT protein. The indicated constructs (see Materials and Methods) were injected into zebrafish embryos, and the subcellular localization of the resulting GFP signals was observed at 48 hpf. (a–e) GFP, and (a'–e') GFP and DAPI. (C) Quantitation of telomerase activity by the expression of zTERT plasmid constructs in zebrafish embryos. Three different concentrations of each zTERT plasmid construct (0.075, 0.15, or 0.3 ng) were co-injected in the indicated combinations with Cont-MO1 (8 ng) (a) or zTERT-MO1 (8 ng) (b), and both the intrinsic and extrinsic telomerase activity was detected by quantitative fluorometric TRAP assay. Each relative telomerase activity value was quantified as a percentage of the activity observed in Cont-MO1-injected embryos expressing a GFP empty vector (GFP) from three independent experiments. ^*^ *P*<0.01, ^**^ *P*<0.001 (Student t-test).

**Figure 7:**
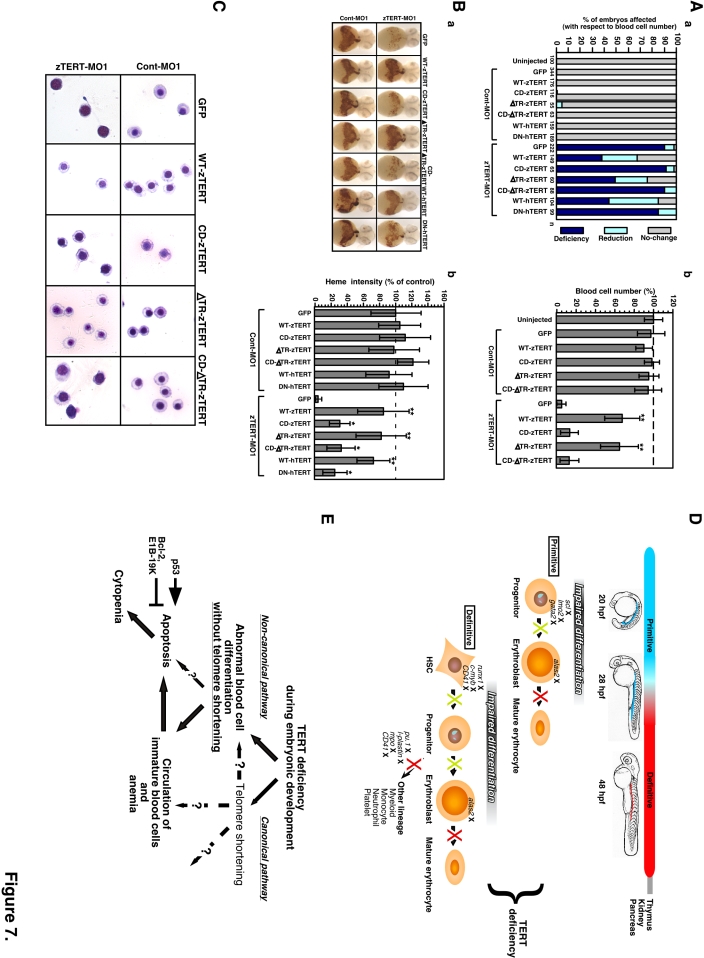
Restoration of ineffective hematopoiesis in TERT-deficient embryos by the expression of zebrafish and human TERT. (A) The blood cell number is rescued in zTERT-MO1-injected embryos (8 ng MOs) at 72 hpf following the injection of the indicated TERT constructs. The percentage of the embryos affected was estimated and assigned into the three categories: No-change, Reduction, or Deficiency, as described above (a). Percentages of control circulating blood cell number in embryos at 72 hpf after the co-injection of several TERT constructs and either Cont-MO1 or zTERT-MO1 at 72 hpf. ^**^ *P*<0.001 (Student t-test) (b). Blood cell numbers were counted in 10 embryos for each group. (B) Rescue of heme appearance, visualized by whole-mount o-dianisidine staining for hemoglobin detection, in TERT-deficient embryos (8 ng MOs) following the injection of several TERT constructs (0.3 ng). Representative samples of whole-mount o-dianisidine staining (a). Percentages of the control heme intensity in embryos at 48 hpf after injection of several TERT constructs with Cont-MO1 or zTERT-MO1 at 48 hpf. ^*^ *P*<0.01, ^**^ *P*<0.001 (Student t-test) (b). Blood cell numbers were counted in 10 embryos for each group. (C) Wright-Giemsa staining of isolated blood cells from Cont-MO1- and TERT-MO1-injected embryos (8 ng) harboring the indicated plasmid constructs (0.3 ng) at 48 hpf. (D) Schematic model of hematopoiesis in TERT-deficient zebrafish embryos. In zebrafish embryos, hematopoiesis occurs in primitive and definitive waves. The first primitive/embryonic wave mainly generates primitive erythrocytes from progenitor cells in the intermediate cell mass (ICM) (represented by the blue color in the 20 and 28 hpf embryo models). The second definitive/adult wave gives rise to hematopoietic stem cells which have the potential to differentiate into all hematopoietic lineages and possess the self-renewal ability to maintain their blood system throughout life. Definitive hematopoietic cells, including stem cells, first arise in the aorta gonad mesonephros (AGM) region (represented by the red color in the 48 hpf embryo model). Definitive hematopoietic stem cells are thought to subsequently colonize the kidney, thymus, and pancreas [53], [88]. Our current model suggests that zTERT deficiency affects both primitive and definitive hematopoiesis in zebrafish, and induces impaired differentiation of the blood cells, including the erythrocyte lineage, prior to maturation. An ‘X’ next to the indicated gene denotes downregulated expression. The yellow ‘X’ in this schematic indicates a somewhat impaired pathway and the red ‘X’ designates a severely impaired pathway. (E) A TERT deficiency in the zebrafish embryo leads to abnormal differentiation and apoptosis, presumably of hematopoietic stem or progenitor cells. This subsequently leads to the circulation of immature blood cells with hypochromic anemia due to a disruption of both primitive and definitive hematopoiesis without telomere shortening. Non-canonical functions of TERT, i.e. authentic telomerase-independent roles, may thus regulate the differentiation of hematopoietic cells, as well as protect these cells from apoptotic cell death during hematopoiesis.

As described above, the TERT protein has a conserved structural organization that is divided into four functional domains; the N-terminal extension domain, the TR-binding domain, the catalytic RT domain, and the C-terminal extension domain (**Figure 6A**) [6]. Each of these domains is required for full activity [6], but the TR-binding and enzymatic RT domains of TERT are particularly critical for telomere maintenance [13]. To investigate the functional significance of these two domains of TERT during hematopoiesis in zebrafish, we generated a series of mutant zTERT constructs (**Figure 6A**) based upon previous studies of hTERT [12], [76], [77], and examined whether these mutant proteins could still rescue blood cell number and differentiation when overexpressed in zTERT-deficient embryos. The positive expression of each construct was confirmed by GFP-tagging and the resulting fluorescence in live animals (**Figure 6B**), and also by western blotting for GFP-tagged zTERT using anti-GFP antibodies and for hemagglutinin (HA)-tagged hTERT using anti-HA antibodies (data not shown). The injection of either wild-type zTERT (WT-zTERT) or hTERT (WT-hTERT) cDNA rescues the blood cell number and differentiation, and reverses both the cytopenia and anemia that accompany maturation failure, in hematopoiesis of zTERT-deficient embryos (**Figure 7A, B** **,** **Figure S9** **,** **Figure S10**).

The specific mutations generating substitutions of the aspartic acid and valine residues at positions 710 and 711 with alanine and isoleucine (D710A and V711I) in the RT motif A of hTERT result in the ablation of authentic telomerase activity and confer dominant-negative properties against the authentic telomerase activity and canonical function of TERT (DN-hTERT) [76], [78]. This metal-binding portion of TERT is highly conserved between zebrafish and humans (**Figure S1**). Therefore, we generated the corresponding mutations in zTERT, D682A and V683I, both in the same RT motif A of zTERT, to generate a catalytic function-defective zTERT mutant (CD-zTERT) (**Figure 6A**). As expected, the expression of CD-zTERT in the zTERT morphants did not restore telomerase activity, confirming that this mutant is catalytically inactive, and also functioned in a dominant-negative manner against authentic telomerase activity (**Figure 6C, a, b**). In contrast, the expression of WT-zTERT in the zTERT-knockdown embryos substantially increased telomerase activity by about two-fold, compared with the control (**Figure 6C, b**). Neither exogenous CD-zTERT nor DN-hTERT caused an obvious rescue of the subsequent cytopenic phenotype for blood cell numbers, but the manifestation evidenced by the heme intensity was slightly restored by both constructs (**Figure 7A, B** **,** **Figure S9** **,** **Figure S10**).

To eliminate the TR-binding ability of zTERT, we created a mutant harboring a deletion of the entire 587 N-terminal amino acids (ΔTR-zTERT), based on the previously identified TR region of hTERT [6], [12] (**Figure 6A**). The ΔTR-zTERT protein did not show any significant telomerase activity (**Figure 6C, b**). However, and more importantly, by expressing this mutant, the cytopenia and anemia phenotypes in the TERT-deficient zebrafish embryos were successfully rescued by restoration of the impaired differentiation of erythrocytes (**Figure 7A, B** **,** **Figure S9** **,** **Figure S10**). A ΔTR-CD-zTERT double mutant, which harbors the TR-binding domain deletion and also contains the D682A/V683I mutations, had no detectable telomerase activity as expected (**Figure 6C** **,** **Figure S11**). The embryos injected with this construct were not significantly protected from endpoint cytopenia, but still slightly recovered from the manifestation of a decreased heme intensity presumably through partial heme synthesis/production in insufficiently differentiated cells (**Figure 7A, B** **,** **Figure S9** **,** **Figure S10**). These findings from both zTERT and hTERT rescue studies indicate that irrespective of its TR-binding ability and authentic telomerase activity, there are non-canonical functions of the RT domain of TERT which may be particularly essential for hematopoietic cell differentiation and survival. In addition, since ΔTR-zTERT no longer specifically localizes in the nucleus (**Figure 6B, d, d'**), but still rescues the hematopoietic phenotype, the activities of ΔTR-zTERT in the cytoplasm may be capable of attenuating any blood cell abnormalities.

Finally, consistent with the results of our rescue experiments described above, cytomorphological abnormalities, such as large nuclei and the basophilic cytoplasms of immature erythrocytes, were found also to be restored by both ΔTR-zTERT and WT-zTERT expression in the morphant embryos (**Figure 7C**). Intriguingly, a moderate level of restoration of these aberrant morphologies in erythrocytes by CD-zTERT and ΔTR-CD-zTERT was also observed (**Figure 7C**). Taken together, these data suggest that a metal-binding-dependent certain novel ‘catalytic’ function of zTERT is critical for the full maintenance of hematopoiesis, but also that other ‘non-catalytic’ function(s) of TERT could be involved in hematopoietic cell differentiation at least in part because mutant TERT (CD-zTERT, ΔTR-CD-zTERT, or DN-hTERT) having a disruption to its metal-binding function still retains a minor ability for this process in heme intensity and cellular morphology. Thus, the canonical TERT function as the authentic/conventional telomerase activity is presumably not essential for hematopoietic cell development observed in our current system. The results of our current study further indicate that an unknown and non-canonical (enzymatic) function(s) associated with the RT motifs of TERT are likely to be required for normal hematopoietic cell development during vertebrate embryogenesis (**Table S1**).

## Discussion

A deficiency in TERT in zebrafish causes ineffective hematopoiesis accompanied by anemia, impaired specification and differentiation, hematopoietic cell apoptosis, and pancytopenia. Moreover, these phenotypes observed in TERT-knockdown zebrafish embryos resemble MDS in humans. Disabled differentiation in hematopoietic stem and/or progenitor cells may underlie both the apoptotic response and maturation defects which occur in both primitive and definitive hematopoiesis, although further studies will be required to definitively elucidate this relationship. The disruption of the hematopoietic cell lineages caused by the zTERT deficiency in our current study was observed in the absence of telomere length alterations, although both the apoptotic and cytopenic phenotypes in these mutants were still dependent, at least in part, upon p53 and Bcl-2 functions. Intriguingly, the overexpression of the RT domain of zTERT (without the TR-binding domain) as well as full-length zTERT rescues the TERT-deficient animals from pancytopenia, with a concurrent restoration of differentiation. Taken together, our data suggest that zTERT is involved in both the differentiation and survival of hematopoietic cells independently of its canonical role in telomere maintenance.

### A crucial role of TERT in hematopoietic cell differentiation and survival in vertebrates

It has been reported previously that the zebrafish Hspa9b/Mortalin-2 mutant shows a developmental blood defect that closely recapitulates the ineffective hematopoiesis seen in MDS patients [56]. In our present study, we demonstrate that zTERT-deficient embryos show a reduction in their red blood cell numbers and harbor myeloid cells with abnormal differentiation, indicating the occurrence of pancytopenia. These abnormalities have also been reported in mutant Hspa9b/Mortalin-2 embryos. In our current TERT morphants, in addition to reductions in the levels of *scl^+^* and *lmo2^+^* primitive hematopoietic progenitors, *c-myb^+^* and *runx1^+^* definitive hematopoietic stem cells as well as *CD41^+^* hematopoietic precursor cells were also found to be significantly decreased (**Figure 3**, **Figure S7**), suggesting that hematopoietic stem cell populations are also impaired by the loss of TERT. Since MDS is a clonal hematopoietic stem cell disease characterized by ineffective hematopoiesis and peripheral cytopenias, TERT deficiency in zebrafish possibly recapitulates this disorder. During zebrafish hematopoiesis, definitive progenitors can be observed in the posterior ICM and in the dorsal aorta as early as 24 hpf [79], [80], [81]. However, since it is unknown whether zebrafish hematopoietic stem cells continue to arise de novo, we cannot rule out the possibility that a failure of later arising stem cell populations to develop may lead to the actual blood cell reduction which we observe by 72 hpf.

Although there is accumulating evidence that apoptosis plays a crucial role in hematopoietic stem and progenitor cell development, the precise mechanisms and roles of TERT in apoptotic signaling pathways have not so far been elucidated. Recent reports strongly suggest that p53, Puma, Slug, and Bcl-2 family members, including Mcl-1, play crucial roles in the agonistic versus antagonistic regulation of apoptosis in hematopoietic stem and/or progenitor cells [70]. In our current zTERT morphants, decreases in the circulating blood cells were restored by either a p53 knockdown by MOs or a p53 mutant background. The overexpression of Bcl-2 or E1B-19K was also found to counteract the reduction in the number of blood cells due to cytopenia. Interestingly, caspase-3 activation was only detectable during primitive hematopoiesis in these TERT morphants from 19–24 hpf, and not at later stages (data not shown). Furthermore, inhibition of this early caspase activation decreases the levels of TERT knockdown-induced apoptosis in the ICM primitive wave (∼28 hpf), whereas a later apoptotic response observed in the CVP (48–72 hpf) is unaffected by this caspase suppression (data not shown). Notably also, caspase inhibition does not appear to be effective in inhibiting the cytopenic phenotype of the zTERT morphants. Thus, the later apoptosis mediated by p53 in the CVP of the zTERT knockdown embryos may be independent of early caspase activation, but is presumably linked with the induction of cytopenia that is evident by 72 hpf. Further analyses will be required to determine the underlying mechanisms connecting the early and late phases of apoptosis during the transition stages from primitive to definitive hematopoiesis.

Importantly, none of the interventions (p53 deficiency, Bcl-2, and E1B-19K) used in our present experiments that inhibit apoptosis were capable of restoring the insufficient differentiation and maturation of erythrocytes in the zTERT morphants (**Figure 5E**, and data not shown), suggesting that TERT is an essential component of certain processes underlying hematopoietic cell differentiation, regardless of the survival pathways that may be initiated.

### Differing functions of TERT in hematopoiesis among vertebrate species

The well documented and conventional function of TERT is its role as the catalytic component of telomerase which is indispensable for the maintenance of telomeres, and thus genome integrity [7]. However, there are several fundamental differences in both the telomere and telomerase biology of different vertebrates. In lower vertebrates in particular, such as zebrafish, telomerase is constitutively active in multiple organs and may support continuous growth of the animal throughout its lifespan [20], [21], [22], [23]. In contrast, higher vertebrates have more tightly regulated telomerase activity and most normal somatic cells usually do not have active telomerase, except for stem/progenitor cells. Cells that become cancerous also have active telomerase in most cases.

Moreover, in terms of telomere length, mouse telomeres (40–60 kb) (particularly in inbred mice) are much longer than human telomeres (10–15 kb), although long telomeres do not appear to be necessary for survival in the mouse [20], [29], [82], [83], [84]. Zebrafish telomere lengths (15–20 kb) are relatively similar to those in humans, based upon the results of our current study. We would contend therefore that the roles of telomeres and telomerase during hematopoiesis cannot be inferred simply from a single animal model but needs to be studied comparatively in several vertebrate species, as well as directly in human cells. This also means that the role of not only telomerase, but also TERT in hematopoietic cells is still incompletely understood. In TR-knockout mice, as well as in TERT-knockout mice, it has been suggested that telomeres show moderate shortening over several generations [43], and subsequently suffer from impaired cell proliferative capability in highly proliferative tissues [17]. On the other hand, even a partial telomerase deficiency in humans brought about by mutations in either TR or TERT has been associated with early or late onset bone marrow failure, as seen in patients with dyskeratosis congenita and aplastic anemia [48], [49], [85]. Bone marrow failure or spontaneous and sporadic anemia has been reported in TR-knockout mice [50], but not in TERT-knockout mice, although further characterizations of TERT are required in the mouse. In our present zebrafish studies, however, a pancytopenia phenotype was observed in TERT-knockdown embryos following MO injections, which was rescued by following injection of a zTERT mutant (ΔTR-zTERT) lacking the TR domain, suggesting that the cytopenia is not due to direct inhibition of authentic telomerase activity itself. Given our findings of lengthening-independent and TR-binding domain-unrelated TERT function(s), hematopoiesis in zebrafish might be more susceptible to the disruption of a non-canonical function of TERT in comparison with the mouse.

### Novel non-canonical functions of zebrafish TERT in hematopoiesis

In previous studies, hematopoietic stem cells from TERT-deficient as well as TR-deficient mice showed reduced repopulating abilities by serial transplantation assay [86], demonstrating that both TERT and TR are critical for normal stem cell function via sustained telomere length maintenance. It has also been reported that TERT overexpression enhances the self-renewal ability of embryonic stem cells, promotes their resistance to apoptosis, and increases their proliferation and ultimate differentiation into hematopoietic lineages [87]. However it is presently unclear to what extent telomere length maintenance via TERT is essential for stem cell activation. Recent studies of TERT overexpression in mice clearly show that telomere length-independent novel function(s) are responsible for hair follicle stem cell mobilization and proliferation, regardless of telomere synthesis [27], [29]. However, one previous report has rather surprisingly showed that TR was still required for this function of TERT [27], whereas another has demonstrated that a TR-unrelated function is essential for this alternative role of TERT [29]. Most recently, Choi et al. further reported that a catalytically inactive mutant of TERT (TERT^ci^) retains the full activity of wild-type TERT in hair follicle stem cell activation and keratinocyte proliferation [40]. Our current finding that the ΔTR-zTERT rescues the effects of zTERT depletion is consistent with a TR-independent functional role (**Table S1**). The evidence that CD-zTERT significantly loses its function in hematopoiesis further suggests that TR-independent unknown (catalytic) activities of TERT may be involved in hematopoietic cell differentiation and survival. Therefore, there are at least three types of telomere metabolism-independent non-canonical functions of TERT; the first of these still requires both TERT and TR [27], the second does not requires the catalytic activity of TERT (i.e., TERT^ci^) and TR [29], [40], and the third that we have elucidated herein does not require the authentic (conventional) catalytic activity of TERT but requires TR-independent (catalytic) activities/functions of TERT.

When and how does TERT function non-canonically? It is possible that if the telomeres are longer than a certain minimal length, TERT may perform non-canonical functions at intracellular locations other than telomeres. Under these circumstances, TERT may be able to alternatively function in cellular proliferation and mobilization, or may be required for cellular differentiation and survival independent of telomere metabolisms. Our present findings support this notion. Hence, zTERT-deficient embryos exhibit severe anemia and cytopenia that presumably results from disruption of the primitive and definitive waves of hematopoiesis in the absence of alterations in telomere length. Furthermore, a zTERT deficiency likely causes defects in hematopoietic stem and/or progenitor cells. TERT might affect the self-renewal of stem cells as well as normal specification and differentiation of stem/progenitor cells during zebrafish hematopoiesis when these cells have telomeres of sufficient length and integrity. Although an extremely minor number of critically short telomeres may still affect the maintenance of hematopoietic lineages, it should be noted that we did not detect any significant differences in the lengths in critically short telomeres or the average telomere lengths in zTERT-deficient embryos. No pattern of short telomeres at the same chromosome ends was evident either in the zTERT morphants i.e. specific chromosome ends with short telomeres were essentially randomly distributed in both the control and zTERT-MO1- or MO2-injected embryos (**Figure S5**).

On the other hand, our current results do not exclude the possibility that the telomere lengths themselves are also critical for the maintenance of hematopoietic cell lineages. In fact, it has been reported that wild-type mice derived from the late generation of *mTR^+/−^* heterozygous parents which had short telomeres display hematopoietic phenotypes resembling aplastic anemia and dyskeratosis congenita [50]. However, our data strongly suggest that non-canonical TERT functions exist that are separate from its role in telomere maintenance via telomerase and regulate hematopoietic cell differentiation and survival, presumably without affecting telomere length.

In zebrafish embryos, as shown in **Figure 7D**, hematopoiesis occurs in primitive and definitive waves. The first primitive/embryonic wave mainly generates primitive erythrocytes from progenitor cells in the ICM (represented by blue color in the 20 and 28 hpf embryo models). The second definitive/adult wave gives rise to hematopoietic stem cells which have the potential to differentiate into all hematopoietic lineages and possess the self-renewal ability to maintain the blood system throughout life. Definitive hematopoietic cells, including stem cells, first arise in the AGM region (represented by red color in the 48 hpf embryo model). Definitive hematopoietic stem cells are thought to subsequently colonize the kidney, thymus, and pancreas [53], [88]. Our current model suggests that zTERT deficiency affects both primitive and definitive hematopoiesis in zebrafish, and induces impaired differentiation of the blood cells, including the erythrocyte lineage, prior to maturation. Moreover, TERT deficiency leads to the abnormal differentiation and apoptosis presumably of hematopoietic stem and/or progenitor cells, subsequently leading to the circulation of immature blood cells with anemia, by disturbing normal hematopoiesis without obvious telomere shortening. Non-canonical functions of TERT other than telomerase may thus regulate the differentiation of hematopoietic cells, as well as protect them from apoptotic cell death during developmental hematopoiesis (**Figure 7E**).

An important question that arises from our current data is the nature of the mechanism that underlies this non-canonical function of zTERT. In many other species, the RT domain of TERT has not been demonstrated to contribute to TR binding, although this has not been directly confirmed in zebrafish [31]. Because TERT mutants bearing deletions in their TR-binding domain functionally rescue both the circulating blood cells and defective differentiation of hematopoietic cells in zTERT morphants, an unknown activity of the RT domain of TERT, other than telomerase activity, may be crucial for hematopoiesis. Our data thus encourage further studies to address three important questions: (1) the nature of the mechanisms by which TERT can regulate hematopoietic cell differentiation and survival without telomere regulation; (2) the intracellular locations and regions where these non-canonical functions operate within the cell; and (3) the precise target of the non-canonical function(s) of the RT domain of TERT.

In the phylogenetic tree of TERT and retroelements rooted with RNA-dependent RNA polymerases, the RT motifs in the RT family are universally conserved [including multicopy single-stranded DNA, group II introns, LTR or non-LTR (Long Terminal Repeat) retrotransposons, and viral RT] [10], [89]. Structural and functional analyses of viral RT and TERT have revealed striking degrees of conservation of important residues within the RT motifs involved in nucleotide binding, rNTP/dNTP discrimination, and nucleotide addition processivity between viral RT and TERT [10]. Unlike all other RT family members, only TERT carries an N-terminal extension as its RNA-binding domain. Based on these facts, it is tempting to speculate that the high-affinity RNA-binding domain of TERT defines (or confines) telomere-interacting specificity, but the RT domain itself may function in a conventional way in certain genomic DNA structures. Alternatively, it has been recently demonstrated that both yeast and human TERT can function as a template- and RNA-independent terminal transferase (TT) for DNA synthesis in the presence of Mn^2+^ [90]. Conceivably, this TT activity might underlie one of the functional roles of the TR-binding domain-deleted zTERT mutant. Although the intrinsic ability of TERT to act as an intracellular TT in vertebrates still needs to be demonstrated in vivo, a variety of DNA and RNA polymerases including RTs are well known to mediate template-independent nucleotide addition under certain conditions [90]. Irrespective of the telomere ends, such a template- and RNA-independent TT activity of TERT may be capable of functioning in the repair of broken terminal ends.

It is also possible that RT motifs themselves might interact with some regulatory non-protein-coding RNAs (regulatory RNAs), such as microRNAs, which have been shown to be involved in modulation of certain gene expressions at both the transcriptional and post-transcriptional level. Such regulatory RNAs participate in many mechanisms that regulate chromatin modification and transcription factor activity, and influence mRNA stability, processing, and translation, all of which are key factors in multiple aspects of differentiation and development, including hematopoiesis [91], [92], [93]. Indeed, it has already been shown that TERT can influence the expression of a number of other genes such as *p53*, *p21*, *pRb*, *cyclin D1*, *epidermal growth factor receptor*, *transforming growth factor-β*, and *DNA methyltransferase* [40], [94], [95], [96], [97], [98], [99], [100], and is presumably therefore involved in the regulation of several genes. In our current analysis of zebrafish embryogenesis, we could also observe a differential gene expression profile induced by zTERT knockdown with regard to hematopoietic cell differentiation for the *scl*, *lmo2*, *gata-2*, *runx1*, *c-myb*, *alas2*, *pu.1*, *l-plastin*, *mpo*, and *CD41* genes (a downregulation of their expression) (**Figure 7E**), in addition to the induction of *p53*, *p21*, and *mdm2*. These data indicate that a zTERT deficiency may induce a defect in hematopoiesis not through telomere dysfunction, but through a potent and specific effect on the gene expression of several key regulators during early development in zebrafish.

Clearly, further studies will be necessary to more precisely determine the physiological non-canonical functions of TERT during hematopoietic cell differentiation and survival. Our current data demonstrate however that the zebrafish model may provide powerful new information regarding the hematopoietic program that is physiologically relevant to both vertebrate biology, and to telomerase- and telomere-related clinical disorders of human blood formation.

## Materials and Methods

### Zebrafish maintenance

Zebrafish (*Danio rerio*) were raised and maintained under standard laboratory conditions at 28.5°C in a 14 h light/10 h dark cycle [101], [102], [103]. Embryos were staged by hours post fertilization (hpf) at 28.5°C and by morphological criteria [104]. Previously established p53-mutant zebrafish (tp53^M214K^) were also used in the experiments [72].

### Microinjection of morpholino oligonucleotides and cDNAs

Morpholino antisense oligonucleotides (MO) (Gene Tools, LLC) were used for knockdown of both the *zTERT* and *zp53* genes. The stock solution was diluted to 250 or 500 µM. The sequence of zTERT MO1 (zTERT-MO1) is 5′-CTGTCGAGTACTGTCCAGACATCTG-3′, which is at the 5′-translation start site. The complementary sequence of the putative zTERT ATG start site is underlined. The sequence of the inverse zTERT control MO1 is 5′-GTCTACAGACCTGTCATGAGCTGTC-3′ (data not shown), and the sequence of the 5-mispair zTERT control MO1 (Cont-MO1) is 5′-CTCTCCAGTACTCTCCACACATGTG-3′. The splice-block morpholino antisense oligonucleotide of zTERT (zTERT-MO2) was used to confirm that the phenotypic effects of zTERT-MO1 were TERT-specific. The sequence of zTERT-MO2 is 5′-CACTCACACATTGAAGAGCTTCACC-3′, which is at the 3′ end of exon 5 and its intron splice junction. The sequence of the 5-mispair control MO (Cont-MO2) is 5′-CAGTCACAGATTCAACAGCTTGACC-3′. Both the zp53 and 4-mispair zp53 control MOs have been described previously [105].

GFP-tagged wild-type zTERT and mutant zTERT constructs were used in the rescue experiments. One point six to eight ng of the TERT-MO or Cont-MO and 75–300 pg of the TERT or EGFP (enhanced GFP) plasmids were injected into single-cell stage embryos at the yolk and cytoplasm interface.

### Plasmids

A 2,287 base-pair fragment of full-length zTERT cDNA (GenBank accession number; submitted) was isolated by RT-PCR (see supplemental Text S1), and cloned into the pEGFP-C1 vector (Clontech). pCI-neo-hTERT-HA (WT-hTERT) and pCI-neo-DN-hTERT-HA (DN-hTERT) were kindly provided by William Hahn [76]. The mutated region of the DN-hTERT is conserved in zebrafish TERT (**Figure S1**), and we therefore generated a DN zTERT accordingly (catalytically inactive zTERT; CD-zTERT) by substituting the aspartic acid and valine residues at positions 682 and 683 with alanine and isoleucine residues, respectively. This was achieved by site-directed mutagenesis of the WT-zTERT vector using the oligonucleotides, 5′-GCTCTACTTCGTCAAGGTCGCGATCAGCGGAGCGTATGACAG–3′ and 5′-CTGTCATACGCTCCGCTGATCGCGACCTTGACGAAGTAGAGC–3′. The mutant construct was then confirmed by sequencing. Deletion vectors were generated by PCR using WT-zTERT and CD-zTERT as templates. zTERT 1732-3388 (EcoRI-SalI) was amplified by PCR using the primers 5′-ACGCGAATTCGAAGGGCCAGTGGAGGCCCCTGTCTCCATC-3′ and 5′-GAGAGTCGACGGGCAGTGCAGATGTGTTTAGTCAGC-3′, and cloned into the pEGFP-C1 vector.

### Monitoring of zTERT knockdown in vivo using a 5′ fragment of zTERT-EGFP

A 5′ fragment containing the translational initiation site of zTERT cDNA (-3-123) was amplified by PCR from a wild-type zTERT expression vector (WT-zTERT vector). The amplified DNA fragments were subsequently purified by excision from an agarose gel. zTERT -3-123 (EcoRI-SalI) was amplified using the primers 5′-GAGAGAATTCCACAGATGTCTGGACAGTACTCGA-3′ and 5′-AGAGGTCGACTGTCGGCCGTCAGGGAATTGCAGT-3′, and was then cloned into pEGFP-N1 vector (pEGFP-5′zTERT) (**Figure S4, a**). 1–2 nl of 100 ng/µl pEGFP-5′zTERT or empty vector (pEGFP-N1) was coinjected with zTERT-MO1 or Cont-MO1 into the yolk sac of embryos at the single-cell stage, and observed at 24 hpf using a Zeiss Axioskop microscope. At least 25 embryos in each injection group were captured and quantified by determining the pixel intensity of the GFP signals using Photoshop software.

### RT-PCR analysis of zTERT splicing block by zTERT-MO2

For RT-PCR detection of the zTERT splicing block by MO2, forward and reverse primers to amplify a fragment between exon 4 and 8 of zTERT were used (5′- AGTGACATCCCGCATCCGCTTTAT-3′ and 5′- AGGGCTTTCTCCATGTGTCCGTAAC-3′, respectively). Control β-actin primers were also used (5′-CCCAGACATCAGGGAGTGAT-3′, and 5′-CACCGATCCAGACGGAGTAT-3′, respectively) as previously described [73].

### Assay for telomerase activity (TRAP assay)

For telomerase activity measurements in zebrafish embryo and cell extracts, the TRAP (Telomere Repeat Amplification Protocol) assay was performed using three different types of detection methods.

The TRAPeze® telomerase detection kit (Chemicon), was used for the electrophoretic gel analysis according to the manufacturer's instructions. The PCR products were separated on 10% polyacrylamide gels and visualized with SYBR® Green (Molecular Probes).

The TRAPeze® XL kit (Chemicon) was used for a PCR-based quantitative fluorescence assay for telomerase activity detections. The TRAPeze® XL kit uses Amplifluor® fluorescence energy transfer labeled primers to obtain semi-quantitative measurements, which enables homogeneous signal amplification and quantification directly in the unopened PCR vessel. For measurement of samples, we used µQuant Microplate Spectrophotometer (BioTek Instruments) with the excitation/emission parameters for fluorescein (495 nm/516 nm) and sulforhodamine (600 nm/620 nm)

The TeloTAGGG Telomerase PCR ELISA^PLUS^ (Roche Applied Science) was used for a PCR-based quantitative ELISA assay for telomerase activity detections according to the manufacturer's instructions. Using µQuant Microplate Spectrophotometer (BioTek Instruments), samples were measured by the absorbance at 450 nm with a reference wave length of 690 nm within 30 min after adding the stop reagent.

### Whole-mount measurement of zebrafish telomere lengths in interphase nuclei by fluorescence in situ hybridization

Individual telomere lengths were analyzed by quantitative fluorescence in situ hybridization (Q-FISH). Q-FISH was performed using a Cy3-conjugated OO-(CCCTAA)_3_ PNA oligonucleotide (Cy3-telomere PNA probe) (Applied Biosystems) as described [106]. Metaphase spreads were washed in PBS for 15 min and fixed in 4% paraformaldehyde/PBS for 2 min. After dehydration, a Cy3-telomere PNA probe (10 nM) was added, and the samples were incubated at 85°C for 5 min for denaturation, and then in hybridization solution (20 mM Tris-HCl (pH 7.2), 70% formamide, and 1% BSA) overnight at room temperature in the dark. After incubation, the embryos were washed three times in 70% formamide, 10 mM Tris HCl (pH 7.2) for 15 min, and twice again in 10 mM Tris-HCl (pH 7.2), 150 mM NaCl, 0.05% Tween 20), and then counterstained with 0.5 µg/ml 4′,6-diamidino-2-phenylindole (DAPI). Images were acquired by using a Zeiss Axioskop microscope. Telomere profiles were analyzed using the TFL-TELO software provided by Peter Lansdorp [107].

### Q-FISH of zebrafish telomeres in embryonic metaphase nuclei

Chromosome preparations were generated using a modification of the method described in The Zebrafish Book (University of Oregon). Briefly, 8 hpf zebrafish embryos were treated with 1 mg/ml colchicine (Sigma) at 28.5°C for 14–16 h and then harvested at 22–24 hpf. Embryos were washed with PBS containing 10% fetal bovine serum (FBS), mashed and filtered through a 100 µm nylon filter to be suspended cells. The cells were then resuspended in 1.1% sodium citrate and 4 mg/ml colchicine for 25°C for 25 min, and fixed in cold methanol∶glacial acetic acid (3∶1). The resulting suspensions were dropped onto wet pre-warmed microscope slides (37°C) along with a chromosome suspension from the human VA-13+hTERC+hTERT cell line provided by Jerry Shay [108]. FISH with a PNA-telomere probe was then performed as described above.

The abnormal karyotype of the VA-13+hTERC+hTERT cell line is characterized by the presence of a cytogenetically identifiable, duplicated marker chromosome that bears microscopically visible interstitial telomeric repeats. The simultaneous hybridization of human and zebrafish metaphases on the same slide facilitated the use of specific interstitial human telomeres as internal controls. Per condition, we assessed 468 telomeres corresponding to 468 chromatids from 15-well spread metaphase images that had no saturated signals. We measured each of the chromatids in a preselected quarter of a metaphase plate (i.e. the upper part of the metaphase between hours 12 and 3). All chromatid telomeric signals of a particular area were measured (whether visible or not). Sister chromatids were preferentially measured. The values (A-values) of Fluorescence Intensity (FI), as measured using the MetaSystems ISIS software and the TFL-TELO software in the unprocessed image, were calculated taking into account the overall measured area (MA) that was usually similar for both sister chromatids (A-value = FI/MA). When only one of the two sister chromatids had a detectable telomeric signal, we used the MA of the sister chromatid with no telomeric signal and measured the tip of the chromosome, utilizing its observed morphology following DAPI staining. The mean measured area/metaphase (mMA) was used when no telomeric signal at either end of a particular chromosome could be observed. Hence, the lowest and highest detectable values were equally and stochastically represented by our measurements.

The Metasystems Isis software automatically adjusts for the exposure time so most of the fluorescent spots per metaphase were detectable when the number of saturated spots was kept as low as possible. Our imaging system (based on a Zeiss Imager Z1 and a Metasystems II videocamera) also showed great linearity between the exposure time and measurable fluorescence intensity when tested using fluorescent microbeads (Molecular Probes) prior to undertaking the measurements in zebrafish. To correct for time exposure variations, we took into consideration the exposure time (t) which was unique for each metaphase so that the corrected B-value = A-value/t. We then normalized the B-value, using internal controls from four identical marker chromosomes with interstitial telomeres (m1va13), which were co-hybridized on the same microscope slide with the zebrafish specimen. We used inverted DAPI banding and PNA-telomere FISH patterns to identify duplicate copies of m1va13. We then measured, as indicated above, the specific interstitial telomeres of four chromatids per metaphase. The mean internal control value (IC-value) for 8 counts per slide, was measured as follows: Relative Telomeric Fluorescence Intensity (RTFI) = B-value×IC-value.

### Assessment of telomere lengths by terminal restriction fragment Southern blotting

Genomic DNA was isolated from three embryos of each genotype which were suspended in 100 µl of genomic DNA extraction buffer [125 mM NaCl, 50 mM EDTA, 1% SDS, 10 mM Tris-HCl (pH 7.5), 0.4 mg/ml Proteinase K] and agitated at 50°C overnight. Genomic DNA was then purified by phenol-chloroform and ethanol purification and 1 µg was digested with *HinfI* and *RsaI* enzymes (Roche applied science), and resolved on 0.8% agarose gels in 1× TAE. The gels were then denatured, blotted onto charged nylon membranes (Amersham Biosciences), and hybridized with a digoxigenin (DIG)-labeled telomeric DNA probe (5′-CCCTAA-3′)_4_ in DIG Easy Hyb (Roche Applied Science). The membranes were washed twice in stringent wash buffer 1 (2× SSC and 0.1% SDS) for 15 min each, and twice again in stringent wash buffer 2 (0.2× SSC and 0.1% SDS) at 50°C also for 15 min each. Telomere fragments were detected and visualized using an anti-DIG-AP antibody (Roche Applied Science) and CDP-Star® reagent (Roche Applied Science).

### Whole-mount detection of apoptosis

Zebrafish embryos from 19 to 72 hpf were fixed overnight in 4% paraformaldehyde at 4°C and stored in 100% methanol at −20°C. Samples were then incubated in acetone at −20°C for 20 min, in 0.5% Triton X-100 and 0.1% sodium citrate in PBS for 15 min, and then treated with 5 to 50 µg/ml proteinase K (Invitrogen) for 5 to 25 min, depending on the embryo stages. After fixation, the embryos were subjected to a TUNEL assay via the ApopTag® Red in situ apoptosis detection kit (Chemicon), according to the manufacturer's instructions.

### Detection of apoptosis for circulating blood cells

To detect circulating blood cells, *gata-1^GFP^* transgenic fish were used. The *gata-1^GFP^* fish embryos were fixed in 4% paraformaldehyde/PBS at 4°C overnight, and cryostat sections (10 µm) were then generated and stained using an anti-GFP polyclonal antibody (Wako) (1∶500) and FITC-labeled anti-rabbit antibody (1∶500). After staining, apoptotic cells were further detected by the TUNEL assay (Chemicon), and sample sections were then counterstained by 0.5 µg/ml DAPI.

### Measurement of circulating blood cells

For the quantification of circulating blood cell numbers in caudal blood vessels, the numbers of blood cells in 0.5 mm of the dorsal aorta were counted for at least 10 embryos from 28 to 72 hpf. In order to obtain a phenotypic score of the blood cell number, each embryo was then scored according to the number of circulating blood cells. The embryo groups were divided into three classes based on their flowing blood cell numbers: i) indistinguishable from the control (>90%; No-change), ii) reduced in number (10–90%; Reduction), or iii) severely deficient or almost no flowing cells (<10%; Deficiency).

### Whole-mount heme staining, histologic staining, and Wright-Giemsa staining

Heme was detected by whole-mount o-dianisidine staining as described previously [109]. Briefly, embryos under anesthesia were stained in o-dianisidine solution [(0.6 mg/ml o-dianisidine, 0.01 M sodium acetate (pH 4.5), 0.65% hydrogen peroxide, 40% ethanol)] for 15 min in the dark. For histological observations, plastic sections (Technovit 8100, Heraeus Kulzer) were made according to the manufacturer's protocol, and stained with hematoxylin-eosin. Embryonic blood was isolated by cardiac puncture, and smeared onto glass slides and Wright-Giemsa staining was performed as previously described [110].

### Whole-mount in situ hybridization of embryos

For in situ hybridization, DIG-labeled antisense RNA probes for zTERT cDNA and hematopoietic marker genes were synthesized using a DIG RNA labeling kit (Roche Applied Science). For the controls, sense RNA-labeled probes were synthesized. In situ hybridization of whole-mount zebrafish embryos was then performed as described [111]. Briefly, prior to hybridization, the embryos were fixed in 4% paraformaldehyde with PBS at 4°C for 15 h (overnight). Before staining, embryos were rehydrated for 10 min in PBS-T containing 75, 50, and 25% methanol and washed for 5 min in PBS-T. Hybridization with the labeled RNA probes was carried out at 68°C overnight in hybridization solution (50% formamide, 5× SSC, 0.5% yeast RNA, 5% heparin, 0.1% Tween-20). Embryos were then incubated for 30 min at room temperature with an anti-DIG antibody (Roche Applied Science) (1∶2,000). Colorimetric detection was carried out using BM Purple substrate (Roche Applied Science). The reaction was stopped by washing in PBS, and then the embryos were examined by a light-microscopy (SMZ-U, Nikon).

### Preparation of embryonic cells and FACS analysis

Embryonic cells from *gata-1^GFP^* transgenic embryos were collected as previously described [69]. Approximately 300 dechorionated embryos were collected in tubes containing 500 µl of a 1× Trypsin/EDTA solution, and were digested by pipetting for 5 to 10 min. Digested cell suspensions were then added to 1 ml of PBS containing 5% FBS and passed through a 70 mm nylon mesh filter. Cells were then collected by centrifugation at 400 rpm for 5 min, and resuspended in PBS/5% FBS (1 ml). After centrifugation, cells were resuspended PBS/1% FBS and 50 µg/ml propidium iodide. FACS sorting was performed with a MoFlo flow cytometer (Dako Cytomation). After 2-time sorting, GFP-positive cells were smeared on glass slides and processed for Q-FISH analysis.

### RNA isolation and RT-PCR analysis

Total RNA was extracted from 48 hpf zebrafish embryos using Trizol® reagent according to the manufacturer's protocol (Invitrogen). Double-stranded cDNA was synthesized using M-MLV reverse transcriptase (Promega), and PCR was performed using ExTaq (Takara). For RT-PCR detection, the forward and reverse primers used for zTERT were 5′-AAACGCTTCTGCACCAAAGCTGAG-3′ and 5′-AGTGGTCAGGAACTCTGTGGCTTT-3′, respectively. The primers for *p53*, *p21*, *mdm2* and *β-actin* have been previously described [73]. Other primers that were designed for this study included: *EF1α*; forward, 5′-ACCACCGGCCATCTGATCTACAAA-3′ and reverse, 5′-ACGGATGTCCTTGACAGACACGTT-3′, and *Hspa9b*; forward, 5′-ACATGAAGCTCACACGGTCTCAGT-3′ and reverse, 5′- AGCCACCAGATGACTGGATGACAA-3′. Amplification was performed at an initial denaturation step at 94°C for 5 min, followed by 25 cycles of 30 sec at 94°C; 30 sec at 60°C; and 60 sec at 72°C, and a final extension step of 10 min at 72°C. PCR products were separated on 1.5% agarose, ethidium bromide-stained gels, which were then imaged using a Multi Image Light Cabinet (Alpha Innotech Corporation).

## Supporting Information

Text S1 (0.10 MB DOC)

Table S1 (0.05 MB DOC)

Figure S1 (1.05 MB PDF)

Figure S2 (0.31 MB TIF)

Figure S3 (0.52 MB TIF)

Figure S4 (0.15 MB TIF)

Figure S5 (2.38 MB TIF)

Figure S6 (0.37 MB TIF)

Figure S7 (0.63 MB TIF)

Figure S8 (0.41 MB TIF)

Figure S9 (0.44 MB TIF)

Figure S10 (0.69 MB TIF)

Figure S11 (0.56 MB TIF)
