# Correction: A non-canonical function of zebrafish telomerase reverse transcriptase is required for developmental hematopoiesis

**DOI:** 10.1371/journal.pone.0350446

**Published:** 2026-05-29

**Authors:** Shintaro Imamura, Junzo Uchiyama, Eriko Koshimizu, Jun-ichi Hanai, Christina Raftopoulou, Ryan D. Murphey, Peter E. Bayliss, Yoichi Imai, Caroline Erter Burns, Kenkichi Masutomi, Sarantis Gagos, Leonard I. Zon, Thomas M. Roberts, Shuji Kishi

After this article [1] was published, concerns were raised regarding Figs 2 and 5. Specifically:

- In Fig 2B, panels e and h appear similar to each other.
- In Fig 5F, both of the *runx1*/zTERT-MO1 panels appear similar to each other.

Co-first author SI stated that panel h of Fig 2B and the *runx1 p53*^*m/m*^ zTERT-MO1 panel of Fig 5F are incorrect and provided corrected versions of Figs 2 and 5. Additionally, the corrected Fig 2 updates all panels of Fig 2B with enlarged heart regions of the embryos. Co-first author SI further stated that the duplication of these panels occurred during figure preparation, and that the associated quantitative data is unaffected.

In addition, the IACUC approval numbers for animal study and methods of anesthesia were omitted from the “Zebrafish maintenance” section of the Materials and Methods. Co**-**first author SI stated that all animal procedures were approved by the IACUC committee at Schepens Eye Research Institute under codes S-150–0909 and S-186–1210, and that zebrafish embryos were anesthetized following the protocol outlined in [2].

The original images underlying panels e-h of Fig 2B, as well as the quantitative data underlying Figs 2Aa and 2C, have been included with this notice as S1 File. The original images underlying the *runx1* zTERT-MO1 panels of Fig 5F, as well as the quantitative data underlying Figs 5B-C and 5H are included as S2 File.

**Fig 2:**
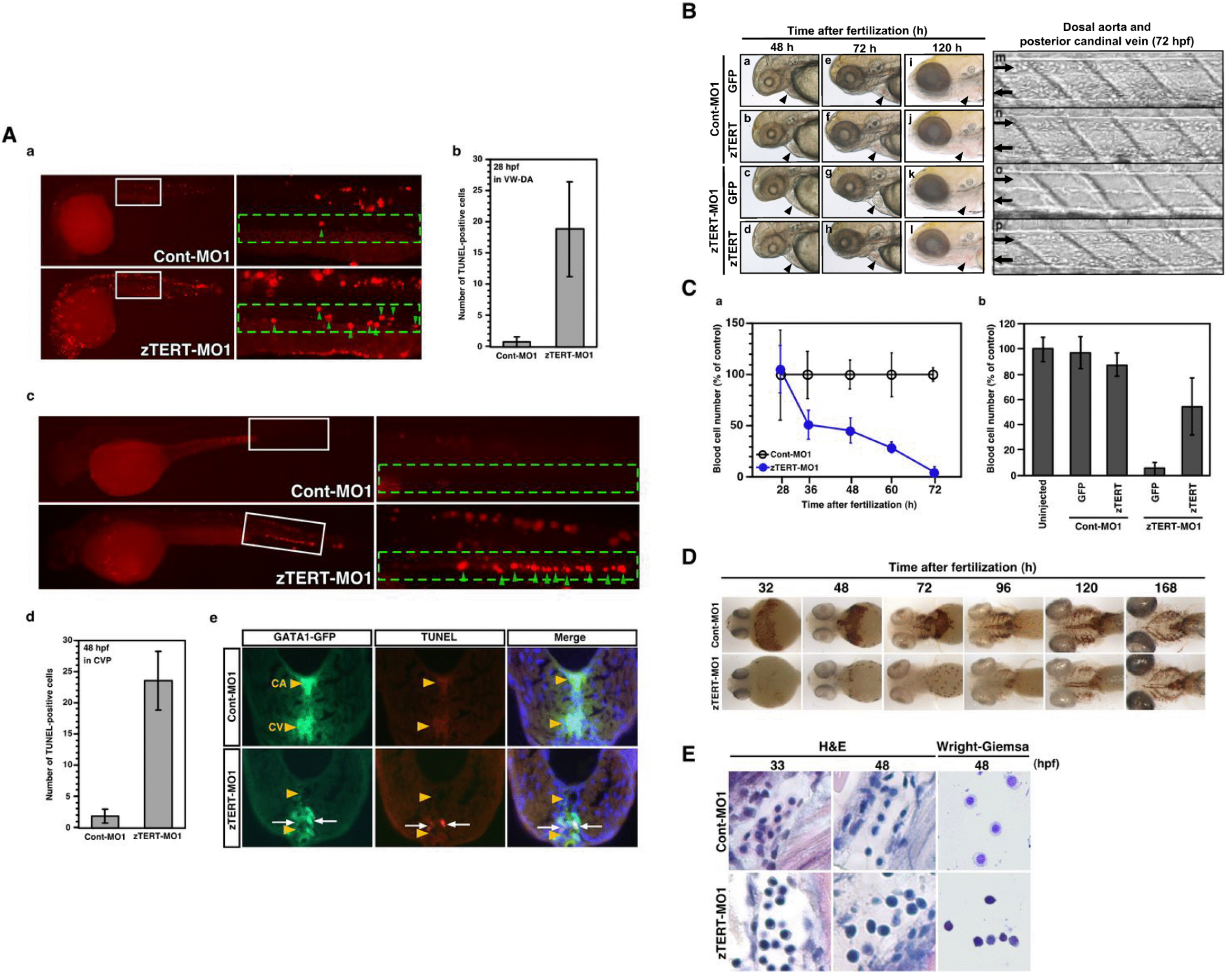
The knockdown of TERT in the zebrafish embryo results in severe cytopenia and in the impaired differentiation of hematopoietic cells. **(A)** Induction of apoptosis in hematopoietic cells of the zebrafish embryo. Both a low magnification of the whole body and higher magnification of the trunk region are shown in (a) and **(c)**, respectively. White squares in the low magnification images designate the regions shown in the higher magnification images in the adjoining right panels. **(a)** Apoptotic cells were detected by a TUNEL assay of the ventral wall of the dorsal aorta (VW-DA) (shown by green arrow heads in the right panel) at 28 hpf. **(b)** Quantification of the TUNEL-positive cells in the ventral wall of dorsal aorta (VW-DA) at 28 hpf. The number of TUNEL-positive cells was estimated within the gated area indicated by the green dashed rectangle at the upper yolk extension. **(c)** Apoptotic cells were detected by a TUNEL assay of the caudal venous plexus (CVP) (shown by green arrow heads in the right panel) at 48 hpf. **(d)** Quantification of the TUNEL-positive cells detected in the CVP at 48 hpf. The quantity of TUNEL-positive cells was assessed.within the gated area indicated by the green dashed rectangle at the anatomical CVP region. **(e)** Detection of apoptosis in *gata-1*^*GFP*^-positive hematopoietic cells. Transverse sections through the trunk region of 48-hpf *gata-1*^*GFP*^ embryos with the dorsal up are shown. The caudal artery (CA; upper) and caudal vein (CV; lower) are shown by orange arrow heads in the panels. By TUNEL assay, *gata-1*^*GFP*^-positive apoptotic cells in the CV are evident and indicated by white arrows. **(B)** Lateral views of 48, 72 and 120 hpf embryos following the co-injection of zTERT-MO1 (or Cont-MO1) and GFP-zTERT-cDNA (or GFP-cDNA) expression vectors **(a–l)**; black arrowheads indicate the heart regions. Bright field pictures of blood cells in trunks of 72 hpf embryos after co-injection of zTERT-MO1 or Cont-MO1 and either a GFP-cDNA or GFP-zTERT-cDNA vector **(m–p)**. The upper vessel is the dorsal artery (from left to right arrows) and the lower vessel is the posterior cardinal vein (from right to left arrows). **(C)** Quantitation of the circulating blood cell number in zTERT-MO- (blue circle) versus Cont-MO- (black open circle) injected embryos during 28–72 hpf **(a)**. **(b)** Calculation of the percentage of the control circulating blood cell numbers at 72 hpf after co-injection of zTERT-MO or Cont-MO and either a GFP-control or GFP-zTERT-cDNA vector. Blood cell numbers were determined for 10 embryos from each group. **(D)** Whole-mount o-dianisidine staining for heme detection in uninjected, Cont-MO1- and zTERT-MO1-injected embryos during 32–168 hpf. Blood flow over the yolk sac and in the tail vessels results in brown staining in wild type (data not shown) and Cont-MO1-injected embryos during 32–168 hpf (ventral view). **(E)** H&E staining of blood cells in tissue sections of the arteries or veins of Cont-MO1- and TERT-MO1-injected embryos at 33 and 48 hpf, and Wright-Giemsa staining of isolated blood cells from Cont-MO1- and TERT-MO1-injected embryos at 48 hpf‌‌.

**Fig 5:**
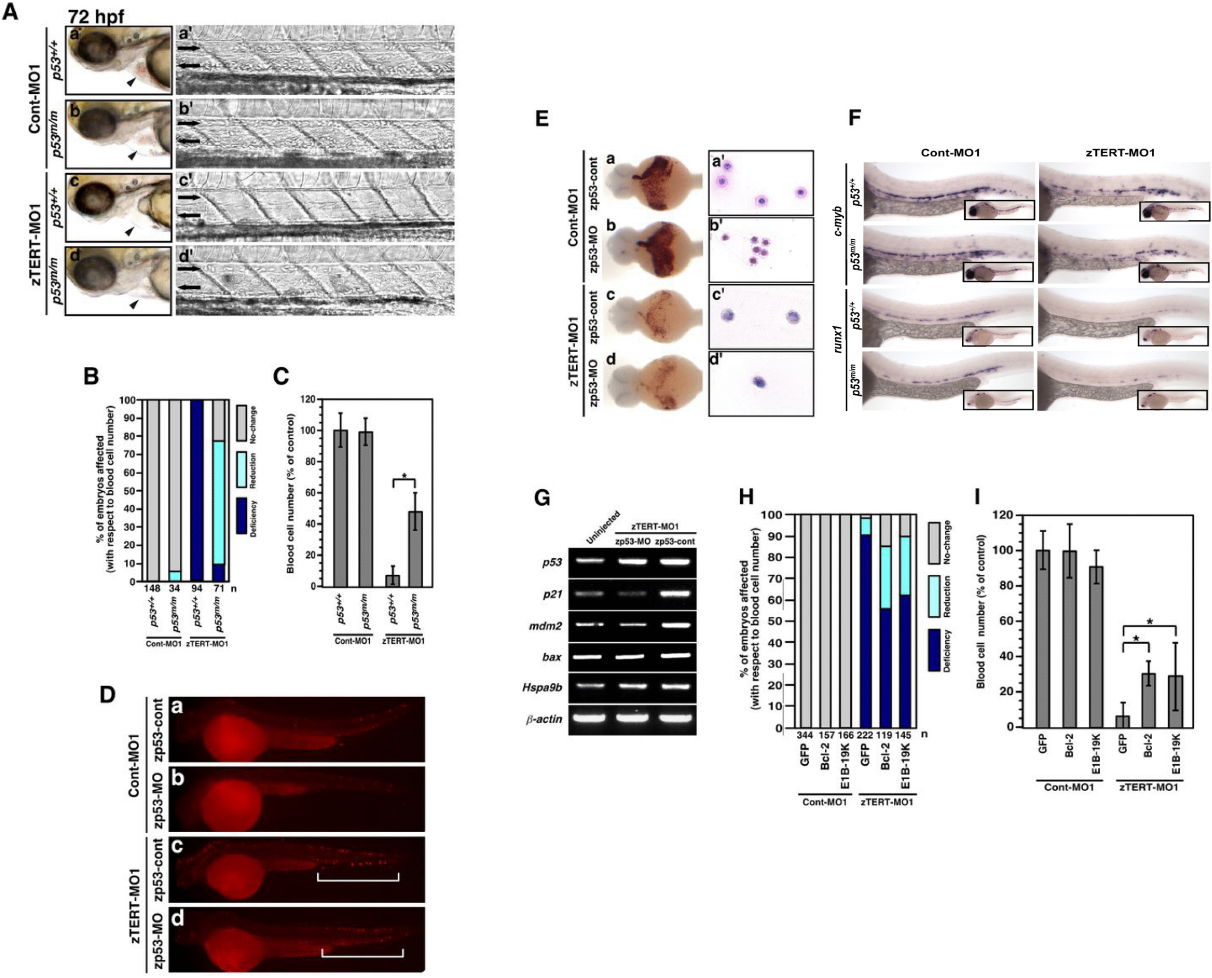
Rescue of cytopenia, but not anemia, in zTERT morphant embryos with a p53-deficient background. **(A)** Lateral views (anterior to left) of wild-type (*p53*^*+/+*^) and homozygous *p53*^*M214K*^ mutant (*p53*^*m/m*^) embryos injected with TERT-MO1 **(a–d)**. Arrowheads indicate the heart regions, including the blood (a’–d’) and views of the artery and veins (anterior to left) in the trunk at 72 hpf. **(B)** Scoring system based on the number of circulating blood cells at 72 hpf after injection of zTERT-MO1 or Cont-MO1 into *p53*^*+/+*^ and *p53*^*m/m*^ embryos. We divided the embryos into three classes based on their flowing blood cell number: i) indistinguishable from the control (>90%; no change) as indicated by the gray bar, ii) cell number reduction compared with the control (10–90%; reduction) as indicated by the light-blue bar, and iii) severely deficient or almost no flowing blood cells (<10%; deficiency) as indicated by the dark-blue bar. **(C)** Percentages of the control levels of circulating blood cell numbers at 72 hpf after the injection of zTERT-MO1 or Cont-MO1 into *p53*^*+/+*^ and *p53*^*m/m*^ embryos. Blood cell numbers were counted in 10 embryos from each group. ^*^P < 0.01 (Student t-test). **(D)** Whole-mount TUNEL staining in control and zTERT-knockdown embryos coinjected with either zp53-MO- or zp53-control-MO at 48 hpf **(a–d)**. A representative region of TUNEL-positive cells is indicated by the brackets **(c, d)**. **(E)** Whole-mount o-dianisidine staining of hemoglobin in control and TERT-knockdown embryos coinjected with either zp53-MO or zp53-control-MO at 48 hpf. The intensity of the blood flow color over the yolk indicates the hemoglobin concentration **(a–d)**. Wright-Giemsa staining of isolated blood cells from Cont-MO1- and zTERT-MO1-injected embryos in a p53-deficient background at 48 hpf (a’–d’). **(F)** Whole-mount in situ hybridization of control and TERT-knockdown embryos for *c-myb* and *runx1* expression in *p53*^*+/+*^ and *p53*^*m/m*^ embryos. The expression in the arterial region is indicated by arrowheads. **(G)** Altered expression levels of the indicated genes in TERT-deficient embryos in a p53-deficient background. Genes involved in the p53 pathway were analyzed by single-embryo RT-PCR. Similar results (data not shown) were obtained from this analysis of a number of individual embryos (more than 10 embryos for each gene). **(H)** Scoring of the number of circulating blood cells at 72 hpf after co-injection of zTERT-MO1 or Cont-MO1 and either GFP-, hBcl-2-, or E1B-19K-cDNA vectors. Embryos are classified as in **(B)**. **(I)** Circulating blood cell numbers as a percentage of the control at 72 hpf after co-injection of zTERT-MO1 or Cont-MO1 and either GFP-control, hBcl-2, or E1B-19K expressing vectors. Blood cell numbers were counted in 10 embryos for each group. ^*^P < 0.01 (Student t-test).

## Supporting information

S1 File Original images underlying Fig 2B panels e-h, and original quantitative data underlying Figs 2A (panel b) and 2C. (PDF)

S2 File Original images underlying the *runx1* zTERT-MO1 panels of Fig 5F, and original quantitative data underlying Figs 5B-C and 5H. (PDF)
