# Supplementary material for: A Non-Canonical Function of Zebrafish Telomerase Reverse Transcriptase Is Required for Developmental Hematopoiesis
Source: PLoS One. 2008 Oct 10;3(10):e3364. doi: 10.1371/journal.pone.0003364 (PMC2561060; doi:10.1371/journal.pone.0003364)
Supplement: Text S1 — (0.10 MB DOC) [file pone.0003364.s001.doc]

**Supporting Information *for PLoS ONE***

**A Non-Canonical Function of Zebrafish Telomerase Reverse Transcriptase is Required for Developmental Hematopoiesis**

Shintaro Imamura, Junzo Uchiyama, Eriko Koshimizu, Jun-ichi Hanai, Christina Raftopoulou, Ryan D. Murphey, Peter E. Bayliss, Yoichi Imai, Caroline Erter Burns, Kenkichi Masutomi, Sarantis Gagos, Leonard I. Zon, Thomas M. Roberts, and Shuji Kishi*

*Correspondence should be addressed to:

Shuji Kishi, M.D., Ph.D.

Schepens Eye Research Institute,

Department of Ophthalmology, Harvard Medical School

20 Staniford Street, Boston, MA 02114

Tel: 617-912-0200

Fax: 617-912-0101

E-mail: [s.kishi@mac.com](mailto:s.kishi@mac.com); shuji.kishi@schepens.harvard.edu

**Supporting Information (Results)**

**Molecular cloning of zebrafish TERT**

To clone the full-length zTERT cDNA, we utilized the TBLASN program (National Cancer for Biotechnology Information) and performed searches of the public databases harboring the zebrafish genome (Sanger Institute). Two zebrafish EST clones were identified (GenBank accession no. CN505902 and CO81403) and subsequently used to isolate the full-length zTERT cDNA of 2,287 bp by RT-PCR. We ascertained its homology to human TERT (hTERT) by sequencing. The 3’-proximal region was obtained using by 3’-RACE (see Materials and Methods). We confirmed high homology between the functional domains of zTERT and hTERT (GenBank accession no. AY007685) (**Figure S1**), with the N-termini containing the TR-binding and RT domains showing the greatest conservation. The zTERT protein shows an approximately 50% identity with hTERT within these functional domains, while exhibiting only 22% identity outside these regions. Importantly, the amino acids identified by mutational analysis that are essential to confer a dominant-negative function in hTERT are conserved in zTERT. In addition, the overall primary structure of zTERT based on its amino acid sequence resembles that of the human, mouse, chicken, *Xenopus*, and Fugu TERT proteins (**Figure S2A**). To clarify the evolutionary relationships between zTERT and TERT proteins from other species, a phylogenetic tree was constructed by comparing the amino acid sequences of the full length proteins (**Figure S2B, C**). zTERT was found to be more closely related to fugu TERT than to its human or mouse counterparts. Radiation hybrid (RH) mapping was then used to assign the zTERT gene to linkage group 19. While the studies presented here were under preparation, a full-length zTERT cDNA sequence was submitted to the GenBank by Xie et al. (accession no. ABM92944) and published by Lau et al. [1].

**Expression profile of zebrafish TERT mRNA and assay of telomerase activity during both early embryonic development and in adult tissues**

To determine the zTERT expression pattern during the early stages of zebrafish development, we performed in situ hybridization, semi-quantitative RT-PCR, and TRAP analyses of zebrafish embryos (**Figure S3A-C**). Whole-mount in situ hybridization assays were performed using DIG-labeled sense and antisense RNA probes (**Figure S3A**). zTERT mRNA is maternally provided and ubiquitously expressed at < 6 hpf. No significant staining was detectable using a sense control RNA probe at any developmental time point tested. As a general time course, zTERT expression can be observed between 0.2 hpf (1-cell), 0.75 hpf (2-cell stage), and 3 hpf (1000-cell stage), and is then almost undetectable at 6 hpf (50%-epiboly stage). Following 9 hpf (90%-epiboly stage), zTERT mRNA expression is again observed, and increases dramatically by 16 hpf (14-somite stage). By 23 hpf, zTERT mRNA is expressed strongly in the eye, midbrain, hindbrain, and tail, but relatively weakly in the trunk. At 48 hpf, some expression of zTERT is retained in the eye (lens) and midbrain. Single-embryo RT-PCR analysis was performed to semi-quantitatively assess levels of embryonic zTERT mRNA expression (**Figure S3B**). The detection of zTERT expression before the 3 hpf stage indicates the presence of maternally derived mRNA. The onset of zygotic transcription of zTERT is delayed until 6 hpf. Thus, zTERT mRNA significantly increases between 12 to 14 hpf. However, the results of our TRAP assays demonstrate that zTERT proteins are maintained at constant levels between the oocyte to 3 hpf stages, and increase from 6 hpf and are maintained at high levels by 72 hpf (**Figure S3C**). The expression of zTERT mRNA in a variety of zebrafish adult tissues was also investigated by RT-PCR (**Figure S3D**). Though zTERT mRNA expression is detectable in all adult tissues, the levels of mRNA differ in each organ. zTERT mRNA is at its highest levels in the ovary and testis, followed by the intestine, kidney, muscle, and liver, and at its lowest levels in the blood, followed by the eye, heart, brain, and skin.

We further examined whether regenerated zebrafish organs had high levels of zTERT mRNA. Zebrafish is a powerful animal model system for studies of tissue regeneration. Taking advantage of the easily accessible and visible fin regeneration in this species, we monitored for differences in telomerase expression and activity between resting and regenerating fins in identical animals. After amputation of the caudal fins, the amputated tissues were kept frozen, whereas the fish were maintained to observe fin regeneration over 3 days. The expression of zTERT was found to be detectable by RT-PCR at higher levels in the regenerated fin than in the original resting fin (**Figure S3D**, the rightmost two lanes).

We also examined the telomerase activity levels in a variety of other adult zebrafish tissues (**Figure S3E**). By TRAP assay we found that telomerase activity is extremely high in the ovary and testis, which is consistent with their mRNA expression levels, followed by liver, muscle, and kidney. In contrast, this activity is fairly weak in the blood, followed by eye, heart, brain, intestine, and skin. The high levels of zTERT mRNA expression were thus consistent with the high telomerase activity particularly in the ovary and testis. Regenerating fin had more abundant telomerase activity than resting fin, which is also consistent with their mRNA levels. On the other hand, the intestine had relatively low telomerase activity levels compared with the zTERT mRNA levels in this organ. These results suggest that telomerase activity is not only determined by, and does not correlate completely, with the zTERT mRNA expression levels and that there may post-translational modifications, possibly proteolytic remodeling, that contribute to the stability and enzymatic activity levels of the TERT protein [2,3,4,5,6]. Alternatively, the subsequent incorporation of other protein components through epigenetic effects (e.g. by methylation or phosphorylation) may be required to form a biologically active holoenzyme of TERT. These putative post-translational events need to be elucidated in further studies that compare zebrafish with other species.

**Mutant zTERT and their different functional roles**

One might think that these mutants (RNA-binding mutant: ΔTR-zTERT; catalytically inactive mutant: CD-zTERT) should have the same defective function because either of them abolishes ‘authentic’ telomerase activity. To resolve any confusion about this matter, it should be noted that the incidence that both mutants abolish ‘authentic’ telomerase activity does not necessarily preclude that they could have the different functions.

Our data suggest that the ΔTR-zTERT mutant still has an alternative function different from the CD-zTERT mutant with regard to its unforeseen activity independently of the telomerase RNA and conventional TERT function. The CD-zTERT mutant does not have this activity due to the point mutations in the catalytic domain. This is why the double mutant, CD-ΔTR-zTERT which has the same point mutations in the aspartate residue of its catalytic domain (See our scheme of the CD-ΔTR-zTERT mutant in **Figure 6A**) completely abolishes the ability to selectively rescue the blood cell phenotype.

It is clear that our catalytically inactive (defective) zTERT mutant (CD-zTERT) significantly loses the ability to rescue hematopoietic phenotypes (**Blue bars** in panels ‘**a**’ and ‘**d**’ of **Figure S10** and **Figure S11**). In contrast, the RNA-binding domain deletion mutant (N-terminal deletion) of zTERT (ΔTR-zTERT) has the same ability to rescue the phenotypes in comparison with wild-type zTERT although ΔTR-zTERT as well as CD-zTERT has no authentic telomerase activity by TRAP assay (**Orange bars** in panels ‘**b**’ and ‘**e**’ of **Figure S10** and **Figure S11**). These results suggest that another ‘telomerase RNA-independent’ alternative function of the catalytic domain (possibly certain enzymatic activity) of TERT is responsible for the observed hematopoietic functions and phenotypes.

To further easily clarify this issue, we made a supplemental **Table S1** depicting the comparison between ΔTR-zTERT and CD-zTERT. The point mutant (e.g., CD-zTERT) in the catalytic RT domain (any residue of three ‘aspartate’ within motif A and C) loses its any enzymatic function of TERT. Importantly, the double mutant (CD-ΔTR-zTERT) which has both ‘N-terminal deletion’ and ‘catalytically inactive point mutations’, was again unable to rescue hematopoietic phenotypes (**Light blue bars** in panels ‘**b**’ and ‘**e**’ of **Figure S10** and **Figure S11**).

A template and RNA-independent alternative enzymatic activity of TERT has been already reported [7]. On the other hand, most recently non-enzymatic functions of TERT protein have been also demonstrated [8]. However, in contrast to our results on CD-zTERT, Choi et al. showed that their catalytically inactive *TERT* mutant (*TERT^ci^*) retained a full activity of wild-type TERT in their system (i.e., in hair follicle stem cell activation and keratinocyte proliferation). Thus, presumably there are at least a couple of different alternative functions of TERT. Obviously further studies will be necessary to determine the molecular mechanism of these non-canonical functions of TERT.

There are at least two different ways to abolish conventional RNA-dependent ‘authentic’ telomerase activity: one is a disruption (of the functional site) of the catalytic domain (e.g., our CD-zTERT as well as Choi et al.’s TERT^ci^), and the other is disruption of RNA-binding domain (e.g., our ΔTR-zTERT). The former could retain non-enzymatic (“non-canonical”) function, but the latter can potentially sustain a RNA-independent alternative (“non-canonical”) activity.

In summary, with respect to this evident non-canonical function of TERT which we found during zebrafish early development, it seems to be telomerase RNA-independent but catalytic domain-dependent as an N-terminal (telomerase RNA-binding) domain-deleted mutant TERT, as well as wild-type TERT, can still function but point mutations in the catalytic domain ('aspartate' residue substitutions within motif A) cause significant loss/reduction of this functional role.

**Supporting Information (Materials and Methods)**

**Molecular cloning of zebrafish TERT**

mRNA was isolated from zebrafish embryos at 24 hpf using an mRNA purification kit (Amersham Biosciences). Poly(A) RNA was purified with oligo(dT)-resin from the Oligotex Direct mRNA Mini Kit (Qiagen). Double-stranded cDNA was synthesized using M-MLV reverse transcriptase (Promega), and PCR was performed using Ex Taq (Takara). The primer sequences are as follows: forward (F): 5’-AGTCGTGCAGACTTTGGAGGAGTT-3’, reverse (R): 5’-TGTAAACGGCCTCCACAGAGTTCA-3’. For PCR, the samples were amplified by 30-cycles of 30 sec at 94°C, 30 sec at 60°C, and 4 min at 72°C. The amplified DNA fragments were then purified by excision from agarose gels and cloned into the pCRII-TOPO cloning vector (Invitrogen). Nucleotide sequences were determined with a DNA sequencer (ABI 3700, AME Bioscience) and the ABI BigDye V3.1 cycle sequencing system. To analyze the full-length sequence of the zebrafish TERT cDNA, 3’- rapid amplification of cDNA ends (3’-RACE) was performed with a Generacer kit (Invitrogen). The reverse primer (5’-GTGGATGTGAGCGGAGCGTATGACAGT-3’) and reverse nested primer (5’-GTCATGAAGAAGCTGCTGTCGGTGCTC-3’) were used to amplify the 3’-end portion of the zebrafish cDNA by PCR with an adapter primer in the kit (Invitrogen). PCR was then performed according to the manufacturer's instructions using 10 ng of cDNA template and 1 ng of each primer in a total volume of 50 μl per sample. The sample was then subjected to touchdown PCR (2 min at 94°C, 5 cycles of 30 sec at 94°C and 2 min at 72°C, 5 cycles of 30 sec at 94°C and 2 min at 70°C, and 25 cycles of 30 sec at 94°C, 30 sec at 65°C, and 120 sec at 72°C). Fragments were then subcloned and sequenced. Multiple sequence alignments were carried out using the MegAlign software (DNAStar), the identity matrix was calculated using the BioEdit 7.0.0 software program, and a phylogenetic tree was constructed by using the Mega 3.1 program (<http://www.megasoftware.net/>). Default parameters were used in each case.

### Microangiography

### Microangiography was performed as described previously [9], except that 0.02-μm fluorospheres with red fluorescence (Molecular Probes) in 2% bovine serum albumin were used for the injections.

**Supporting Information (Figure Legends)**

**Figure S1.** **Amino acid sequence of zTERT in comparison with other vertebrates.**

Amino acid sequence comparisons and determination of conserved regions between the zebrafish, human, mouse, chicken, *Xenopus* and Fugu TERT proteins. The zebrafish TERT (zTERT) amino acid sequence was predicted from its cDNA and aligned with respect to the human, mouse, chicken, *Xenopus*, and Fugu TERT protein sequences using the MegAlign software (DNAStar) with default settings. The outputs are displayed by the box-shading and amino acids that are identical in all six species are shaded in black. Comparisons between the amino acid sequences in regions v-I, v-II, v-III, v-IV v-V, v-VI, and v-VII, and motifs T, 1, 2, A, B’, C, D and E are shown by light blue boxes and the regions are indicated in red [2,10,11,12]. Three conserved Asp (D) metal-binding residues in the reverse transcriptase (RT) motifs A and C are denoted by red asterisks.

**Figure S2.** **Primary structure of zTERT in comparison with other vertebrates.**

(**A**) Schematic representations of TERT proteins from diverse species (zebrafish, human, mouse, chicken, *Xenopus* and Fugu). Bar; 200 amino acids (aa).

(**B**) Percentiles of the identities among the TERT amino acid sequences from the five different vertebrate species. The identity matrix was calculated using the BioEdit 7.0.0 program.

(**C**) A phylogenetic tree of the TERT proteins shown in **(A)** and **(B)** obtained by comparing the full length of amino acid sequences using the Mega 3.1 program.

**Figure S3. zTERT mRNA and telomerase activity levels during embryogenesis and in adult fish.**

**(A)** Whole-mount mRNA in situ hybridizations were performed at the indicated stages. All embryos are lateral views with the animal pole orientated upwards (0.2-6 hpf) and the head positioned to the left (12-48 hpf). Note that the expression of *TERT* mRNA is absent at 6 hpf, detectable at 12 hpf, and is at high levels, particularly in the eye, midbrain, and hindbrain, by 16 to 48 hpf. Sense probes were used as a negative control.

**(B)** The expression of zTERT mRNA was analyzed by RT-PCR during embryogenesis and was found to be decreased by 6 hpf, at high levels during 9-24 hpf, and decreased again by 72 hpf. Similar results were obtained for a number of individual embryos (more than 10 embryos at each time point).

**(C)** Telomerase activity in whole zebrafish embryos during early development assayed by TRAP. Similar results were obtained for at least 5 embryos at each time point.

(**D)** Detection of z*TERT* mRNA expression by RT-PCR. Representative results from three independent experiments are shown. Similar results were obtained in three-independent experiments by using different individual samples.

**(E**) Detection of telomerase activity by TRAP assay in adult zebrafish (12 months old) tissues. The average values of two-independent duplicated experiments are shown in the graph. C; negative control.

**Figure S4.**  **Knockdown of zTERT by zTERT-MO1 and zTERT-MO2.**

(**A**) Schematic diagram of a construct containing the translational initiation site of zTERT fused with GFP and the region corresponding to the zTERT-MO1 sequence within the 5’ end of zTERT.

(**B**) Schematic diagram of the zTERT-MO2-induced splicing defect. Total RNA was extracted from 24 hpf embryos after MO2 injection at the single (or two)-cell stage. The targeted fragment was then amplified by RT-PCR between exons 4 and 8 of zTERT (see the main Materials and Methods for the corresponding forward and reverse primers). DNA sequencing results show that the intron between exon 5 and 6 was not skipped during splicing in the MO2-injected embryos. The integration of this intact intron creates an in-frame stop codon (TAG) immediately behind exon 5, resulting in a truncated zTERT protein lacking most of its RT domain.

**Figure S5. Analyses of Telomere length in zTERT morphants.**

(**A**) Metaphase spreads from zebrafish embryos for quantitative telomere FISH (Q-FISH) analysis. **Metaphase chromosomes** from Cont-MO1-injected embryos (**a**), and zTERT-MO1-injected embryos (**b**), **for** telomere Q-FISH.

(**B**) Representative histograms showing the distribution of telomere lengths by telomere fluorescence intensity measurements of metaphase spreads from control and zTERT morphants (MO1 and MO2; 8 ng) at 24 hpf, as measured using the TFL-TELO software. Histograms of telomere Q-FISH for Cont-MO1 and zTERT-MO1 (**a**), and Cont-MO2 and zTERT-MO2 (**b**). The x-axis depicts the intensity of each signal as expressed in telomere fluorescence intensity units (TFU), and the y-axis shows the frequency of telomeres of a given intensity. The dashed orange line indicates 10 x 10^2^ TFU.

**(C)** Telomeric restriction fragment (TRF) analysis of DNA isolated from either uninjected, Cont-MO1-, or zTERT-MO1-injected embryos **(a)**, and from zebrafish genomic DNA digested by *DNase I* *in vitro* and control human DNA (DNA-high and DNA-low) **(b)**.

**Figure S6. Blood cell number and heme intensity in zTERT-MO2-injected embryos.**

(**A**) Lateral views of the trunk regions of 72 hpf embryos following injection of 0, 1.6, and 8.0 ng of zTERT-MO2. In these bright field pictures, blood cells in the trunks were observed upon a decrease in the injected MO concentrations. The upper vessel is the dorsal artery (left to right arrows) and the lower vessel is the posterior cardinal vein (right to left arrows).

(**B**) Quantitation of the circulating blood cell number in zTERT-MO2-injected embryos. The percentage of the circulating blood cell numbers at 72 hpf was calculated using uninjected samples (0 ng) as a control and were determined for 15 embryos from each group.

**(C)** Whole-mount o-dianisidine staining for the presence of heme in Cont-MO2- and zTERT-MO2-injected embryos at 48 hpf (8 ng MOs). Blood flow over the yolk sac and in the tail vessels results in brown staining in Cont-MO2-injected embryos at 48 hpf (ventral view; upper panel), but there is an obvious decrease in this staining following the MO2 injection (lower panel).

(**D**) Quantification of the heme staining intensity in embryos injected with Cont-MO2 or zTERT-MO2 (8 ng) at 48 hpf. Percentages of the control heme intensity in injected embryos.

(**E**) Wright-Giemsa staining of isolated blood cells from Cont-MO2- and TERT-MO2-injected embryos at 48 hpf (8 ng MOs).

**Figure S7. Whole-mount in situ hybridization of in zTERT-MO2-injected embryos.**

**(A)** The detection of primitive hematopoietic markers in embryos at the 20-somite stage (19 hpf) which are oriented with the anterior to the left in lateral (left panels) and dorsal views (right panels). Control (Cont-MO2) and TERT (zTERT-MO2) morphants (8 ng MOs) were analyzed for the expression of *scl* (zTERT-MO2; n = 51 of 54; 94%)*, lmo2* (zTERT-MO2; n = 48 of 55; 87%) and *gata-2* (zTERT-MO2; n = 50 of 53; 94%), which are early hematopoietic markers. The expression of *scl* and *lmo2* in the ICM and that of *gata-2* in the blood island is indicated by arrowheads.

**(B)** Embryos at 28-32 hpf are oriented with the anterior to the left in lateral views. Control (Cont-MO1) and TERT morphants (both zTERT-MO1 and MO2) (8 ng MOs) were analyzed for the expression of multiple hematopoietic cell lineage markers. Representative time points for the expression of *alas2* (32 hpf) (zTERT-MO2; n = 51 of 55; 93%), *runx1* (32 hpf) (zTERT-MO2; n = 47 of 50; 94%), and *c-myb* (32 hpf) (zTERT-MO2; n = 46 of 50; 92%) are shown. *Gata-1* (28 hpf) (zTERT-MO2; n = 46 of 53; 87%), *pu.1* (28 hpf) (zTERT-MO2; n = 44 of 51; 86%), *globin bE3* (28 hpf) (zTERT-MO2; n = 48 of 55; 87%), *l-plastin* (28 hpf) (zTERT-MO2; n = 51 of 58; 88%), *mpo* (28 hpf) (zTERT-MO2; n = 50 of 56; 89%), and *flk1* (28 hpf) (zTERT-MO2; n = 45 of 51; 88%) are not shown but the results of the expression analysis for these genes were found to be consistent between the MO1 and MO2 morphants. The expression of these genes in the arterial region is indicated by arrowheads

(**C**) Alterations of *CD41^GFP^*-positive cells in zTERT morphants. (**a**) zTERT-MO1 or MO2 was injected into *CD41^GFP^*-transgenic fish embryos. In comparison with the control (Cont-MO1), an apparent decrease of *CD41^GFP^*-positive cells was observed in zTERT morphants at 54 hpf. Reduction of *CD41^GFP^*-positive cells in the CVP are shown in the right panels. This decreased number of *CD41^GFP^*-positive cells in zTERT morphants (in both MO1 and MO2) was observed until 72 hpf. (**b**) Quantification of *CD41^GFP^*-positive cells in the CVP areas of control and zTERT morphants at 54 hpf.

**Figure S8. The effects of zTERT knockdown on the embryonic vasculature.**

0.02 μm beads were injected into *fli*^GFP^ transgenic embryos at 72 hpf. GFP signals (green) indicate the vascular structures, and the beads (red) correspond to the blood flow in the vasculature. These green and red signals merge as yellow.

**Figure S9. Rescue of blood cell number and heme intensity in zTERT-MO2-injected embryos.**

(**A**) Rescue of blood cell number by TERT expression in zTERT-MO2-injected embryos. (**a**) The blood cell number defect is rescued in zTERT morphants at 72 hpf following the injection of the indicated TERT constructs. (**b**) Percentage of control circulating blood cell numbers in embryos at 72 hpf after co-injection of several TERT constructs and either Cont-MO1, zTERT-MO1, or zTERT-MO2 at 72 hpf. Blood cell numbers were counted for 15 embryos in each group.

**(B)** Rescue of heme intensity by TERT expression in zTERT-MO2-injected embryos. (**a**) Rescue of blood cell appearance, visualized by whole-mount o-dianisidine staining for hemoglobin detection, in TERT-deficient embryos following the injection of several TERT constructs. Representative whole-mount o-dianisidine stained samples are shown. (**b**) Percentages of the control heme intensity in embryos at 72 hpf after injection of the indicated TERT constructs with Cont-MO2 or zTERT-MO2. Heme intensities were measured for 12 embryos in each group.

**Figure S10. Restoration of ineffective hematopoiesis in TERT-deficient embryos by the expression of zebrafish and human TERT.**

(A) Quantitation of the circulating blood cell number and heme intensity in zTERT-MO1-injected zebrafish embryos. zTERT-MO1 (0, 1.6, 4.0, or 8.0 ng) was co-injected with GFP or with WT-zTERT or CD-zTERT (**a, d**), ΔTR-zTERT or CD-ΔTR-zTERT (**b, e**), WT-hTERT or DN-hTERT (**c, f**).

(**B**) Quantitation of circulating blood cell number and heme intensity in zTERT-MO2-injected zebrafish embryos. zTERT-MO2 (0, 1.6, 4.0, or 8.0 ng) was co-injected with GFP or with the zTERT constructs as described in (**A**).

**Figure S11.** Telomerase activity **in TERT-deficient embryos by the expression of zebrafish and human TERT.**

Quantitation of telomerase activity in zTERT-MO1 or -MO2-injected zebrafish embryos. zTERT-MO1 or -MO2 (0, 1.6, 4.0, or 8.0 ng) were co-injected with GFP or with WT-zTERT or CD-zTERT (**a, d**), ΔTR-zTERT or CD-ΔTR-zTERT (**b, e**), WT-hTERT or DN-hTERT (**c, f**).

**References**

1. Lau BW, Wong AO, Tsao GS, So KF, Yip HK (2008) Molecular Cloning and Characterization of the Zebrafish (Danio rerio) Telomerase Catalytic Subunit (Telomerase Reverse Transcriptase, TERT). J Mol Neurosci 34: 63-75.

2. Kuramoto M, Ohsumi K, Kishimoto T, Ishikawa F (2001) Identification and analyses of the Xenopus TERT gene that encodes the catalytic subunit of telomerase. Gene 277: 101-110.

3. Keppler BR, Grady AT, Jarstfer MB (2006) The biochemical role of the heat shock protein 90 chaperone complex in establishing human telomerase activity. J Biol Chem.

4. Holt SE, Aisner DL, Baur J, Tesmer VM, Dy M, et al. (1999) Functional requirement of p23 and Hsp90 in telomerase complexes. Genes Dev 13: 817-826.

5. Kim JH, Park SM, Kang MR, Oh SY, Lee TH, et al. (2005) Ubiquitin ligase MKRN1 modulates telomere length homeostasis through a proteolysis of hTERT. Genes Dev 19: 776-781.

6. Martin-Rivera L, Herrera E, Albar JP, Blasco MA (1998) Expression of mouse telomerase catalytic subunit in embryos and adult tissues. Proc Natl Acad Sci U S A 95: 10471-10476.

7. Lue NF, Bosoy D, Moriarty TJ, Autexier C, Altman B, et al. (2005) Telomerase can act as a template- and RNA-independent terminal transferase. Proc Natl Acad Sci U S A 102: 9778-9783.

8. Choi J, Southworth LK, Sarin KY, Venteicher AS, Ma W, et al. (2008) TERT promotes epithelial proliferation through transcriptional control of a Myc- and Wnt-related developmental program. PLoS Genet 4: e10.

9. Weinstein BM, Stemple DL, Driever W, Fishman MC (1995) Gridlock, a localized heritable vascular patterning defect in the zebrafish. Nat Med 1: 1143-1147.

10. Nakamura TM, Morin GB, Chapman KB, Weinrich SL, Andrews WH, et al. (1997) Telomerase catalytic subunit homologs from fission yeast and human. Science 277: 955-959.

11. Bryan TM, Sperger JM, Chapman KB, Cech TR (1998) Telomerase reverse transcriptase genes identified in Tetrahymena thermophila and Oxytricha trifallax. Proc Natl Acad Sci U S A 95: 8479-8484.

12. Lingner J, Hughes TR, Shevchenko A, Mann M, Lundblad V, et al. (1997) Reverse transcriptase motifs in the catalytic subunit of telomerase. Science 276: 561-567.
