# Supplementary material for: A Non-Canonical Function of Zebrafish Telomerase Reverse Transcriptase Is Required for Developmental Hematopoiesis
Source: PLoS One. 2008 Oct 10;3(10):e3364. doi: 10.1371/journal.pone.0003364 (PMC2561060; doi:10.1371/journal.pone.0003364)
Supplement: Table S1 — (0.05 MB DOC) [file pone.0003364.s002.doc]

**Table S1 *Mutant zTERT and Their Different Functional Roles.***

| **Mutant zTERT**    **Functions** | **CD-zTERT**  Catalytically defective mutant (by point mutations in the reverse transcriptase motif A) | **ΔTR-zTERT**  RNA-binding domain deletion mutant (by N-terminal deletion) |
| --- | --- | --- |
| **i) Telomerase activity** | **No telomerase activity** is observed (Figure 6C, panel b), and it has dominant-negative function to telomerase (Figure 6C, panel a). | **No telomerase activity** is observed (Figure 6C, panel b). |
| **ii) Non-enzymatic functions and/or other catalytic activities** | No other catalytic and enzymatic activity is anticipated [1], ***but*** the point mutations may also induce unforeseen effects on the TERT protein conformation. | Template (i.e., telomerase RNA)-independent alternative catalytic activity such as terminal transferase is suspected [2], ***but*** non-enzymatic functions of RT domain of TERT protein is also needed to be considered. |
| **iii) Effects on hematopoietic phenotypes** | **No (/little) rescue of hematopoietic phenotypes** is observed in zTERT-depleted embryos. | **Rescue of hematopoietic phenotypes** (at the same level of **wild-type zTERT**) is observed in zTERT-depleted embryos. |

**Note:** The double mutant (**CD-ΔTR-zTERT**), which has both the ‘N-terminal deletion’ and ‘catalytically inactive point mutations’, is unable to rescue hematopoietic phenotypes, suggesting that the catalytic/enzymatic domain of TERT is functionally crucial for the blood phenotypes. See “Schematic representation of zebrafish TERT and its mutants” in **Figure 6A.**

**References**

1. Choi J, Southworth LK, Sarin KY, Venteicher AS, Ma W, et al. (2008) TERT promotes epithelial proliferation through transcriptional control of a Myc- and Wnt-related developmental program. PLoS Genet 4: e10.

2. Lue NF, Bosoy D, Moriarty TJ, Autexier C, Altman B, et al. (2005) Telomerase can act as a template- and RNA-independent terminal transferase. Proc Natl Acad Sci U S A 102: 9778-9783.
