# Supplementary figures and images for: A Non-Canonical Function of Zebrafish Telomerase Reverse Transcriptase Is Required for Developmental Hematopoiesis
Source: PLoS One. 2008 Oct 10;3(10):e3364. doi: 10.1371/journal.pone.0003364 (PMC2561060; doi:10.1371/journal.pone.0003364)

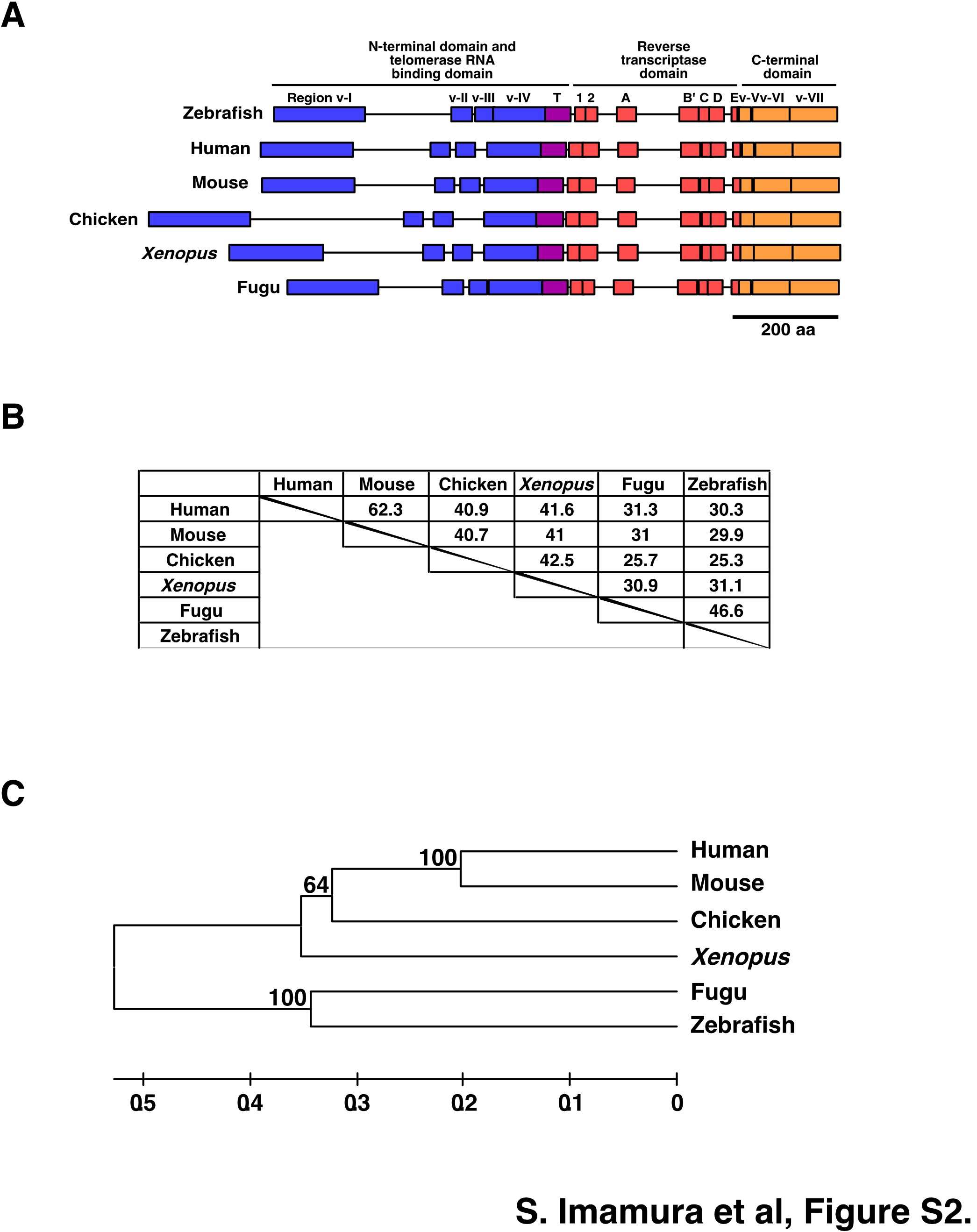

Supplement: Figure S2 — (0.31 MB TIF) [file pone.0003364.s004.tif]

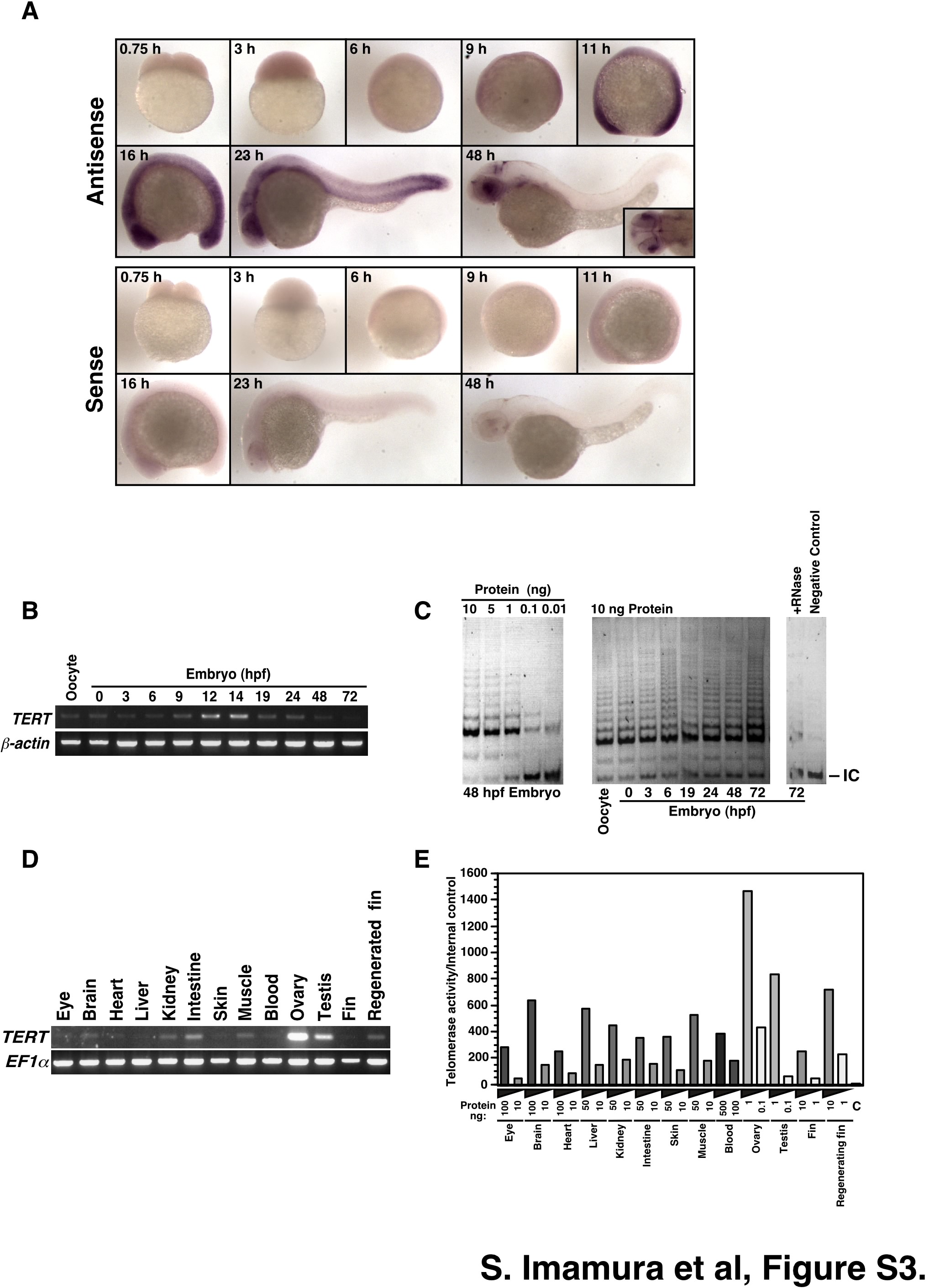

Supplement: Figure S3 — (0.52 MB TIF) [file pone.0003364.s005.tif]

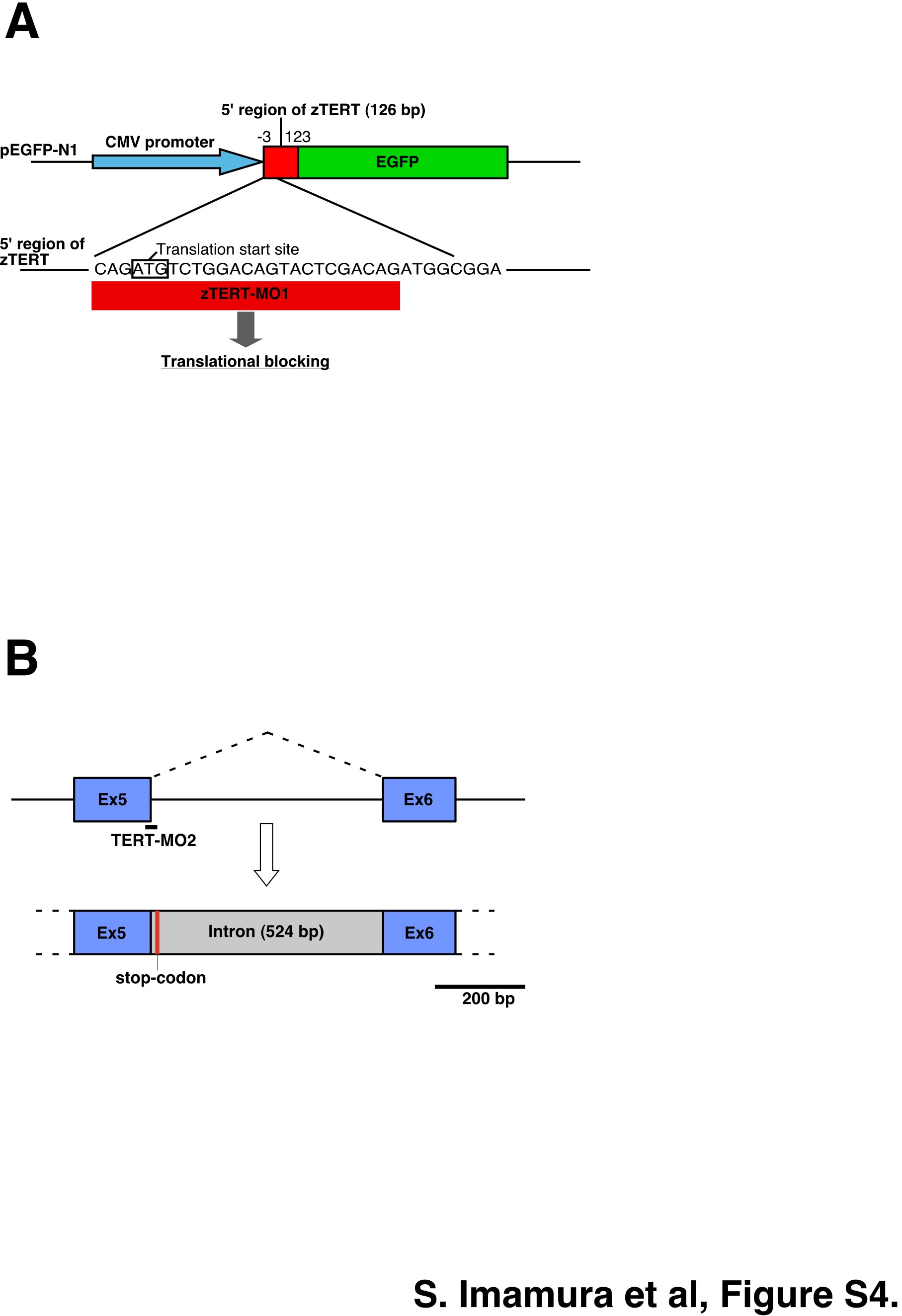

Supplement: Figure S4 — (0.15 MB TIF) [file pone.0003364.s006.tif]

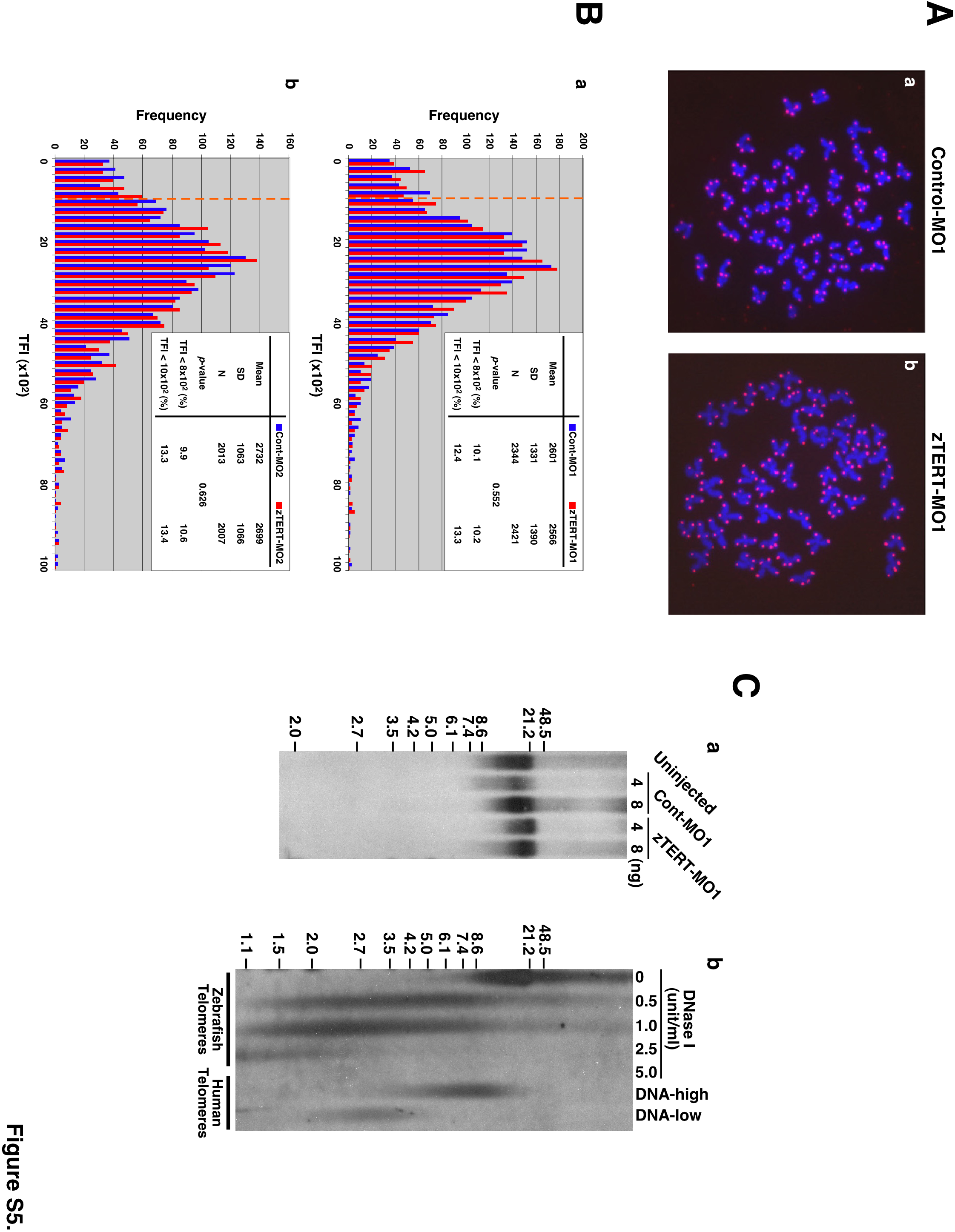

Supplement: Figure S5 — (2.38 MB TIF) [file pone.0003364.s007.tif]

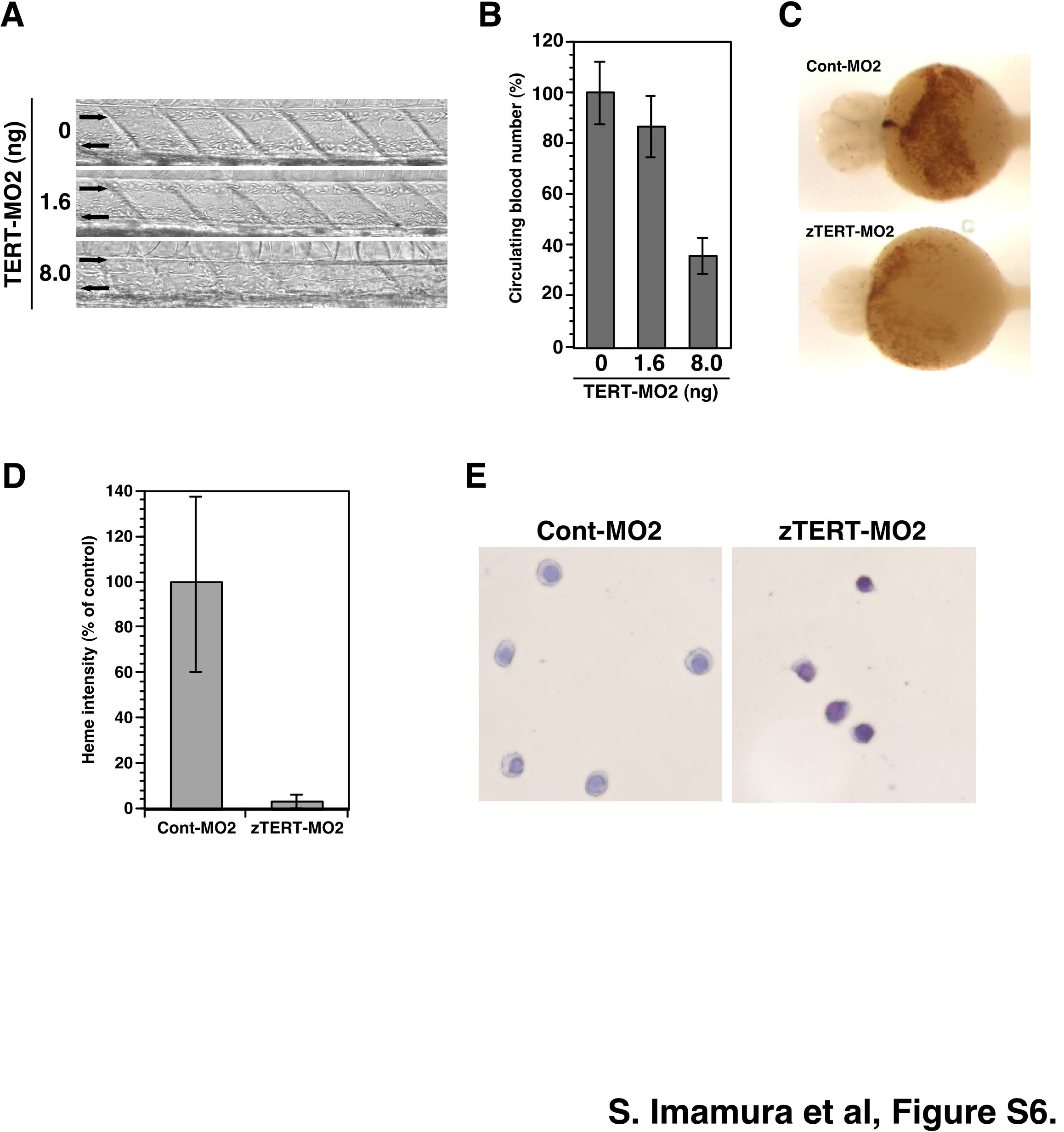

Supplement: Figure S6 — (0.37 MB TIF) [file pone.0003364.s008.tif]

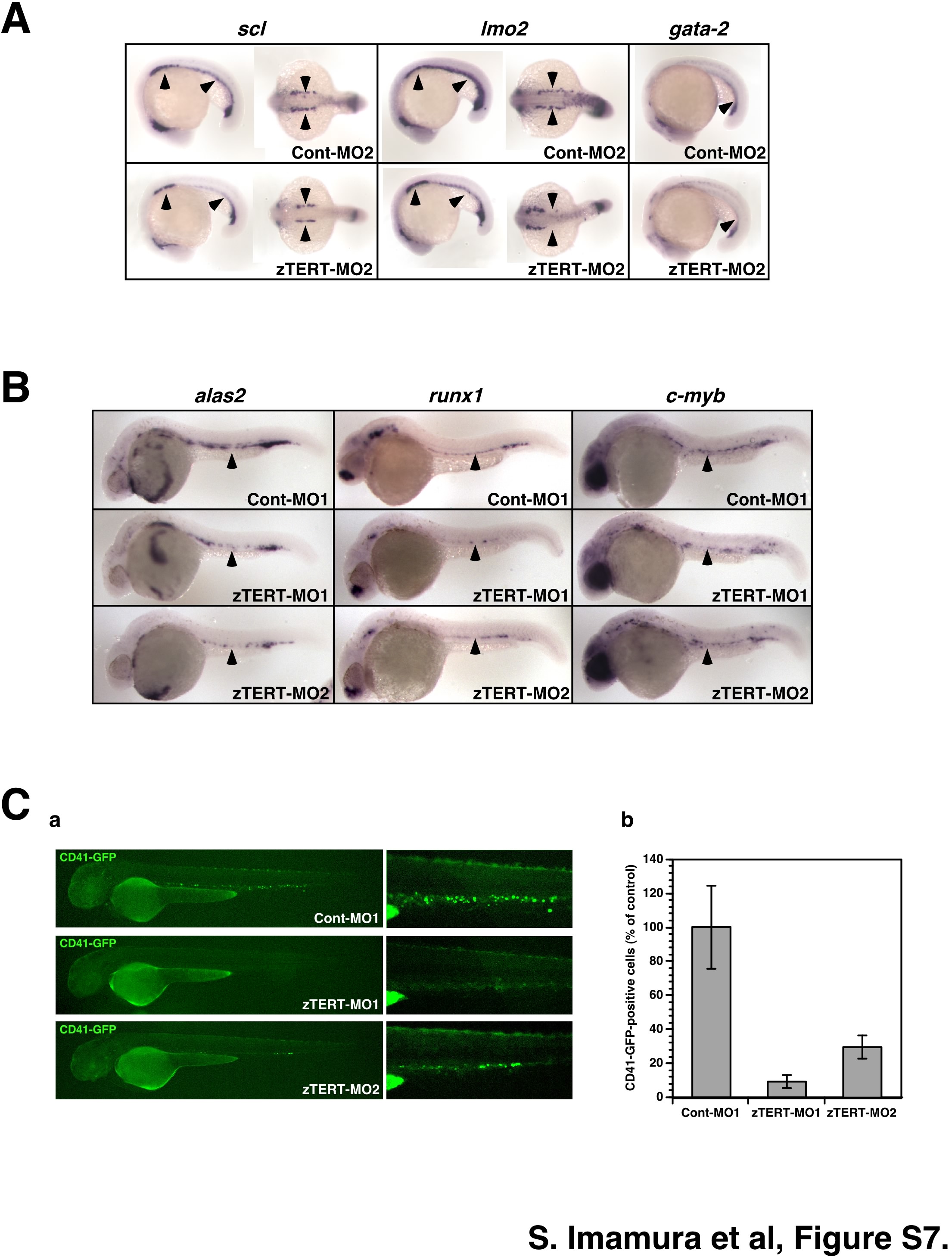

Supplement: Figure S7 — (0.63 MB TIF) [file pone.0003364.s009.tif]

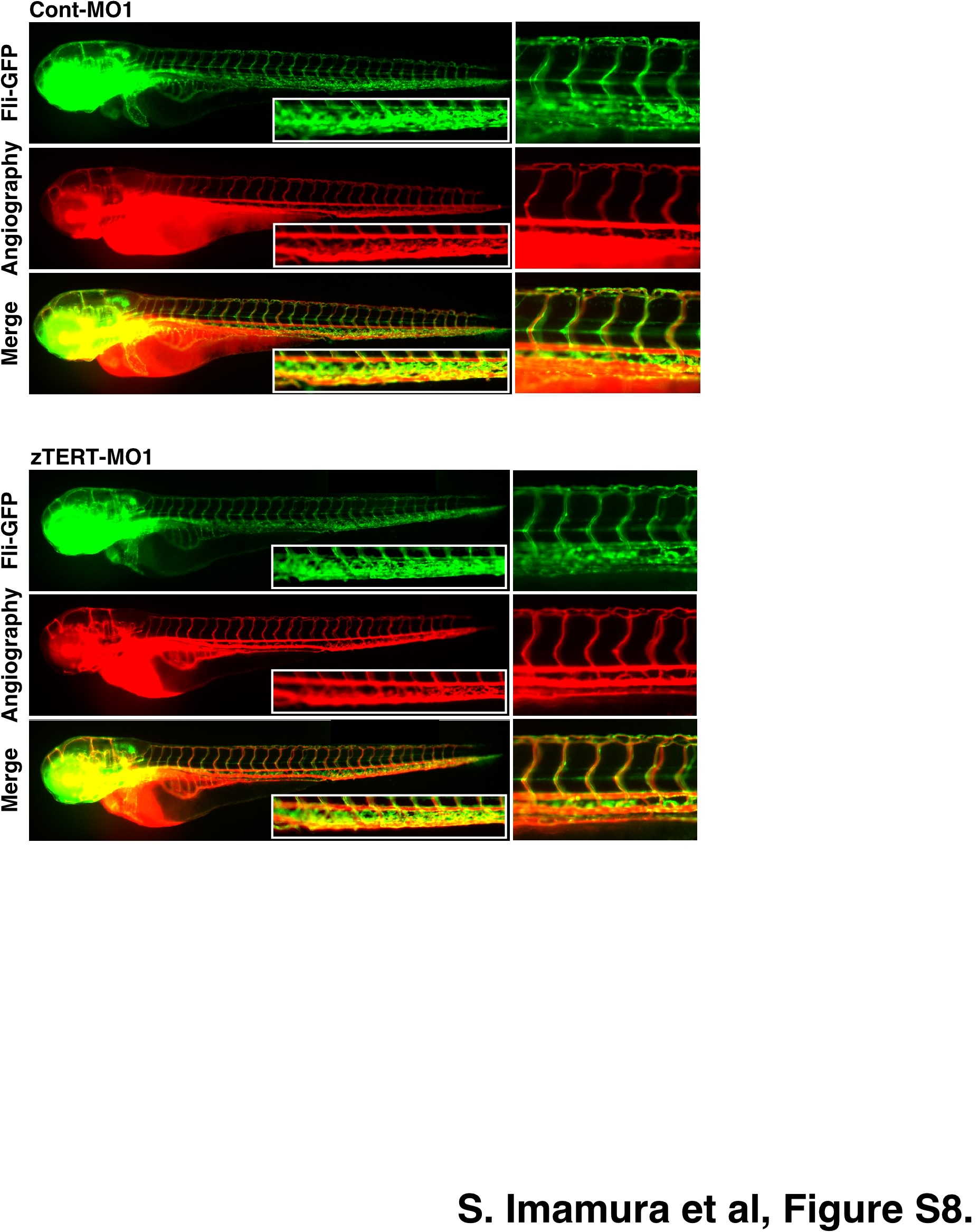

Supplement: Figure S8 — (0.41 MB TIF) [file pone.0003364.s010.tif]

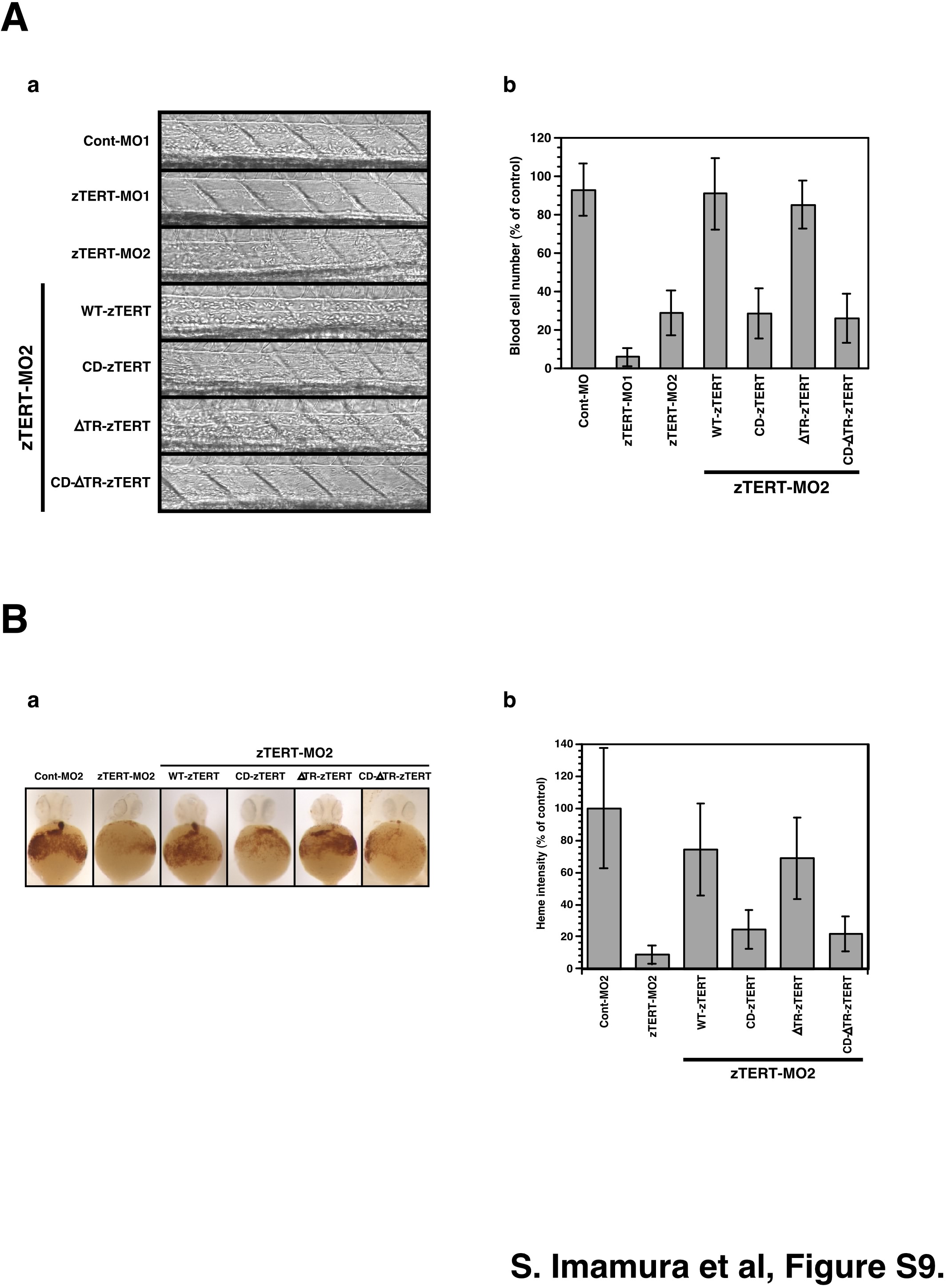

Supplement: Figure S9 — (0.44 MB TIF) [file pone.0003364.s011.tif]

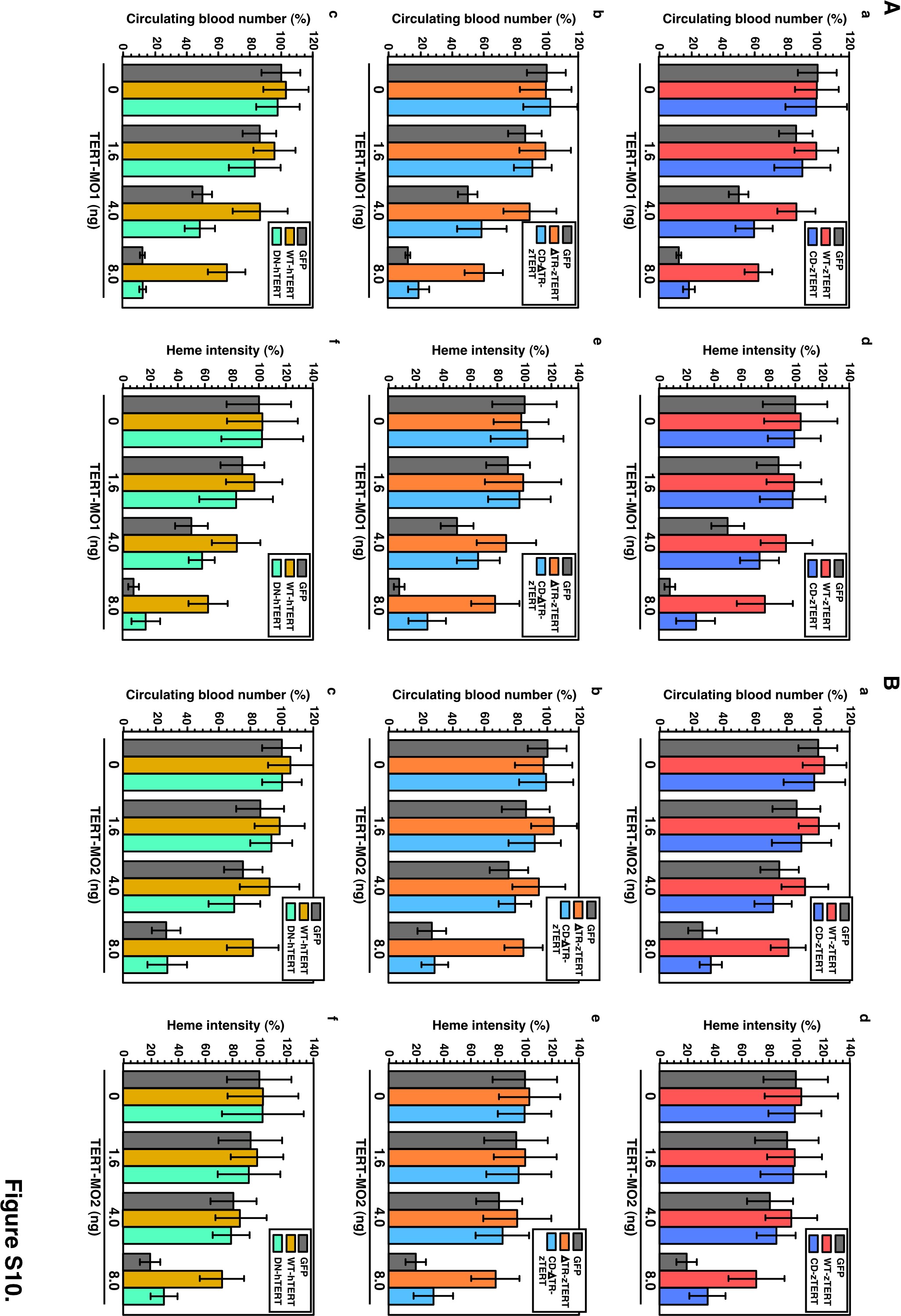

Supplement: Figure S10 — (0.69 MB TIF) [file pone.0003364.s012.tif]

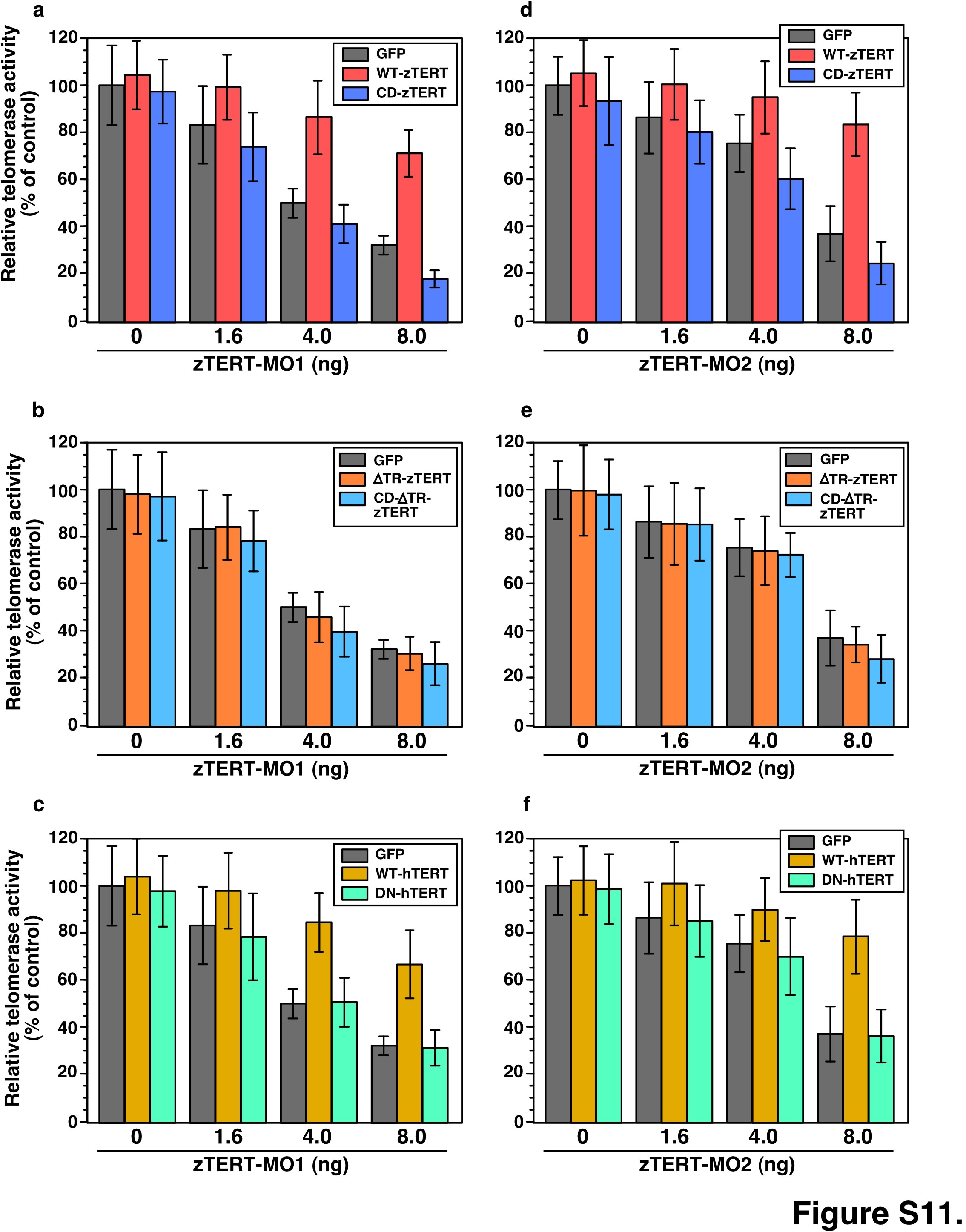

Supplement: Figure S11 — (0.56 MB TIF) [file pone.0003364.s013.tif]
