# Supplementary material for: A Non-Canonical Function of Zebrafish Telomerase Reverse Transcriptase Is Required for Developmental Hematopoiesis
Source: PLoS One. 2008 Oct 10;3(10):e3364. doi: 10.1371/journal.pone.0003364 (PMC2561060; doi:10.1371/journal.pone.0003364)
Supplement: Figure S1 — (1.05 MB PDF) [file pone.0003364.s003.pdf]

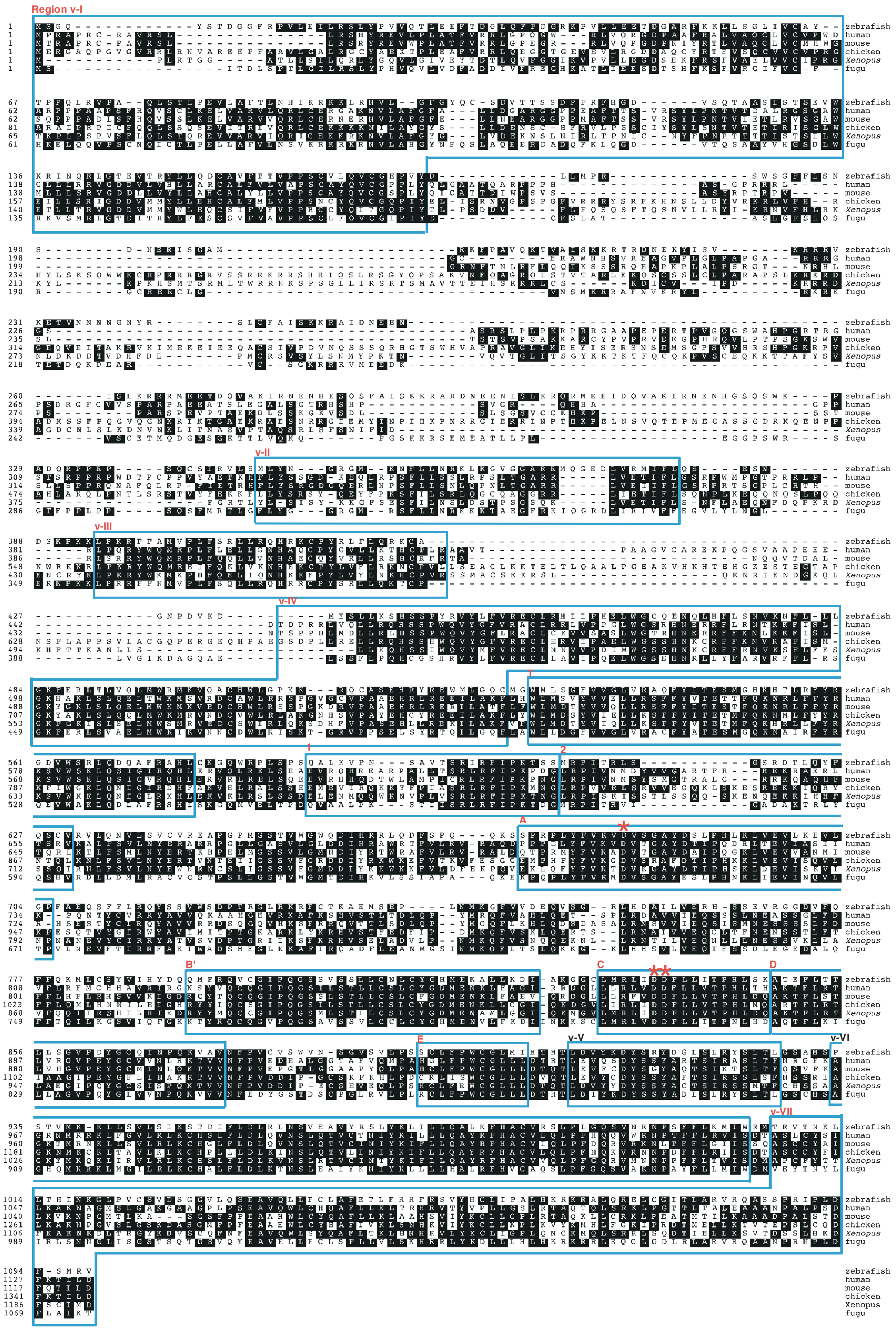

S. Imamura et al, Figure S1.
