# Supplementary material for: Correction: A non-canonical function of zebrafish telomerase reverse transcriptase is required for developmental hematopoiesis
Source: PLoS One. 2026 May 29;21(5):e0350446. doi: 10.1371/journal.pone.0350446 (PMC13221052; doi:10.1371/journal.pone.0350446)
Supplement: S1 File — (PDF) [file pone.0350446.s001.pdf]

Fig.2B

original

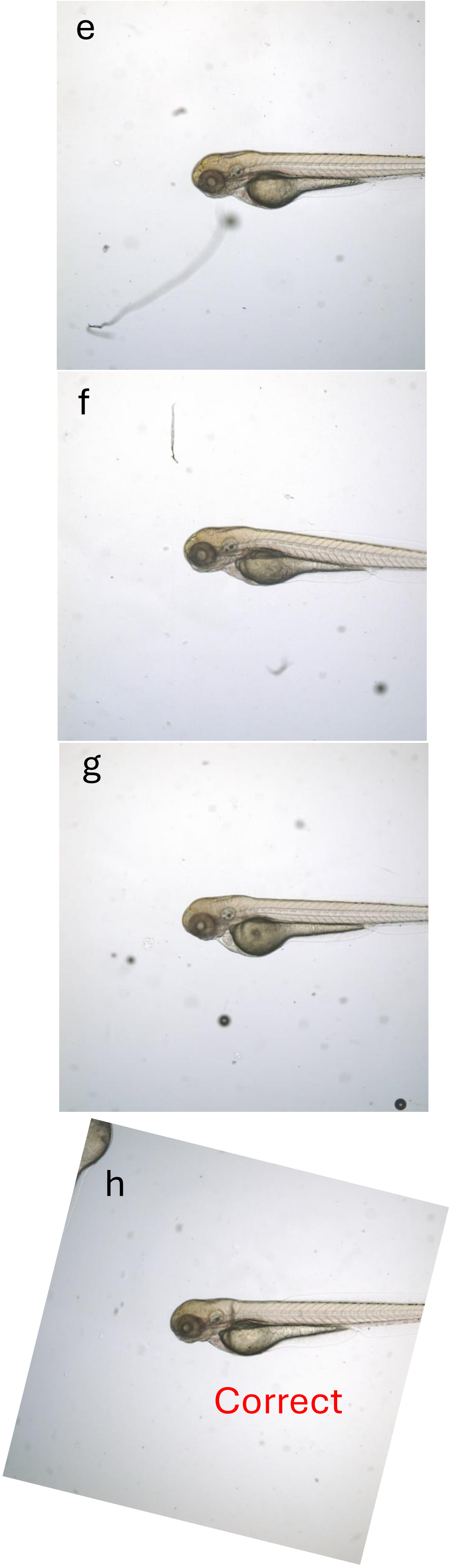

Fig.2A

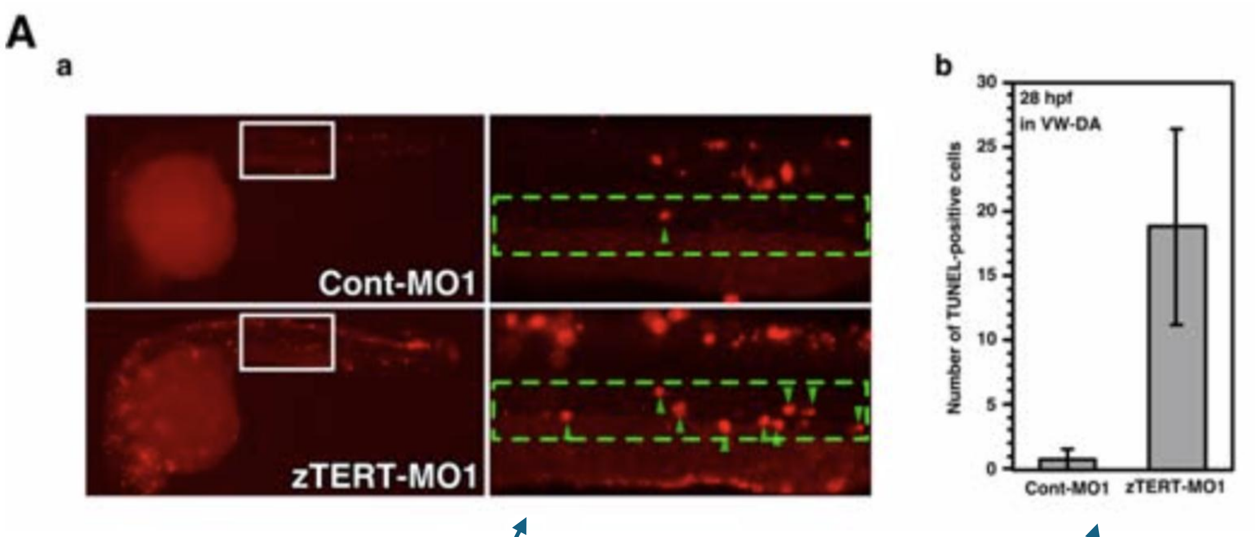

Original data

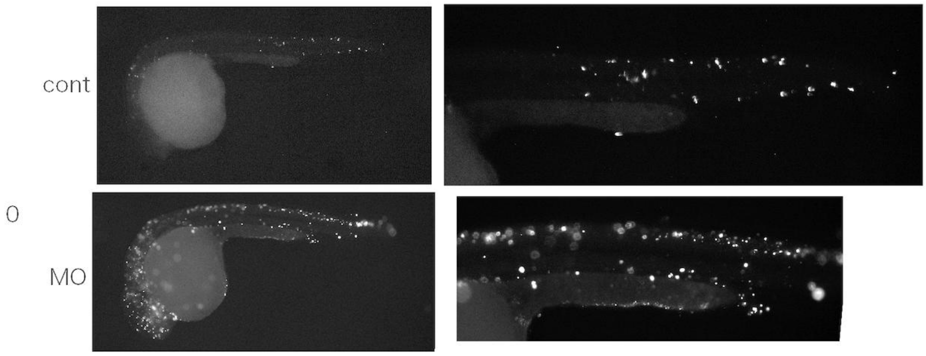

	TUNEL	
	cont	TERT
	2	19
	1	20
	1	30
	0	16
	0	9
	0	
	1	
mean	0.71428571	18.8
SD	0.75592895	7.59605161

Cont-MO1

zTERT-MO1

Fig.2C

C

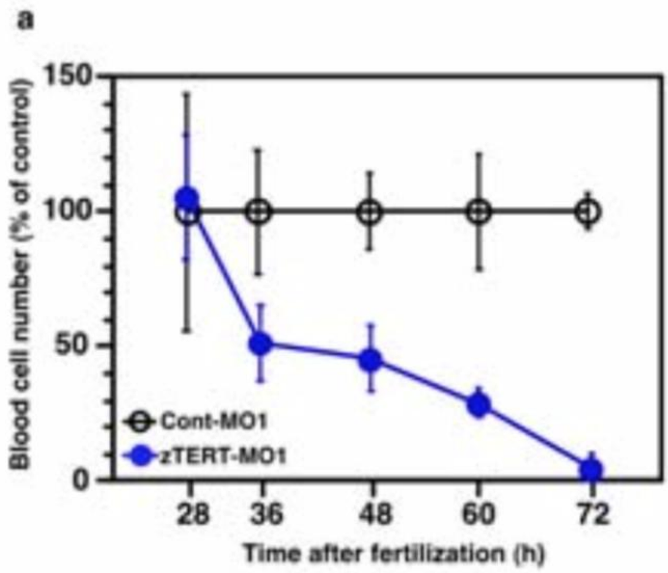

Deltagraph, March 10, 2006

TERT blood.dg6:データビュー

データ グラフ オーガナイザー インポート 更新 グラフ 再計算 数式作成 書式設定 ソート 行と列を転置

	ラベル	A	B	C	D	E	F	G	H	I	J
ラベル		28h cont	28h MO	36h cont	36h MO	48h cont	48h MO	60h cont	60 h MO	72h cont	72h MO
1		18	29	99	43	109	51	109	42	140	8
2		63	55	143	38	107	71	98	51	141	5
3		68	57	96	57	121	60	180	37	138	21
4		45	60	72	25	129	28	120	35	133	11
5		66	36	98	36	112	50	118	32	125	0
6		48	48	72	60	95	74	105	47	126	2
7		35	56	79	60	99	48	133	39	129	1
8		57	48	83	42	130	67	131	35	129	20
9		14	37	77	43	143	41	178	28	148	0
10		31	43	91	63	142	51	149		150	0
11											
12		Cont-MO1 28h	zTERT-MO1 28h	Cont-MO1 36h	zTERT-MO1 36h	Cont-MO1 48h	zTERT-MO1 48h	Cont-MO1 60h	zTERT-MO1 60h	Cont-MO1 72h	zTERT-MO1 72h
13											
14											
15											
16											
17											

This graph shows the blood cell counts for each experimental group. Values are expressed relative to the uninjected control, which was set to 100.

Fig.2C

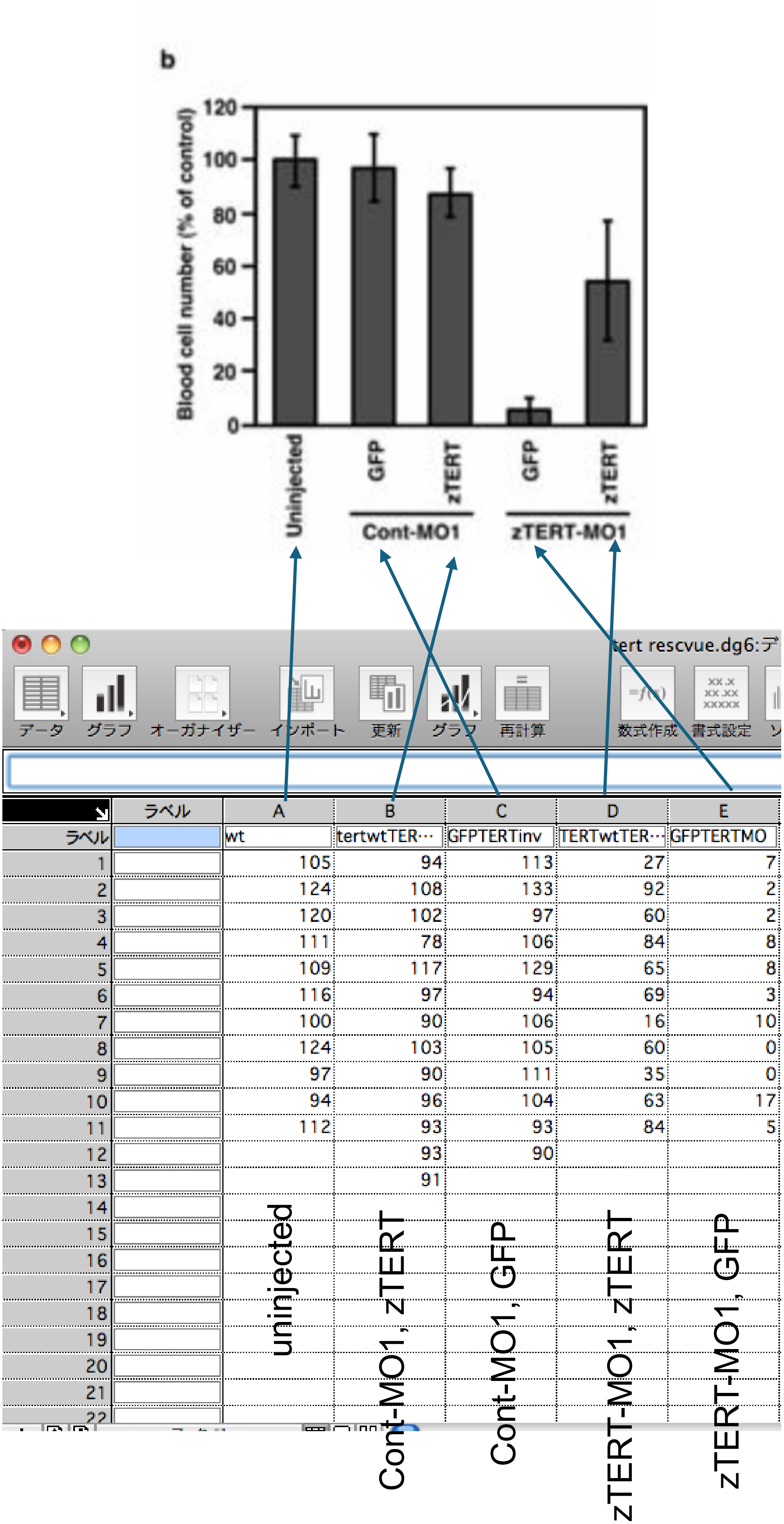

tert rescvue.dg6:デ

データ グラフ オーガナイザー インポート 更新 グラフ 再計算 数式作成 書式設定 ソ

	ラベル	A	B	C	D	E
ラベル		wt	tertwtTER···	GFPTERTinv	TERTwtTER···	GFPTERTMO
1		105	94	113	27	7
2		124	108	133	92	2
3		120	102	97	60	2
4		111	78	106	84	8
5		109	117	129	65	8
6		116	97	94	69	3
7		100	90	106	16	10
8		124	103	105	60	0
9		97	90	111	35	0
10		94	96	104	63	17
11		112	93	93	84	5
12			93	90		
13			91			
14						
15						
16						
17						
18						
19						
20						
21						
22						

uninjected

Cont-MO1, zTERT

Cont-MO1, GFP

zTERT-MO1, zTERT

zTERT-MO1, GFP

This graph shows the blood cell counts for each experimental group. Values are expressed relative to the uninjected control, which was set to 100.
