# Supplementary material for: Correction: A non-canonical function of zebrafish telomerase reverse transcriptase is required for developmental hematopoiesis
Source: PLoS One. 2026 May 29;21(5):e0350446. doi: 10.1371/journal.pone.0350446 (PMC13221052; doi:10.1371/journal.pone.0350446)
Supplement: S2 File — (PDF) [file pone.0350446.s002.pdf]

Fig.5F

Original data

runx

p53+/+
Cont-MO1

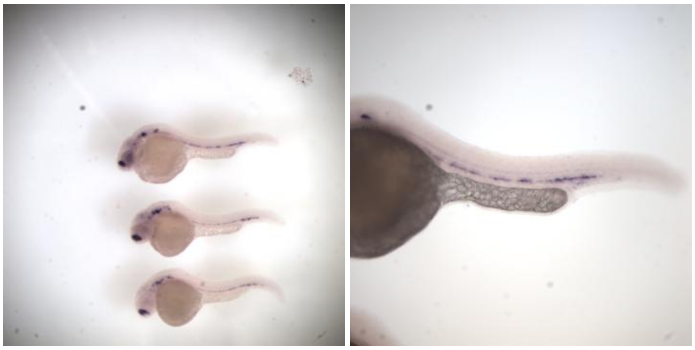

p53+/+
zTERT-MO1

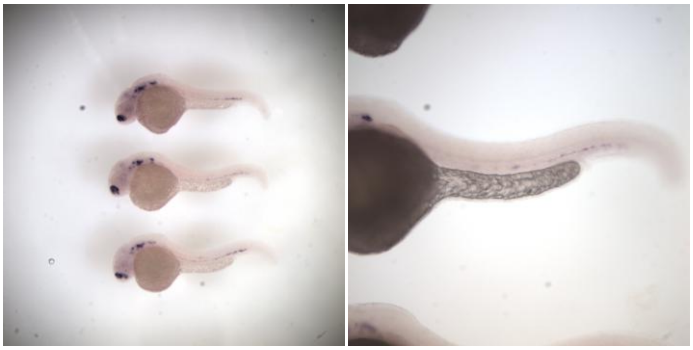

p53m/m
Cont-MO1

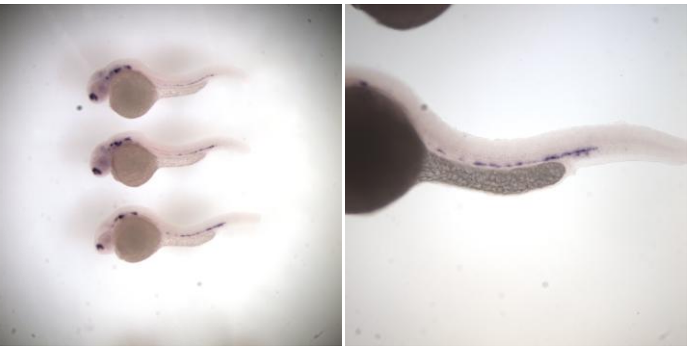

p53m/m
zTERT-MO1

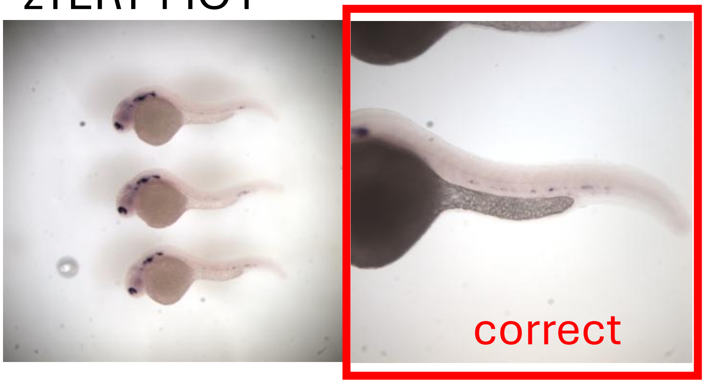

Fig.5B

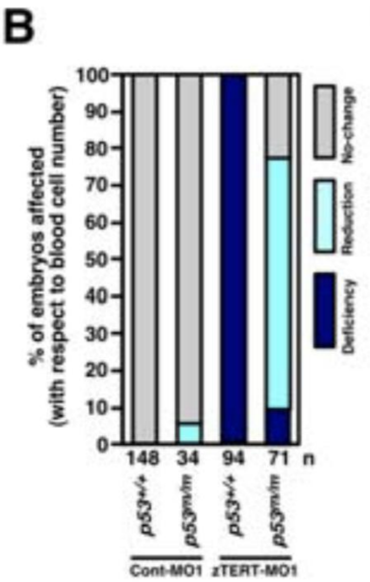

Deficiency

Total number

Completed deficiency

Significant deficiency

reduction

No-change

rescue TERT MO.dg6:データビュー

データ グラフ オーガナイザー インポート 更新 グラフ 再計算 数式作成 書式設定 ソート 行と列を転置

	ラベル	A	B	C	D	E	F	G
ラベル				complete d…	significant …	reduction	no-change	
1		GFP	344	0	0	0.3	99.7	
2		zTERT-cDNA	176	0	0	0.6	99.4	
3		zTERT-DN	116	0	0	0.9	99.1	
4		zTERT-del	55	0	0	5.5	94.5	
5		zTERT-del DN	63	0	0	0	100	
6		hTERT	159	0	0	0.6	99.4	
7		hTERT-DN	189	0	0	0	100	
8		GFP	222	57.2	33.8	7.7	1.4	
9		zTERT-cDNA	149	14.1	24.8	28.9	32.2	
10		zTERT-DN	65	69.2	23.1	6.2	1.5	
11		zTERT-del	60	21.7	28.3	26.7	23.3	
12		zTERT-del DN	88	47.7	43.2	9.1	0	
13		hTERT	104	5.8	38.5	41.3	14.4	
14		hTERT-DN	99	43.4	42.4	14.1	0	
15		wt	49	0	0	0	100	
16								
17		p53+/+	148	0	0	0	100	p53+/+, Cont-MO1
18		p53-/-	34	0	0	6	94	p53m/m, Cont-MO1
19		p53+/+	94	78.7	21.3	0	0	p53+/+, zTERT-MO1
20		p53-/-	71	0	10	68	22	p53m/m, zTERT-MO1
21								
22		TERT-cont+hBcl2	3	0	0	0	100	
23		TERT-cont+E1B	23	0	0	4.3	95.7	
24		TERT-MO+hBcl2	14	28.6	42.9	28.6	0	
25		TERT-MO+E1B	42	23.8	52.4	21.4	2.4	
26								
27		TERT-cont+hBcl2	157	0	0	0	157	
28		TERT-MO+hBcl2	119	16	51	35	17	
29		TERT-cont+E1B	166	0	0	1	165	
30		TERT-MO+E1B	145	23	68	40	14	
31								

Because the blood cells were counted under the microscope, photographs were not taken.

Fig.5C

C

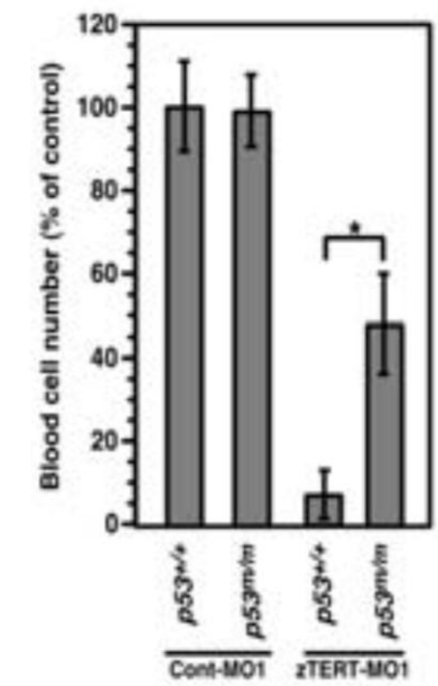

	average	std
p53+/+, Cont-MO1	102.5	10.937
p53m/m, Cont-MO1	101.3	8.895
p53+/+, zTERT-MO1	6.7	5.982
p53m/m, zTERT-MO1	49	12.092

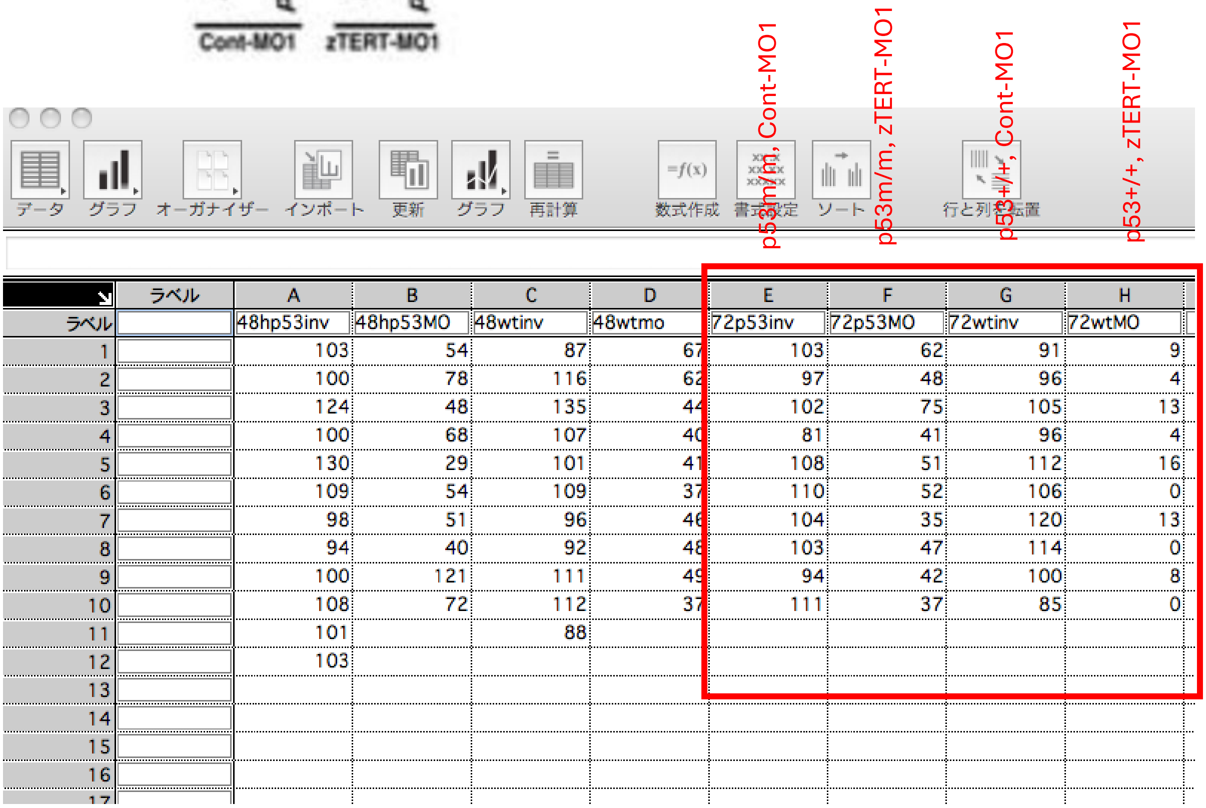

	ラベル	A	B	C	D	E	F	G	H
ラベル		48hp53inv	48hp53MO	48wtinv	48wtmo	72p53inv	72p53MO	72wtinv	72wtMO
1		103	54	87	67	103	62	91	9
2		100	78	116	62	97	48	96	4
3		124	48	135	44	102	75	105	13
4		100	68	107	40	81	41	96	4
5		130	29	101	41	108	51	112	16
6		109	54	109	37	110	52	106	0
7		98	51	96	46	104	35	120	13
8		94	40	92	48	103	47	114	0
9		100	121	111	49	94	42	100	8
10		108	72	112	37	111	37	85	0
11		101		88					
12		103							
13									
14									
15									
16									

Fig.5H

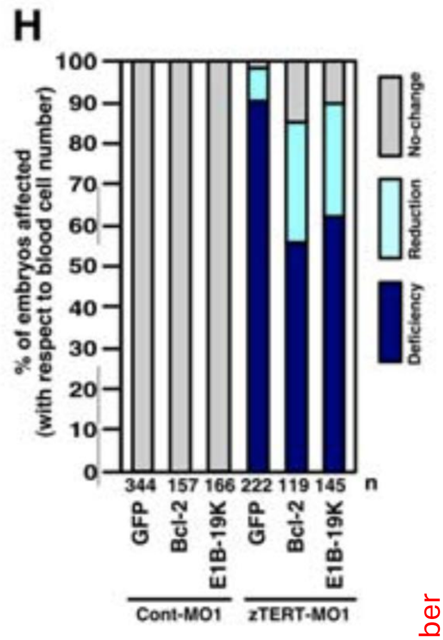

Deficiency

Total number
Completed deficiency
Significant deficiency
reduction
No-change

rescue TERT MO.qg6:データビュー

データ グラフ オーガナイザー インポート 更新 グラフ 再計算 数式作成 書式設定 ソート 行と列を転置

	ラベル	A	B	C	D	E	F	G
ラベル				complete d···	significant ···	reduction	no-change	
1		GFP	344	0	0	0.3	99.7	
2		zTERT-cDNA	176	0	0	0.6	99.4	
3		zTERT-DN	116	0	0	0.9	99.1	
4		zTERT-del	55	0	0	5.5	94.5	
5		zTERT-del DN	63	0	0	0	100	
6		hTERT	159	0	0	0.6	99.4	
7		hTERT-DN	189	0	0	0	100	
8		GFP	222	57.2	33.8	7.7	1.4	
9		zTERT-cDNA	149	14.1	24.8	28.9	32.2	
10		zTERT-DN	65	69.2	23.1	6.2	1.5	
11		zTERT-del	60	21.7	28.3	26.7	23.3	
12		zTERT-del DN	88	47.7	43.2	9.1	0	
13		hTERT	104	5.8	38.5	41.3	14.4	
14		hTERT-DN	99	43.4	42.4	14.1	0	
15		wt	49	0	0	0	100	
16								
17		p53+/+	148	0	0	0	100	
18		p53-/-	34	0	0	6	94	
19		p53+/+	94	78.7	21.3	0	0	
20		p53-/-	71	0	10	68	22	
21								
22		TERT-cont+hBcl2	3	0	0	0	100	
23		TERT-cont+E1B	23	0	0	4.3	95.7	
24		TERT-MO+hBcl2	14	28.6	42.9	28.6	0	
25		TERT-MO+E1B	42	23.8	52.4	21.4	2.4	
26								
27		TERT-cont+hBcl2	157	0	0	0	157	
28		TERT-MO+hBcl2	119	16	51	35	17	
29		TERT-cont+E1B	166	0	0	1	165	
30		TERT-MO+E1B	145	23	68	40	14	
31								

Cont-MO1, GFP

zTERT-MO1, GFP

Cont-MO1, Bcl-2
zTERT-MO1, Bcl-2
Cont-MO1, E1B-19K
zTERT-MO1, E1B-19K
